# Brain network integration dynamics are associated with loss and recovery of consciousness induced by sevoflurane

**DOI:** 10.1002/hbm.25405

**Published:** 2021-03-19

**Authors:** Andrea I. Luppi, Daniel Golkowski, Andreas Ranft, Rüdiger Ilg, Denis Jordan, David K. Menon, Emmanuel A. Stamatakis

**Affiliations:** ^1^ Division of Anaesthesia University of Cambridge Cambridge UK; ^2^ Department of Clinical Neurosciences University of Cambridge Cambridge UK; ^3^ Department of Neurology, Klinikum rechts der Isar Technische Universität München München Germany; ^4^ Department of Anaesthesiology and Intensive Care Medicine, Klinikum rechts der Isar Technische Universität München München Germany; ^5^ Department of Neurology Asklepios Clinic Bad Tölz Germany; ^6^ Wolfon Brain Imaging Centre University of Cambridge Cambridge UK

**Keywords:** anaesthesia, complexity, consciousness, dynamic functional connectivity, integration‐segregation, sevoflurane, small‐world network

## Abstract

The dynamic interplay of integration and segregation in the brain is at the core of leading theoretical accounts of consciousness. The human brain dynamically alternates between a sub‐state where integration predominates, and a predominantly segregated sub‐state, with different roles in supporting cognition and behaviour. Here, we combine graph theory and dynamic functional connectivity to compare resting‐state functional MRI data from healthy volunteers before, during, and after loss of responsiveness induced with different concentrations of the inhalational anaesthetic, sevoflurane. We show that dynamic states characterised by high brain integration are especially vulnerable to general anaesthesia, exhibiting attenuated complexity and diminished small‐world character. Crucially, these effects are reversed upon recovery, demonstrating their association with consciousness. Higher doses of sevoflurane (3% vol and burst‐suppression) also compromise the temporal balance of integration and segregation in the human brain. Additionally, we demonstrate that reduced anticorrelations between the brain's default mode and executive control networks dynamically reconfigure depending on the brain's state of integration or segregation. Taken together, our results demonstrate that the integrated sub‐state of brain connectivity is especially vulnerable to anaesthesia, in terms of both its complexity and information capacity, whose breakdown represents a generalisable biomarker of loss of consciousness and its recovery.

## Graphical abstract

We use functional MRI to study the effects of sevoflurane anaesthesia on the dynamics of integration and segregation in the human brain. We show that brain states characterised by high integration are especially vulnerable to general anaesthesia, in terms of both complexity and information capacity. Higher doses of sevoflurane also compromise the temporal balance of integration and segregation in the human brain.

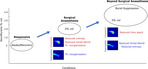

## 1 INTRODUCTION

The quest to understand the neural mechanisms of consciousness is a fundamental challenge of contemporary neuroscience. A powerful approach to this question is the combination of non‐invasive brain imaging with the temporary suppression of consciousness induced by general anaesthesia. In particular, recent work has demonstrated that the dynamics of brain network integration and segregation are of considerable relevance for our understanding of human consciousness and its loss (Northoff, Wainio‐Theberge, & Evers, 2020). On one hand, studies using dynamic functional connectivity (dFC) have increasingly demonstrated that patterns of brain connectivity are not stationary, but rather vary over time, switching between dynamic sub‐states with different relevance for cognition and even consciousness (Allen et al., 2014; Barttfeld et al., 2015; Demertzi et al., 2019; Huang, Zhang, Wu, Mashour, & Hudetz, 2020; Luppi et al., 2019, 2021; Lurie et al., 2020; Uhrig et al., 2018).

On the other hand, integration and segregation are essential properties of both the mind and the brain. Subjectively, humans experience the world as a whole (integration) composed of distinct sensory streams (segregation) (Tononi, 2004). Neurobiologically, information processed in parallel by domain‐specific systems must eventually be brought together and integrated, in order to guide adaptive behaviour (Dehaene & Changeux, 2011; Dehaene, Changeux, & Naccache, 2011; Luppi, Mediano, Rosas, Holland, et al., 2020; Mashour, Roelfsema, Changeux, & Dehaene, 2020). Therefore, the interplay of brain integration and segregation is at the core of leading theoretical accounts of consciousness (Balduzzi & Tononi, 2008; Deco, Tononi, Boly, & Kringelbach, 2015; Tononi & Edelman, 1998; Tononi, Sporns, & Edelman, 1994).

By representing the brain as a network of nodes (brain regions) and their connections, graph theory provides a formal way to quantify and investigate integration, segregation and their dynamic instantiation in the brain. Through this approach, Shine and colleagues (Shine et al., 2016) demonstrated that the human brain can be robustly characterised as alternating between an integrated sub‐state, and a segregated one. These sub‐states play different roles in supporting cognitive and motor functions (Shine et al., 2016), exhibit different relationships with the underlying anatomical connectivity (Fukushima et al., 2018), and they are also differentially modulated by the effects of the potent psychedelic, LSD (Luppi et al., 2021). Leveraging the same framework of dynamic integration and segregation, Luppi et al. (2019) recently showed that dynamic sub‐states dominated by integration or segregation have different relevance for consciousness: specifically, the dynamic sub‐state where integration predominates was found to exhibit concomitant reductions in network complexity and information capacity when consciousness is lost, whether due to propofol anaesthesia or severe brain injury (Luppi et al., 2019). The two dynamic sub‐states were also found to exhibit different patterns of reorganisation of functional connectivity during loss of consciousness, regardless of its cause: in particular, the anticorrelations between the brain's default mode and executive control networks, which robustly characterise the conscious brain at rest (Buckner & DiNicola, 2019; Fox et al., 2005; Raichle et al., 2001) were abolished specifically during the integrated sub‐state (Luppi et al., 2019).

Crucially, in addition to being compromised when consciousness is lost, any aspects of brain function that actively support consciousness should also be restored when consciousness returns. Here, we combined graph theory with dynamic connectivity in previously published functional MRI data (*N* = 16 healthy volunteers) (Golkowski et al., 2019; Ranft et al., 2016) to explore the time‐resolved effects of loss of consciousness and its recovery (indicated by loss and recovery of responsiveness) resulting from general anaesthesia with the inhalational anaesthetic, sevoflurane. Sevoflurane reversibly induces loss of consciousness in humans, and is widely used in clinical practice. In the past decade, there have been growing efforts to understand its effects on the human brain (Blain‐Moraes et al., 2017; Deshpande, Kerssens, Sebel, & Hu, 2010; Huang et al., 2016; Kafashan, Ching, & Palanca, 2016; Martuzzi, Ramani, Qiu, Rajeevan, & Constable, 2010; Martuzzi et al., 2011; Nir et al., 2020; Palanca, Avidan, & Mashour, 2017; Palanca et al., 2015; Riehl, Palanca, & Ching, 2017; J. Zhang et al., 2018). However, the specific dynamics of brain network integration and segregation under sevoflurane anaesthesia remain unexplored. Specifically, here we hypothesised that if the effects on brain integration and segregation observed with propofol anaesthesia and patients with disorders of consciousness (DOC) (Luppi et al., 2019) are involved in supporting the presence of consciousness in the human brain, then (a) they should generalise to sevoflurane‐induced anaesthesia; and (b) these effects should be reversed upon recovery, since any aspects of brain function that actively support consciousness should be restored when consciousness returns.

## 2 MATERIALS AND METHODS

The present study was a re‐analysis of a previously acquired dataset, which is described in detail in the original publication (Ranft et al., 2016). Although the original study acquired both functional MRI (fMRI) and electroencephalographic (EEG) data, in the present work we only considered the fMRI data.

### 2.1 Study participants

The ethics committee of the medical school of the Technische Universität München (München, Germany) approved the current study, which was conducted in accordance with the Declaration of Helsinki. Written informed consent was obtained from volunteers at least 48 hr before the study session. Twenty healthy adult men (20–36 years of age; mean, 26 years) were recruited through campus notices and personal contact, and compensated for their participation in the study.

Before inclusion in the study, detailed information was provided about the protocol and risks, and medical history was reviewed to assess any previous neurologic or psychiatric disorder. A focused physical examination was performed, and a resting electrocardiogram was recorded. Further exclusion criteria were the following: physical status other than American Society of Anesthesiologists physical status I, chronic intake of medication or drugs, hardness of hearing or deafness, absence of fluency in German, known or suspected disposition to malignant hyperthermia, acute hepatic porphyria, history of halothane hepatitis, obesity with a body mass index more than 30 kg/m^2^, gastrointestinal disorders with a disposition for gastroesophageal regurgitation, known or suspected difficult airway, and presence of metal implants. Data acquisition took place between June and December 2013.

### 2.2 Study protocol

Sevoflurane concentrations were chosen so that subjects tolerated artificial ventilation (reached at 2.0 vol%) and that burst‐suppression (BS) was reached in all participants (around 4.4 vol%). To make group comparisons feasible, an intermediate concentration of 3.0 vol% was also used. In the MRI scanner, volunteers were in a resting state with eyes closed for 700 s. Since EEG data were simultaneously acquired during MRI scanning (Ranft et al., 2016) (though they are not analysed in the present study), visual online inspection of the EEG was used to verify that participants did not fall asleep during the pre‐anaesthesia baseline scan. Sevoflurane mixed with oxygen was administered via a tight‐fitting facemask using an fMRI‐compatible anaesthesia machine (Fabius Tiro, Dräger, Germany). Standard American Society of Anesthesiologists monitoring was performed: concentrations of sevoflurane, oxygen and carbon dioxide, were monitored using a cardiorespiratory monitor (DatexaS/3, General electric). After administering an end‐tidal sevoflurane concentration (etSev) of 0.4 vol% for 5 min, sevoflurane concentration was increased in a stepwise fashion by 0.2 vol% every 3 min until the participant became unconscious, as judged by the loss of responsiveness (LOR) to the repeatedly spoken command “squeeze my hand” two consecutive times. Sevoflurane concentration was then increased to reach an end‐tidal concentration of approximately 3 vol%. When clinically indicated, ventilation was managed by the physician and a laryngeal mask suitable for fMRI (I‐gel, Intersurgical, United Kingdom) was inserted. The fraction of inspired oxygen was then set at 0.8, and mechanical ventilation was adjusted to maintain end‐tidal carbon dioxide at steady concentrations of 33 ± 1.71 mmHg during BS, 34 ± 1.12 mmHg during 3 vol%, and 33 ± 1.49 mmHg during 2 vol% (throughout this article, mean ± *SD*). Norepinephrine was given by continuous infusion (0.1 ± 0.01 μg kg^−1^ min^−1^) through an intravenous catheter in a vein on the dorsum of the hand, to maintain the mean arterial blood pressure close to baseline values (baseline, 96 ± 9.36 mmHg; BS, 88 ± 7.55 mmHg; 3 vol%, 88 ± 8.4 mmHg; 2 vol%, 89 ± 9.37 mmHg; follow‐up, 98 ± 9.41 mmHg). After insertion of the laryngeal mask airway, sevoflurane concentration was gradually increased until the EEG showed BS with suppression periods of at least 1,000 ms and about 50% suppression of electrical activity (reached at 4.34 ± 0.22 vol%), which is characteristic of deep anaesthesia. At that point, another 700 s of electroencephalogram and fMRI was recorded. Further 700 s of data were acquired at steady end‐tidal sevoflurane concentrations of 3 and 2 vol%, respectively, each after an equilibration time of 15 min. In a final step, etSev was reduced to two times the concentration at LOR. However, most of the subjects moved or did not tolerate the laryngeal mask any more under this condition: therefore, this stage was not included in the analysis (Ranft et al., 2016).

Sevoflurane administration was then terminated, and the scanner table was slid out of the MRI scanner to monitor post‐anaesthetic recovery. The volunteer was manually ventilated until spontaneous ventilation returned. The laryngeal mask was removed as soon as the patient opened his mouth on command. The physician regularly asked the volunteer to squeeze their hand: recovery of responsiveness was noted to occur as soon as the command was followed. Fifteen minutes after the time of recovery of responsiveness, the Brice interview was administered to assess for awareness during sevoflurane exposure; the interview was repeated on the phone the next day.

After a total of 45 min of recovery time, another resting‐state combined fMRI‐EEG scan was acquired (with eyes closed, as for the baseline scan). When participants were alert, oriented, cooperative, and physiologically stable, they were taken home by a family member or a friend appointed in advance. A total of *N* = 16 volunteers completed all five stages (awake, BS, 3% vol, 2% vol, and recovery) and were included in our analyses.

### 2.3 FMRI acquisition

Data acquisition was carried out on a 3‐Tesla magnetic resonance imaging scanner (Achieva Quasar Dual 3.0 T 16CH, The Netherlands) with an eight‐channel, phased‐array head coil. The data were collected using a gradient echo planar imaging sequence (echo time = 30 ms, repetition time [TR] = 1.838 s, flip angle = 75°, field of view = 220 × 220 mm^2^, matrix = 72 × 72, 32 slices, slice thickness = 3 mm, and 1 mm interslice gap; 700‐s acquisition time, resulting in 350 functional volumes). The anatomical scan was acquired before the functional scan using a T1‐weighted MPRAGE sequence with 240 × 240 × 170 voxels (1 × 1 × 1 mm voxel size) covering the whole brain.

### 2.4 Preprocessing

The preprocessing and image analysis were performed using the CONN toolbox, version 17f (CONN; http://www.nitrc.org/projects/conn) (Whitfield‐Gabrieli & Nieto‐Castanon, 2012) based on Statistical Parametric Mapping 12 (http://www.fil.ion.ucl.ac.uk/spm), implemented in MATLAB 2016a. For each condition (awake, BS, 3% vol, 2% vol sevoflurane, and recovery), we applied a standard preprocessing pipeline, the same as we employed in our previous studies (Luppi et al., 2019, 2021). The pipeline involved the following steps: removal of the first three volumes, to achieve steady‐state magnetisation; motion correction; slice‐ timing correction; identification of outlier volumes for subsequent scrubbing by means of the quality assurance/artifact rejection software ART (http://www.nitrc.org/projects/artifact_detect); normalisation to Montreal Neurological Institute (MNI‐152) standard space (2 mm isotropic resampling resolution), using the segmented grey matter image from each volunteer's T1‐weighted anatomical image, together with an a priori grey matter template; finally, the functional data were spatially smoothed with a Gaussian kernel of 6 mm full width at half‐maximum. For outlier detection, we adopted the default CONN settings of 5 global signal *Z*‐values and 0.9 mm.

### 2.5 Denoising

Denoising was also performed using the CONN toolbox. To reduce noise due to cardiac and motion artifacts, which are known to impact functional connectivity and network analyses (Power, Barnes, Snyder, Schlaggar, & Petersen, 2012; van Dijk, Sabuncu, & Buckner, 2012), we applied the anatomical CompCor method of denoising the functional data (Behzadi, Restom, Liau, & Liu, 2007), as implemented within the CONN toolbox. The aCompCor method involves regressing out of the functional data the first five principal components attributable to white matter and cerebrospinal fluid (CSF) signal; six subject‐specific realignment parameters (three translations and three rotations) as well as their first‐order temporal derivatives; followed by scrubbing our outliers identified by ART, using ordinary least squares regression (Whitfield‐Gabrieli & Nieto‐Castanon, 2012). Finally, the denoised BOLD signal timeseries were linearly detrended and band‐pass filtered to eliminate both low‐frequency drift effects and high‐frequency noise, thus retaining frequencies between 0.008 and 0.09 Hz.

The step of global signal regression (GSR) has received substantial attention in the literature as a denoising method (Andellini, Cannatà, Gazzellini, Bernardi, & Napolitano, 2015; Lydon‐Staley, Ciric, Satterthwaite, & Bassett, 2019; Power et al., 2014). GSR mathematically mandates that approximately 50% of correlations between regions will be negative (Braun et al., 2012); however, the proportion of anticorrelations between brain regions has been shown to vary across states of consciousness, including DOC as well as anaesthesia with both propofol and sevoflurane (Golkowski et al., 2019; Luppi et al., 2019; Ranft et al., 2016). Indeed, recent work has demonstrated that the global signal contains information about states of consciousness, including anaesthesia and DOC (Tanabe et al., 2020). Therefore, in line with previous studies, including investigations of general anaesthesia (Carhart‐Harris et al., 2016; Luppi et al., 2019) here we decided to avoid GSR in favour of the aCompCor denoising procedure, which is among those recommended for investigations of dynamic connectivity (Lydon‐Staley et al., 2019). The cartographic profile method employed here to identify integrated and segregated sub‐states of dFC (see below) has also been shown to be robust to the use of GSR during preprocessing (Shine et al., 2016).

### 2.6 Definition on regions of interest

To construct matrices of functional connectivity, spatially normalised brains were parcellated into 200 cortical regions of interest (ROIs), obtained from the scale‐200 version of the recent multi‐scale local–global functional parcellation of Schaefer et al. (2018). Since this parcellation only includes cortical regions, it was augmented with 32 subcortical ROIs from the highest resolution of the recent Melbourne subcortical functional parcellation (Tian, Margulies, Breakspear, & Zalesky, 2020), following recent work (Luppi & Stamatakis, 2020). We refer to this composite 232‐ROI parcellation as the “augmented Schaefer‐232.”

We chose this atlas because recent work has demonstrated that it produces networks whose topology is highly representative across alternative node definition schemes (Luppi & Stamatakis, 2020). Nevertheless, to ensure the robustness of our analyses to the choice of parcellation, we replicated them using the Brainnetome atlas (Fan et al., 2016), which comprises 246 cortical and subcortical ROIs, derived from multimodal (anatomical and functional) connectivity. Parcellations in the order of 200 nodes were found to produce the most topologically representative networks, with the Brainnetome atlas being the second most representative after Schaefer‐232 (Luppi & Stamatakis, 2020). Results presented in the main text pertain to the Schaefer‐232 atlas, with corresponding results for the alternative parcellation presented in the Supporting Information. For each ROI, the time‐courses of denoised BOLD signals were averaged between all voxels belonging to it, and extracted for further analysis.

### 2.7 Dynamic functional connectivity

Dynamic connectivity matrices were derived using an overlapping sliding‐window approach (Allen et al., 2014; Barttfeld et al., 2015). For each subject and each condition (awake, BS, 3% vol, 2% vol, and recovery), tapered sliding windows were obtained by convolving a rectangle of 22 TRs (40s) with a Gaussian kernel of 3 TRs, sliding with 1 TR step size (Allen et al., 2014), in line with previous work (Luppi et al., 2019, 2021). The chosen length is in the range of 30–60 s recommended to capture spontaneous fluctuations while ensuring a sufficient number of timepoints per window to enable stable network identification; likewise, tapered windows are chosen to minimise the potential effects of outliers (Preti, Bolton, & Van De Ville, 2017). To ensure the robustness of our analyses to window length, we also replicated them with a shorter length (~33 s — 18 TRs) and a longer length (~50 s — 27 TRs). Within each of the resulting overlapping temporal windows of 22 (resp. 18, 27) TRs, a 232‐by‐232 matrix of functional connectivity between ROIs was estimated (or 246‐by‐246 for the Brainnetome atlas). Hence, for each condition of each subject we obtained a 3D tensor, consisting of one functional connectivity matrix for each timepoint.

### 2.8 Derivation of integrated and segregated sub‐states

Previous work on both healthy and unconscious individuals has established the “cartographic profile” as a robust method to investigate the dynamics of network integration and segregation in the human brain (Fukushima et al., 2018; Luppi et al., 2019, 2021; Shine et al., 2016). This methods identifies dynamic sub‐states of higher integration or segregation, with different roles in human behaviour and consciousness (Fukushima et al., 2018; Luppi et al., 2019, 2021; Shine et al., 2016). Since the cartographic profile relies on the graph‐theoretical module assignments of each ROI (node), here, nodes were given by the ROIs of a given parcellation scheme, with network edges given by their functional connectivity. Under these conditions, modules are defined as groups of nodes that are positively correlated with each other, but negatively correlated with nodes belonging to different modules (Sporns & Betzel, 2016).

**FIGURE 1:**
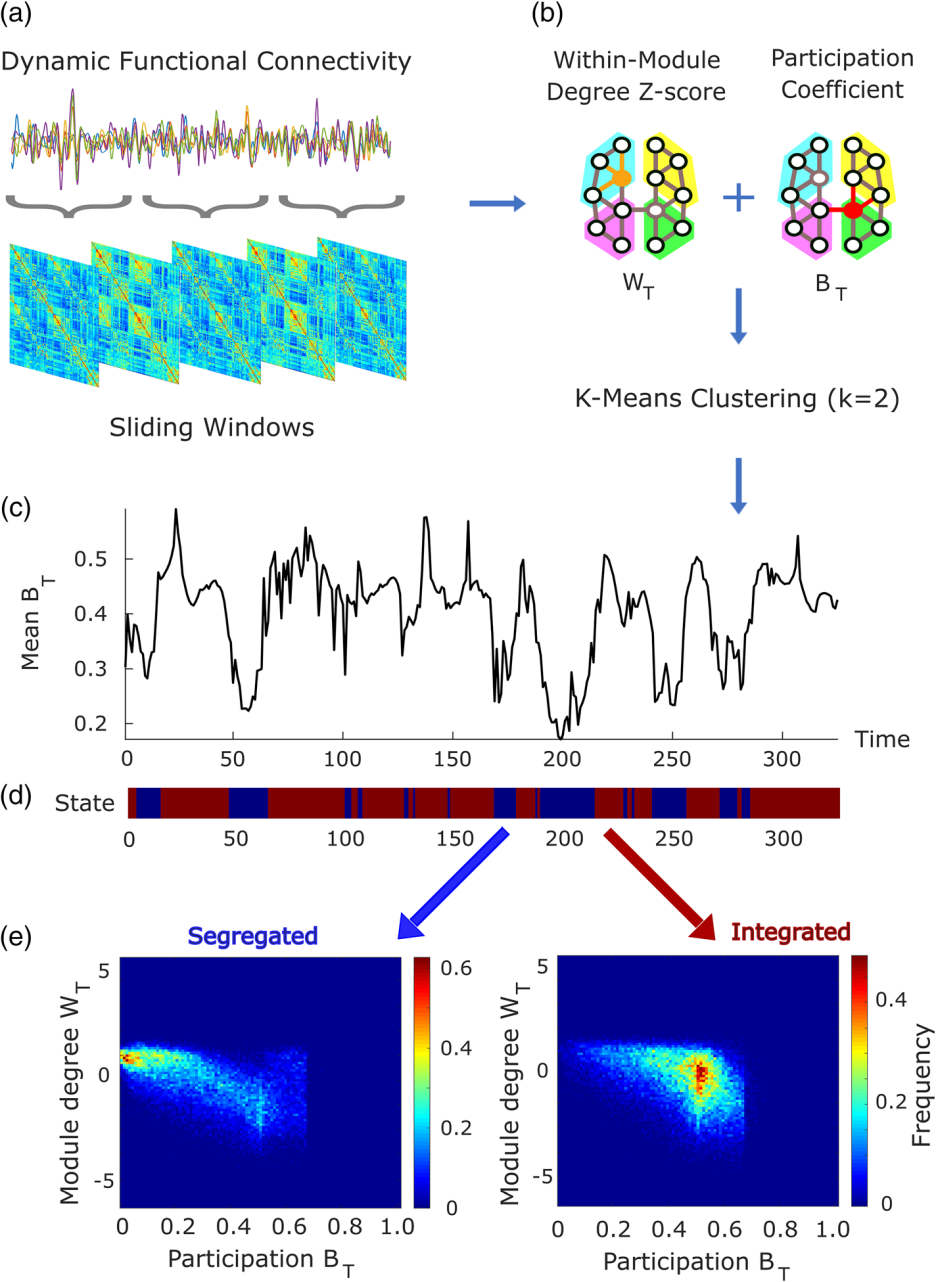
Derivation of predominantly integrated and segregated sub‐states of dynamic functional connectivity from cartographic profile. (a) Dynamic functional connectivity is obtained from overlapping sliding windows. (b), the graph‐theoretical properties of participation coefficient *B*_T_ and within‐module degree Z‐score *W*_T_ are used for *k*‐means clustering (with *k =* 2; note that *k* = 2 was also the best data‐driven clustering solution based on the silhouette criterion for quality of clustering). The cluster with higher (lower) average participation coefficient is then identified as the predominantly integrated (segregated) dynamic sub‐state, separately for each condition. (c–d) Time series of the mean participation coefficient *B*_T_ for a representative subject at baseline, with the corresponding states assigned from k‐means clustering (blue: “predominantly segregated” and red: “predominantly integrated”). (d) Mean cartographic profile of both the predominantly segregated and predominantly integrated states from one representative subject

We then followed the well‐established procedure for determining the cartographic profile, developed in Shine et al. (2016) and also adopted in our previous studies (Luppi et al., 2019, 2021) (Figure 1). Detailed descriptions are provided in those previous publications and in the Materials and Methods section of Supporting Information. Briefly, for each time‐point of each subject and condition, the Louvain greedy algorithm (Blondel, Guillaume, Lambiotte, & Lefebvre, 2008) implemented in the Brain Connectivity Toolbox (BCT; http://www.brain-connectivity-toolbox.net) (Rubinov & Sporns, 2010, 2011) was used to identify network modules. Due to its stochastic nature, the algorithm was repeated for 100 iterations for each time‐ resolved network, and the module size resolution parameter *γ* was set to one, the default (Fukushima et al., 2018; Luppi et al., 2019, 2021; Shine et al., 2016).

Based on the modularity assignments, we then used the BCT to derive the participation coefficient and within‐degree Z‐score for each node (Materials and Methods in Supporting Information): together, these two measures quantify both a node's *inter‐*modular and *intra‐*modular connectivity. Based on their joint histogram, the “cartographic profile” was determined following previously established procedures (using MATLAB code made freely available by Shine et al., 2016 at https://github.com/macshine/integration/) (Fukushima et al., 2018; Luppi et al., 2019, 2021; Shine et al., 2016). For each subject and condition, an unsupervised machine learning algorithm known as *k*‐means clustering was used to assign matrices of dFC into *k* = 2 clusters based on the joint histogram of participation coefficient and within‐module degree *Z*‐score (Shine et al., 2016). To avoid the possibility of the algorithm becoming stuck in local minima, it was repeated 500 times with random re‐initialisation of the two clusters' initial points. Following previous work (Luppi et al., 2019, 2021, Shine et al., 2016) Pearson correlation was chosen as distance metric for the algorithm. Although based on previous work our interest was a priori on clustering with *k* = 2, the clustering process was also repeated with values of *k* ranging between 2 and 7, using MATLAB's *evalclusters* function to determine the goodness of different clustering solutions using the Silhouette criterion (Figure S1).

Thus, for each subject the *k*‐means clustering algorithm assigned each matrix of dFC to one of *k* clusters. Finally, for our clustering of interest (with *k* = 2) we obtained the mean participation coefficient of the matrices belonging to each of the two clusters. Following the well‐established procedure defined in previous work with both healthy and unconscious individuals (Fukushima et al., 2018; Luppi et al., 2019; Shine et al., 2016), the cluster whose matrices had a higher mean participation coefficient was labelled as the “predominantly integrated” state, whereas the cluster with lower mean participation coefficient was considered to be the “predominantly segregated” state. For each subject, a centroid matrix of functional connectivity was computed for each state (predominantly integrated and segregated), as the element‐wise median of the timepoint‐specific FC matrices assigned to the cluster corresponding to that state. This procedure has been successfully applied in previous studies to obtain robust characterisation of predominantly integrated and segregated sub‐states of dFC, which have been well characterised in the literature in terms of their role in supporting cognitive and motor functions (Shine et al., 2016), relationship with the underlying anatomical connectivity (Fukushima et al., 2018), and relevance for supporting human consciousness (Luppi et al., 2019) and the time‐varying effects of the psychedelic LSD (Luppi et al., 2021). The proportion of time spent in each sub‐state was also quantified, as the number of timepoints assigned to that cluster, over the total number of timepoints.

### 2.9 Validation of dynamics against stationary null model

Using the cartographic profile method employed here (Shine et al., 2016), previously demonstrated that the resting brain fluctuates more frequently than a stationary null model, indicating the presence of genuine dynamics in the data. Nevertheless, here we also validated this observation in our own data, using a vector autoregressive (VAR) model to generate surrogate timeseries which are stationary by construction. Since fitting a single multi‐dimensional VAR that simultaneously considered the covariance between all pairs of regional timeseries was computationally infeasible, we followed previous work (Zalesky, Fornito, Cocchi, Gollo, & Breakspear, 2014), fitting two‐dimensional VAR models separately for each pair of ROIs of each participant in each condition. The VAR model order was set to 5, which was chosen to correspond to a temporal lag of ~9 s (given our TR of 1.83 s), similar to the 8 s lag used by Shine et al. (2016) and Zalesky et al. (2014). Sliding‐windows dFC followed by the cartographic profile was then applied to the surrogate data of each participant and condition, and the proportion of time spent in the predominantly integrated sub‐state was compared between the empirical data and the stationary surrogates.

### 2.10 Small‐world propensity

When investigating information‐processing networks, such as the human brain, it is advantageous to consider what kind of network structure would facilitate the exchange of information. A key property of many natural and artificial networks, including the human brain, is the so‐called “small‐world” organisation (Bassett & Bullmore, 2017) (but see Papo, Zanin, Martínez, & Buldú, 2016). A small‐world network combines the presence of tightly interconnected clusters (characterising lattice networks, and theorised to support specialised processing) with a short characteristic path length (a key feature of random network, facilitating integration between different clusters). Thus, small‐worldness represents a mark of optimal balance between global and local processing. Small‐worldness of dynamic sub‐states has been shown to decrease during anaesthesia and in patients with DOC, specifically during the integrated sub‐state (Luppi et al., 2019). Therefore, we hypothesised that the same should be observed with sevoflurane.

We adopted the measure of small‐world propensity recently developed by Muldoon, Bridgeford, and Bassett (2016), which provides a theoretically principled way to quantify and compare the extent that different networks exhibit small‐world structure, while accounting for network density. The small‐world propensity, *φ*, is designed to quantify the extent that a network displays small‐world organisation by taking into account the deviation of the network's empirically observed clustering coefficient, *C*_obs_, and characteristic path length, *L*_obs_, from equivalent lattice (*C*_latt_, *L*_latt_) and random (*C*_rand_, *L*_rand_) networks (Materials and Methods in Supporting Information).

(1) $$\varphi =1-\sqrt{\frac{{∆}_{C}^{2}+{∆}_{L}^{2}}{2}}$$

where

(2) $${∆}_{C}=\frac{C_{\text{latt}}-C_{\mathrm{obs}}}{C_{\text{latt}}-C_{\mathrm{rand}}}$$

(3) $${∆}_{L}=\frac{L_{\mathrm{obs}}-L_{\mathrm{rand}}}{L_{\text{latt}}-L_{\mathrm{rand}}}$$

Thus, ∆_*C*_ and ∆_*L*_ quantify the fractional deviation of the empirically observed clustering coefficient and characteristic path length, from the corresponding null models according to the definition of a small‐world network: namely, a lattice network for the clustering coefficient, and a random network for the characteristic path length (Muldoon et al., 2016).

Following Muldoon et al. (2016), we further bound both measures of fractional deviation ∆_*C*_ or ∆_*L*_ between 0 and 1 (to account for the possibility of empirical networks exceeding the corresponding null models), by setting negative values of ∆_*C*_ or ∆_*L*_ to 0, and values that exceed unity to be exactly 1. In turn, this ensures that the resulting values of small‐world propensity will also be bounded between 0 and 1 (Luppi et al., 2021).

Small‐world propensity is then interpreted as follows: both a large ∆_*C*_ or ∆_*L*_ would indicate large deviation of the network's properties from the corresponding properties that define small‐world organisation. Thus, large ∆_*C*_ or ∆_*L*_ would lead to the measure of small‐world propensity becoming closer to zero. Conversely, if a network exhibits both the high clustering coefficient of a lattice, and the low path length of a random network (thereby satisfying both requirements of the small‐world network definition), then it will have low ∆_*C*_ and low ∆_*L*_, and the small‐world propensity as a whole will be closer to 1. Hence, higher small‐world propensity intuitively indicates better adherence to the requirements of a small‐world network.

Unlike the small‐world index of Humphries and Gurney (2008), small‐world propensity is not intended as a way to determine, in absolute terms, whether or not a network exhibits small‐world structure. Rather, this metric is better suited to compare on a continuous scale the degree of small‐world organisation exhibited by different networks (Muldoon et al., 2016); thus, it is ideally suited for the purposes of the present study. Since the measure of small‐world propensity adopted here can be applied on weighted networks, we also repeated our analysis including all non‐negative edges (negative edges were set to zero because this measure depends on calculations of shortest paths between nodes, and therefore requires graphs with strictly positive edges [Rubinov & Sporns, 2010, 2011]).

### 2.11 Connectivity entropy

We also quantified the complexity of the pattern of connectivity of each sub‐state of functional connectivity, in terms of the mean normalised Shannon entropy of the connections of each ROI (“connectivity entropy”) (Saenger et al., 2018). Entropy is a measure of non‐uniformity of a distribution, with higher entropy reflecting a pattern that is less predictable and more diverse.

Here, we assigned the connectivity values in each column of the matrix into *n* bins to construct a distribution; we then computed the Shannon entropy of this distribution for each node, following the method of Saenger et al. (2018):

(4) $$H=-\sum_{i=1}^{n}p_{i}\log\left(p_{i}\right)/\log\left(n\right)$$

This measure of entropy was normalised to lie between 0 and 1 by dividing it by the Shannon entropy of a uniform distribution, which corresponds to log(*n*). Here, we followed previous work (Luppi et al., 2019; Saenger et al., 2018), always using *n* = 10 bins.

### 2.12 Statistical analysis

Since an analysis of variance revealed a significant effect of sevoflurane level on the percentage of scrubbed volumes (*F*[4,75] = 2.53, *p* = .047), the percentage of scrubbed volumes was included as a covariate of no interest, to statistically control for potential confounding effects. Moreover, one subject had over 20% of ART‐rejected volumes during the awake scan; therefore, we excluded this subject from analysis, leaving *N* = 15 subjects. The mean percentage (±*SD*) of scrubbed scans was 1.75 ± 1.96 (awake); 2.25 ± 3.17 (BS); 1.43 ± 2.37 (3% vol); 0.19 ± 0.75 (2% vol); 2.94 ± 2.70 (recovery).

The statistical significance of the effect of anaesthetic level was assessed with analysis of covariance; significant effects were further explored with Bonferroni‐corrected within‐subjects comparisons between each pair of anaesthetic doses.

To ensure the robustness of our results, we also repeated all analyses without including the covariates and without excluding the high‐artifact subject (Ranft et al., 2016): in this case, non‐parametric, permutation based testing (two‐sided within‐subjects *t* tests with 10,000 permutations) was used to ensure robustness to outliers; effect size was estimated using Cohen's *d*.

The network‐based statistic (NBS) approach (Zalesky, Fornito, & Bullmore, 2010) was used to investigate the statistical significance of sevoflurane‐induced alterations on the functional brain networks, for the predominantly integrated and segregated sub‐states. This nonparametric statistical method is designed to control the family‐wise error due to multiple comparisons, for application to graph data. Connected components of the graph are identified from edges that survive an a priori statistical threshold (*F* test; here we set the threshold to an intensity value of 10). In turn, the statistical significance of such connected components is estimated by comparing their topological extension against a null distribution of the size of connected components obtained from non‐parametric permutation testing. This approach rejects the null hypothesis on a component‐basis, and therefore achieves superior power compared to mass‐univariate approaches (Zalesky et al., 2010). Once again, the percentage of scrubbed volumes was included as a covariate of no interest to account for potential confounding effects. To ensure the robustness of our results, we also replicated them with an extent‐based threshold.

## 3 RESULTS

### 3.1 Reduced prevalence of integrated state under deep sevoflurane anaesthesia

**FIGURE 2:**
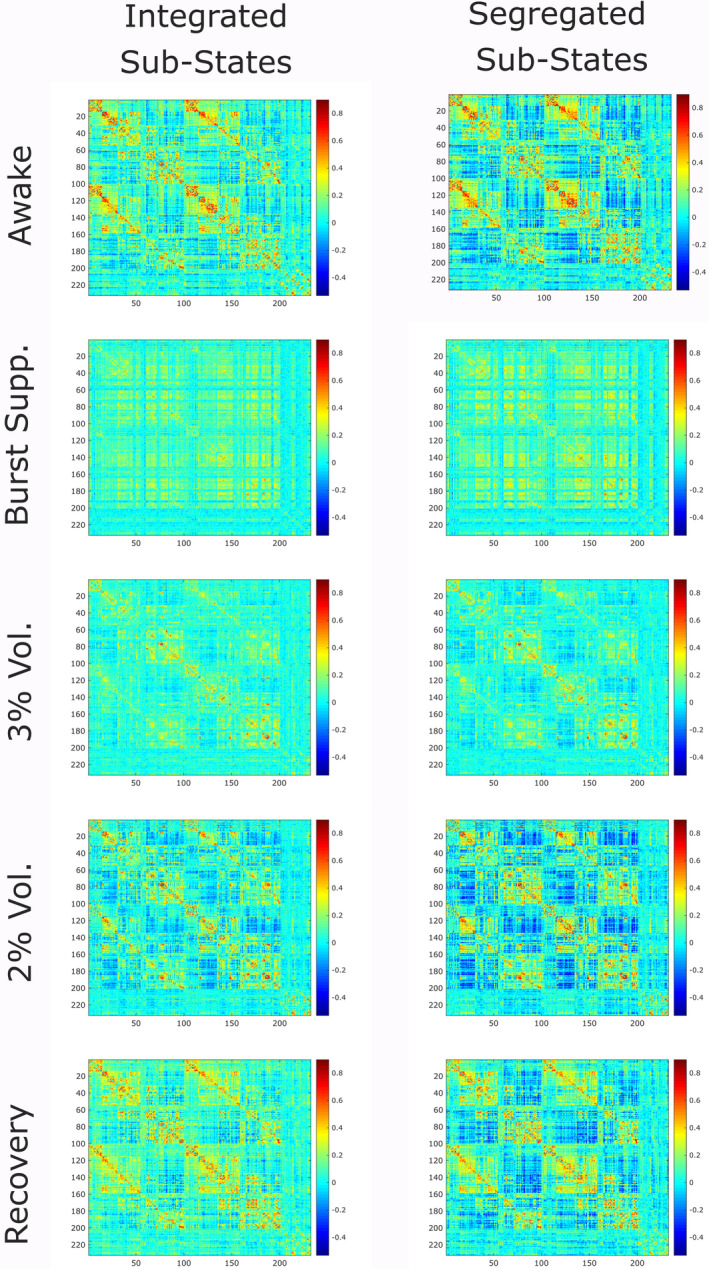
Group‐average centroids for the predominantly integrated (top) and segregated (bottom) sub‐states, for each condition. Matrices represent the group‐average of connectivity (Pearson correlation) between each pair of 232 cortical and subcortical regions from the Schaefer‐232 atlas, during each scanning condition, and separately for the integrated and segregated dynamic sub‐states. Note that the colour bar scale is equalised across matrices for ease of comparison

Studying alterations in brain function induced by sevoflurane offers a way to relate its potent anaesthetic effects to their underlying neurobiological correlates. In particular, the focus of this study was on the effects of general anaesthesia on the dynamics of two fundamental properties of the brain: integration and segregation. Thus, we employed an a priori clustering of dFC into two sub‐states: we followed a previously established methodology known as “cartographic profile,” which has been validated across several studies, to derive predominantly integrated and segregated sub‐states with well‐characterised roles in cognition and consciousness (Fukushima et al., 2018; Luppi et al., 2019, 2021; Shine et al., 2016) (Figure 2). Confirming the algorithm's capacity to robustly identify distinct clusters in the data, remarkable similarity can be seen between the clusters identified in this study (Figure 1d), and those shown in Figure 1 from Shine et al. (2016).

Reassuringly, in line with previous studies the silhouette criterion for quality of clustering (measured using MATLAB's *evalclusters* command) selected *k* = 2 as the most appropriate clustering of dFC across values of *k* ranging between 2 and 7 (Figure S1), for virtually every combination of subject and condition (including all subjects during the awake scan), thus providing data‐driven support for our hypothesis‐driven clustering of dFC into two sub‐states (predominantly integrated and segregated). Additionally, as expected within each condition the predominantly integrated sub‐state has significantly higher mean participation coefficient *B*_T_ than the predominantly segregated sub‐state (Figure S2).

The proportion of time spent in the predominantly integrated sub‐state during the awake condition (*M* = 0.66, *SD* = 0.16) was also consistent with previous results obtained by Shine et al. (2016) and by Luppi et al. (2019, 2021) using the same cartographic profile method, providing an additional sanity check on the key method underlying the present results (Figure 2 and Table S1). Note that the proportion of time spent in the primarily segregated sub‐state is just the complement of the proportion of time spent in the integrated sub‐state, since each timepoint belongs to either one or the other sub‐state.

**FIGURE 3:**
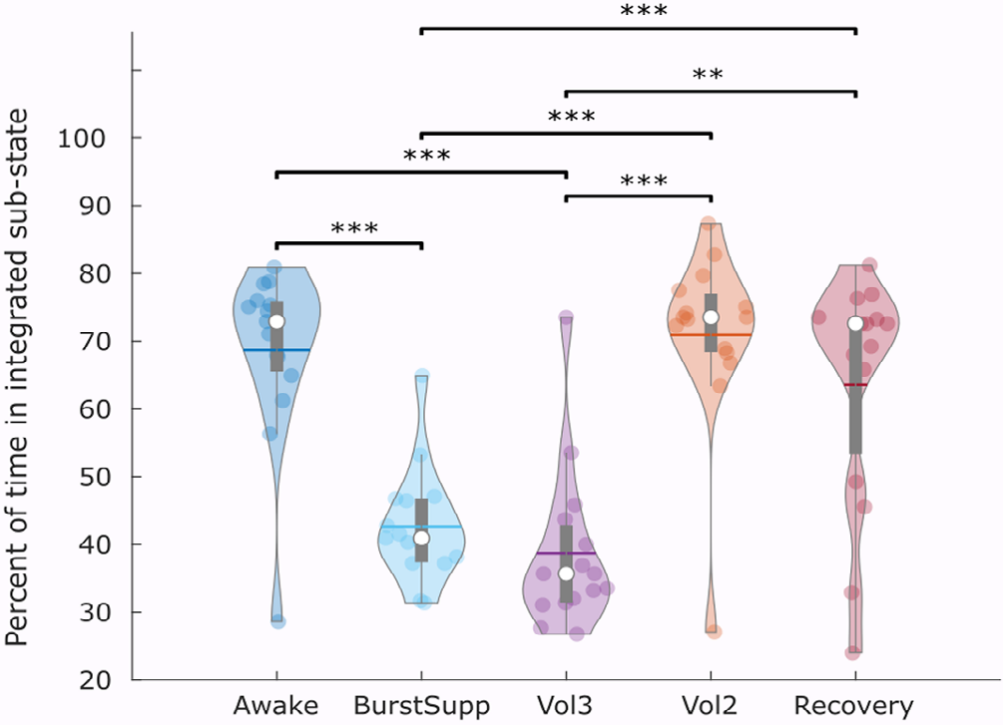
Reduced time is spent in the integrated sub‐state at deep levels of sevoflurane anaesthesia. Violin plots indicate the distribution of participants in each condition (coloured circles). White circle, median; horizontal centre line, mean; box limits, upper and lower quartiles; whiskers, 1.5× interquartile range. **p < .01; ***p < .001, Bonferroni‐corrected

However, an analysis of variance indicated a significant effect of anaesthetic dosage on the proportion of time spent in the predominantly integrated sub‐state (*F*[4,69] = 21.38, *p* < .001). Bonferroni‐corrected follow‐up paired tests revealed that time spent in the integrated state dropped significantly at the highest concentrations of sevoflurane (BS and 3% vol), returning to awake levels when sevoflurane concentration was reduced to 2% volume (Figure 3 and Table S1). We elaborate on these results in the Discussion.

Reassuringly, our validation analysis indicated that for both the awake condition and 2% vol sevoflurane, the proportion of time spent in the integrated sub‐state was significantly greater for empirical data than stationary data generated by a VAR model for each participant (Table S2). This was not the case for BS and 3% vol sevoflurane, consistently with substantial sevoflurane‐induced reorganisation of brain dynamics (we note that recovery, though not significantly different from wakefulness or 2% vol sevoflurane, included a number of outliers with unusually low time spent in the integrated sub‐state, which explains the lack of significant difference from the VAR model). We also note that during each condition, the proportion of time spent in the predominantly integrated sub‐state was significantly different from 50%, which is what we should have observed if sub‐states had been assigned randomly (Table S3).

### 3.2 Time‐specific effects of sevoflurane on brain connectivity

Prior research suggests that altered states of consciousness have differential neuronal underpinnings as observed at the macroscale with functional brain networks during the integrated and segregated sub‐states (Luppi et al., 2019, 2021). Thus, we sought to determine whether different effects of sevoflurane on brain function are manifested during dynamic sub‐states of high or low integration or segregation. We therefore investigated how each of these two sub‐states was affected by sevoflurane, in terms of its functional connectivity, as well as its network properties, given their proposed relevance for consciousness (Dehaene et al., 2011).

In particular, to identify network alterations that covaried with the presence versus absence of consciousness (as indicated by behavioural responsiveness), here we focused on connections between brain regions (edges) that satisfied *both* of the following conditions: (a) significant and consistent alteration in the absence of responsiveness: compared with wakefulness, a given connection should be significantly reduced during *all* three levels of sevoflurane (BS, 3% vol and 2% vol), or significantly increased during *all* three levels of sevoflurane; (b) any significant changes observed during LOR should be significantly and consistently reversed upon recovery.

We further refined our results to determine whether any of these specific connectivity alterations were sensitive to brain network dynamics, defined as connectivity changes that were only observed in one or the other dynamic sub‐state, but not both, as reported by Luppi et al. (2019) pertaining to both propofol anaesthesia and DOC.

**FIGURE 4:**
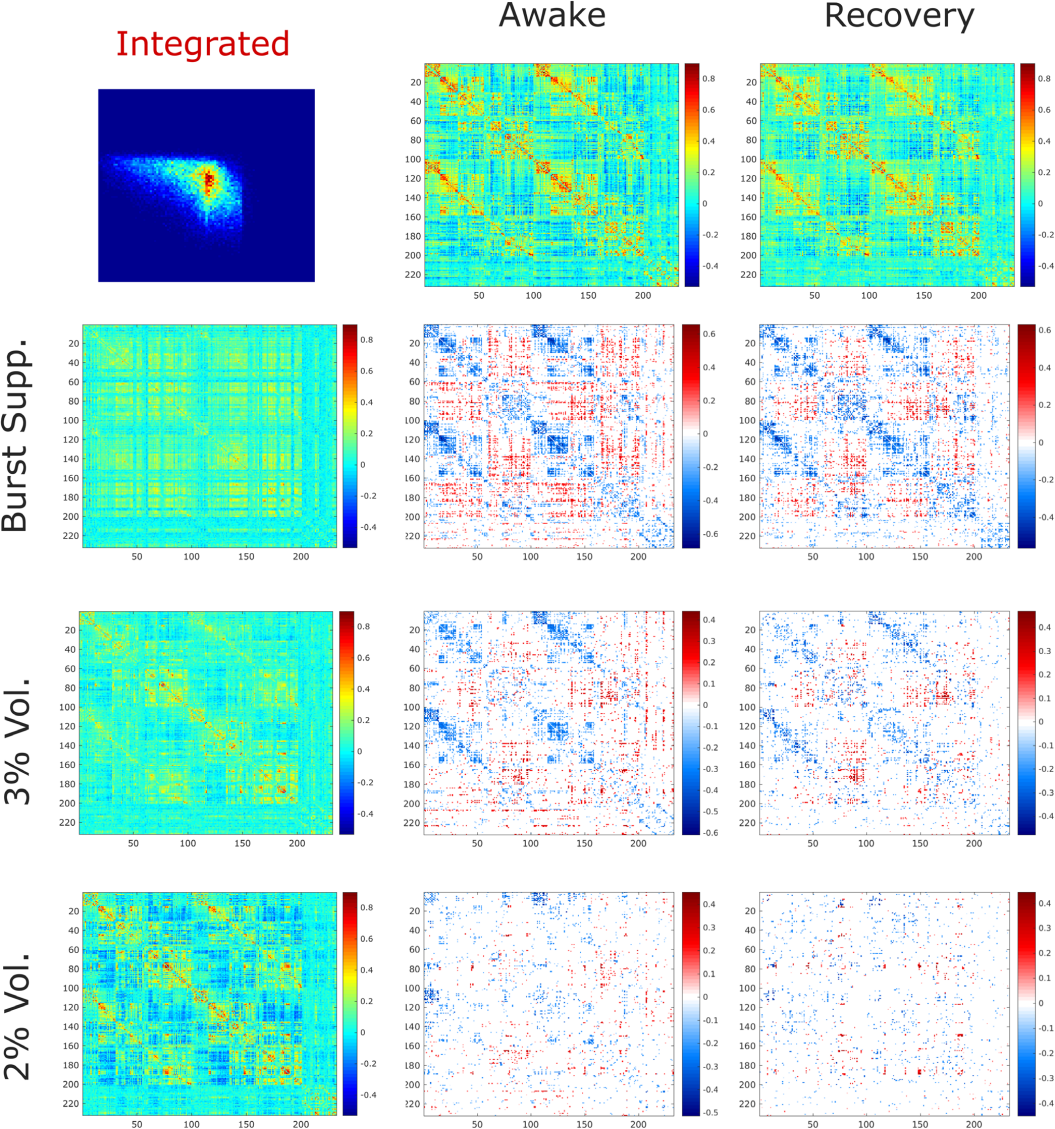
Connectivity changes during integrated sub‐state induced by sevoflurane. For the predominantly integrated sub‐state, functional connectivity between pairs of 232 brain regions from each conscious condition (awake and recovery) are compared with every unconscious condition (burst‐suppression, 3% sevoflurane and 2% sevoflurane), using the network‐based statistic (NBS) (Zalesky et al., 2010), to identify network alterations that reliably distinguish between the presence and absence of consciousness. Statistical significance between each pair of conditions was determined by the NBS with an intensity‐based F‐threshold of 10

**FIGURE 5:**
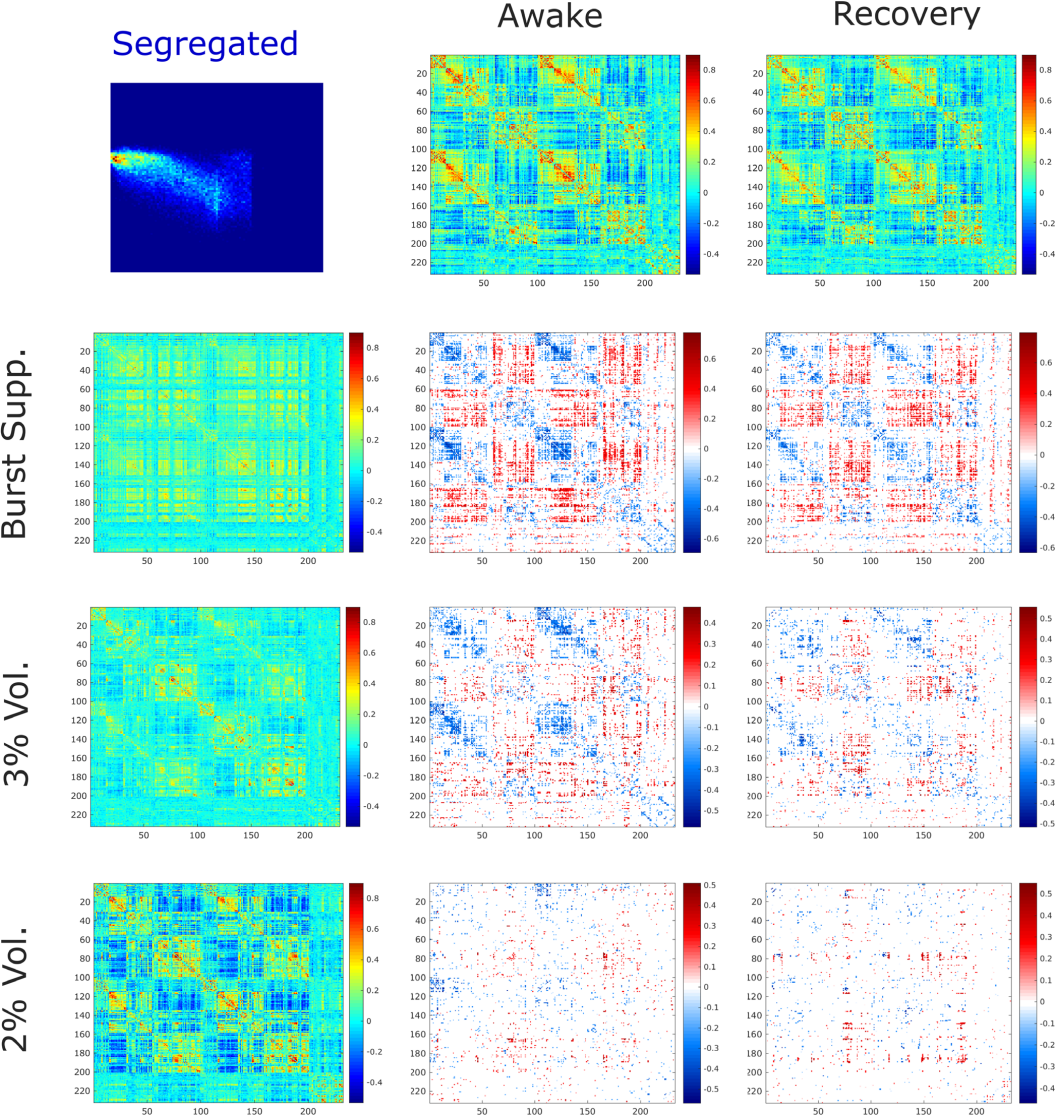
Connectivity changes during segregated sub‐state induced by sevoflurane. For the predominantly segregated sub‐state, functional connectivity between pairs of 232 brain regions from each conscious condition (awake and recovery) are compared with every unconscious condition (burst‐suppression, 3% sevoflurane and 2% sevoflurane), using the network‐based statistic (NBS) (Zalesky et al., 2010), to identify network alterations that reliably distinguish between the presence and absence of consciousness. Statistical significance between each pair of conditions was determined by the NBS with an intensity‐based F‐threshold of 10

**FIGURE 6:**
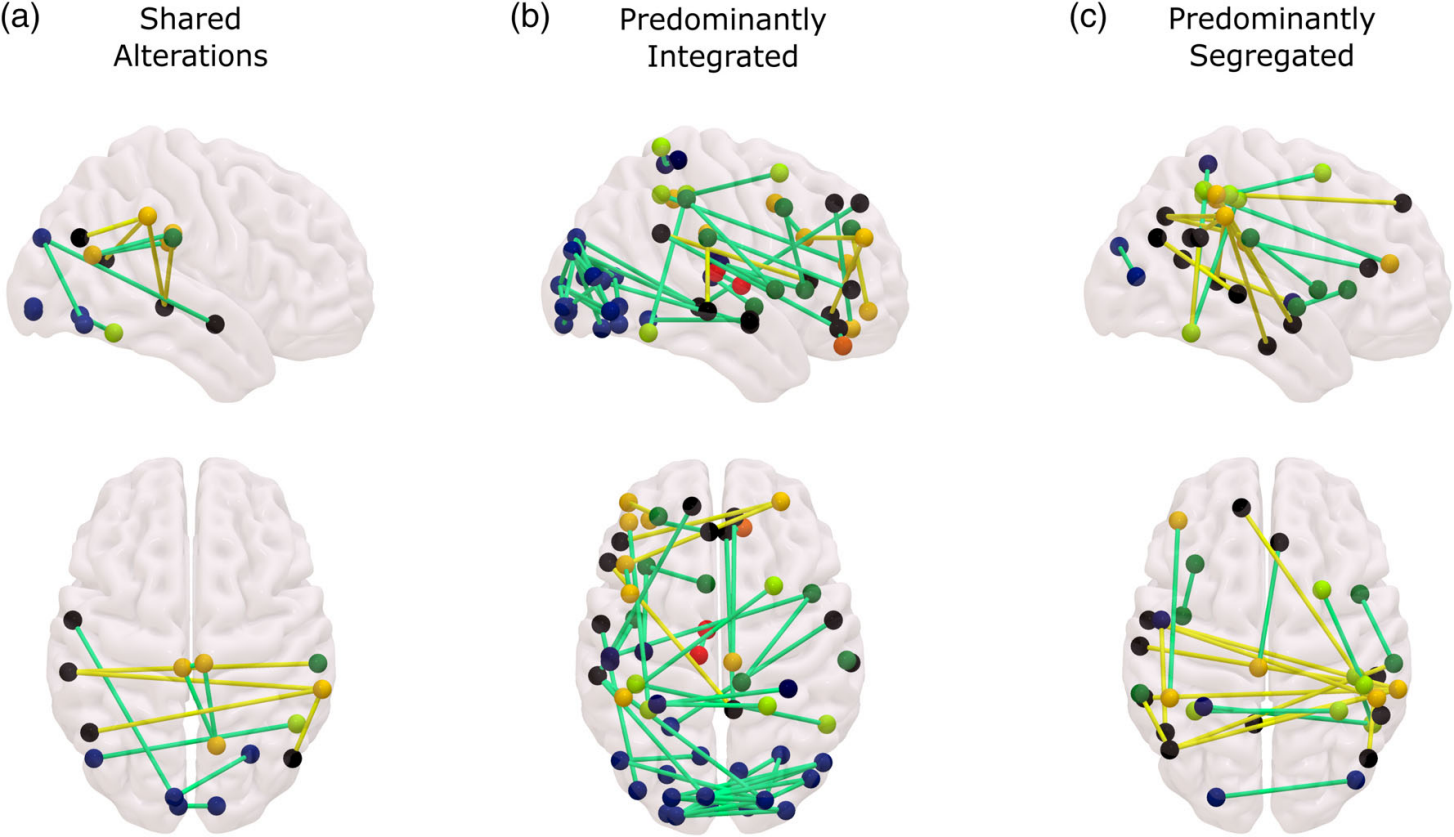
Consciousness‐specific dynamic reorganisation of functional connectivity induced by sevoflurane. Separately for the predominantly integrated and segregated sub‐states, the common changes identified by the network‐based statistic (NBS) between all conscious and all unconscious conditions (Figures 4 and 5) are identified. The results are then compared between the two dynamic sub‐states, to identify alterations that are insensitive to brain network dynamics, being shared by both sub‐states (a), versus alterations that are consistently observed only in the integrated sub‐state (b) or only in the segregated sub‐state (c). Yellow edges: conscious > unconscious. Green edges: unconscious > conscious. Black nodes: default mode network. Light blue: visual network. Dark blue: sensorimotor network. Dark green: salience/ventral attention network. Light green: dorsal attention network. Yellow: fronto‐parietal network. Regions of interest (ROIs) were assigned to resting‐state network by Schaefer et al. (2018) based on the definitions of Yeo et al. (2011). Statistical significance between each pair of conditions was determined by the network‐based statistic (NBS) with an intensity‐based F‐threshold of 10. The same results were obtained using an extent‐based threshold instead (Figure S3)

Thus, separately for the predominantly integrated and segregated sub‐states, we used the NBS (Zalesky et al., 2010) to compare each level of anaesthesia (BS, 3% vol and 2% vol) with the awake condition, and also with post‐anaesthetic recovery. The NBS revealed that for both integrated and segregated sub‐states, wakefulness and recovery were both significantly different from BS, 3% volume and 2% volume of sevoflurane in terms of network‐level connectivity (Figures 4 and 5). Moreover, in addition to alterations observed in both dynamic sub‐states (Figure 6a), a number of specific connectivity alterations were identified, which were exclusively observed during either the predominantly integrated or the predominantly segregated sub‐states of dFC—demonstrating the importance of taking brain network dynamics into account (Figure 6b,c).

Following Luppi et al. (2019), the predominantly integrated sub‐state displayed comparatively more extensive reorganisation of brain connectivity, than the segregated sub‐state. Connectivity changes in the integrated sub‐state mostly consisted of reductions, especially between visual regions (dark blue) but also extensively involving regions of the default mode network (DMN; black nodes, Figure 6b), and a disconnection between anterior and posterior portions of the left thalamus; however, a small number of increases in connectivity were also observed, primarily involving anterior regions of the fronto‐parietal control network (FPN; yellow nodes) (Figure 6b).

In contrast, the majority of alterations that were exclusively observed during the predominantly segregated sub‐state consisted of interhemispheric increased connectivity (reduced anticorrelations) between the default mode and FPN or dorsal attention networks (DAN; dark green nodes) (Figure 6c). The DMN is prominently involved in supporting human consciousness (Bodien, Threlkeld, & Edlow, 2019; Campbell et al., 2020; Di Perri et al., 2016; Luppi et al., 2019; Luppi, Mediano, Rosas, Allanson, et al., 2020; Threlkeld et al., 2018), and a perturbation of its characteristic alternation with the FPN/DAN has been repeatedly observed during unconsciousness due to DOC, propofol, as well as sevoflurane (Bonhomme et al., 2016; Boveroux, Vanhaudenhuyse, & Phillips, 2010; Demertzi et al., 2015; Deshpande et al., 2010; Di Perri et al., 2018; Huang et al., 2020; Kafashan et al., 2016; Luppi et al., 2019; Palanca et al., 2015, 2017).

### 3.3 Diminished functional network small‐worldness under sevoflurane

In addition to reorganisation of individual connections between brain regions, previous evidence indicates that loss of consciousness also induces changes in the global topology of functional brain networks. Specifically, a measure of optimal information transfer known as the network's small‐world character is compromised in a dynamic fashion during loss of consciousness induced by propofol anaesthesia (Barttfeld et al., 2015) or severe brain injury (Luppi et al., 2019). Moreover, such impairment was not uniform over time, but rather informed by brain dynamics of integration and segregation (Luppi et al., 2019). Therefore, here we analysed the small‐world propensity of brain networks across levels of sevoflurane anaesthesia, separately for the predominantly integrated and segregated dynamic sub‐states.

**FIGURE 7:**
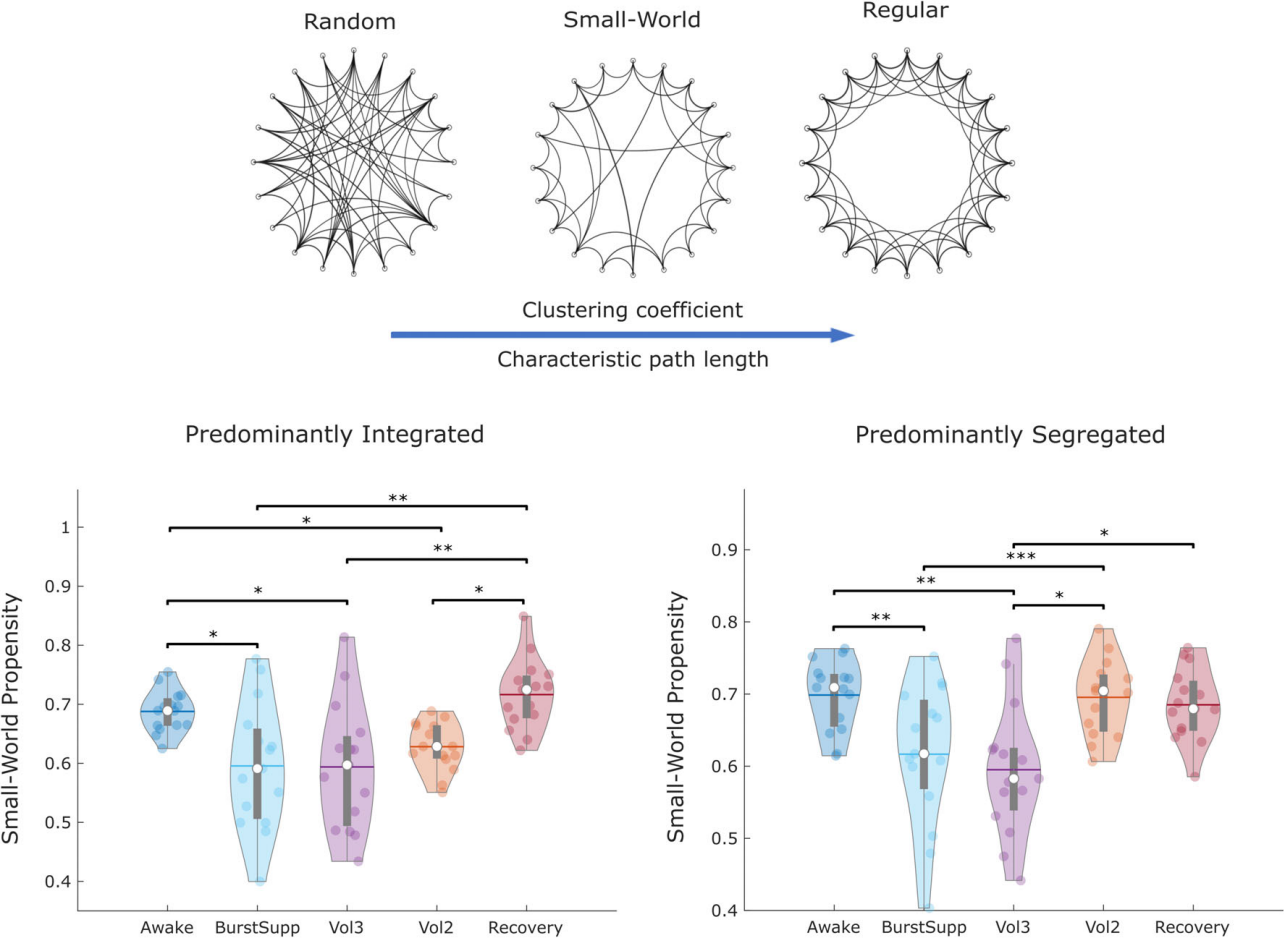
Reduced small‐world propensity of dynamic brain networks under sevoflurane. Violin plots represent the distribution of small‐world propensity for the predominantly integrated sub‐state, and the predominantly segregated dynamic sub‐state, across levels of sevoflurane. White circle, median; horizontal centre line, mean; box limits, upper and lower quartiles; whiskers, 1.5× interquartile range. *p < .05; **p < .01; ***p < .001, after Bonferroni correction

Analysis of variance indicated a significant effect of sevoflurane concentration on small‐world propensity of brain networks, both for the predominantly integrated (*F*[4,69] = 7.81, *p* < .001) and predominantly segregated sub‐states (*F*[4,69] = 11.02, *p* < .001). Post‐hoc Bonferroni‐corrected pairwise tests revealed that indeed, small‐world propensity of brain networks was decreased during sevoflurane‐induced LOR, reaching its lowest level at 3% sevoflurane concentration, and it returned to baseline levels after awakening (Figure 7 and Tables S4 and S5). However, small‐world propensity appeared to follow different recovery trajectories when the dynamics of brain integration and segregation were taken into account. For the predominantly integrated sub‐state, small‐world propensity decreased sharply at BS, but then returned gradually to baseline levels upon awakening; conversely, for the predominantly segregated sub‐state the return to baseline levels occurred earlier, when sevoflurane concentration was reduced from 3 to 2% volume, even though at 2% volume the participants were still deeply unresponsive.

For both dynamic sub‐states, the overall reduction in small‐world propensity under sevoflurane resulted from the balance of two opposing trends (Figure S4): the deviation of characteristic path length from a corresponding random network (Δ_*L*_) decreased during anaesthesia, which would lead to increased small‐world propensity; however, this effect was more than compensated by an increase in the deviation of the network's clustering coefficient from that of a regular network (Δ_*C*_) — resulting in overall increased small‐world propensity of brain networks, due to substantially compromised capacity for local processing (Tables S6–S9).

### 3.4 Diminished complexity of functional connections during sevoflurane anaesthesia

Finally, recent research has converged in indicating that spatial and temporal complexity of the brain are reduced during propofol‐induced general anaesthesia (Varley et al., 2020), and also in patients with DOC (Luppi et al., 2019). Complexity is increasingly recognised as a fundamental property for the brain's ability to support a wide repertoire of conscious states (Carhart‐Harris, 2018; Carhart‐Harris et al., 2014; Tononi et al., 1994; Wenzel et al., 2019). In particular, it was shown that a measure of brain complexity, the diversity (entropy) of connectivity between brain regions, is not uniform in time, but rather it is particularly disrupted by loss of consciousness during predominantly integrated states of brain dynamics (Luppi et al., 2019). Therefore, we sought to determine whether the complexity of functional connectivity patterns was reduced during sevoflurane anaesthesia, and whether this disruption exhibited differences between the predominantly integrated and segregated sub‐states of brain dynamics, as previously observed with loss of consciousness induced by propofol or severe brain injury. Therefore, we examined the Shannon entropy of brain connectivity patterns (Saenger et al., 2018) across levels of sevoflurane, separately for the predominantly integrated and segregated dynamic sub‐states.

**FIGURE 8:**
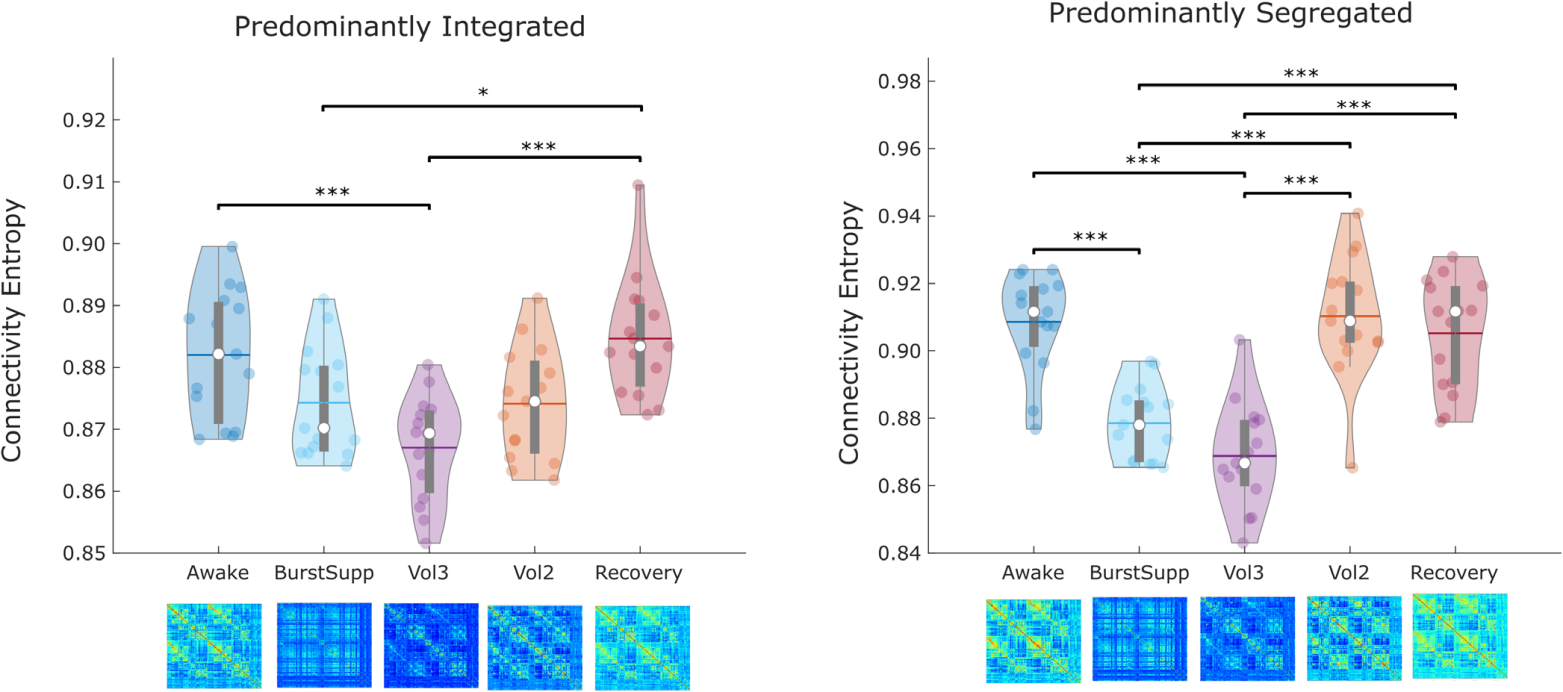
Reduced entropy of functional connections between brain regions under the effects of sevoflurane. Violin plots represent the distribution of connectivity entropy across the whole brain for the predominantly integrated sub‐state, and the predominantly segregated dynamic sub‐state, across levels of sevoflurane. White circle, median; horizontal centre line, mean; box limits, upper and lower quartiles; whiskers, 1.5× interquartile range. *p < .05; **p < .01; ***p < .001, after Bonferroni correction

Analysis of variance indicated a significant effect of anaesthetic depth on the entropy of connection patterns between regions, for both the predominantly integrated (*F*[4,69] = 9.60, *p* < .001) and predominantly segregated sub‐states (*F*[4,69] = 30.19, *p* < .001). Subsequent Bonferroni‐corrected pairwise tests revealed that, as expected, the entropy of whole‐brain connectivity patterns was significantly reduced at high concentrations of sevoflurane, and returned to baseline upon awakening (Tables S10 and S11). Similarly to small‐world propensity, the lowest values of connectivity entropy were observed at 3% volume of sevoflurane (Figure 8). However, the patterns of changes appeared to differ between predominantly integrated and segregated dynamic sub‐states. For the integrated sub‐state, decrease in connectivity entropy was not immediate: no significant difference was observed between wakefulness and BS; likewise, recovery was also gradual (Figure 8). In contrast, the segregated sub‐state exhibited a sharp drop in connectivity entropy between wakefulness and BS, as well as a subsequent sharp increase back to baseline levels when sevoflurane concentration was reduced from 3 to 2% volume, despite the fact that participants were still anaesthetised—analogously to small‐world propensity (Figure 8).

### 3.5 Validation of results

The present results were obtained with nodes defined by the augmented Schaefer‐232 atlas, which was recently found to produce the most topologically representative brain networks among a set of 9 anatomical and functional parcellations of different granularity (Luppi & Stamatakis, 2020). Nevertheless, since our results are strongly dependent on network characterisation, we further demonstrated their robustness by replicating them using an alternative way of defining brain network nodes. Specifically, we employed the Brainnetome atlas (Fan et al., 2016), which comprises 246 cortical and subcortical ROIs obtained from combined anatomical and functional connectivity (Figures S5–S9). Of note, the disconnection between anterior and posterior portions of the left thalamus (rostral and caudal left temporal thalamus) was shared by the integrated and segregated sub‐states, instead of being specific to the predominantly integrated sub‐state. Several regions of the limbic subnetwork of Yeo et al. (2011) also showed reduced connectivity, especially in the integrated sub‐state.

For the Schaefer‐232 atlas, we also replicated our results without excluding the subject with high number of scrubbed volumes, and without including the number of scrubbed volumes as a covariate in our statistical analyses, relying instead on non‐parametric tests (permutation‐based) to account for any outliers (Figures S10–S14). The main difference was that a substantially larger number of connectivity changes were evident across both sub‐states of dFC, at least some of which may be related to the effects of scrubbing. Finally, we also show that our results are replicated using shorter (18 TRs ~33 s) and longer (27 TRs ~50s) window lengths (Figures S15–S17).

## 4 DISCUSSION

Here we combined fMRI dFC and graph theory to investigate two fundamental properties of both the human mind and brain—integration and segregation—and how they are affected by general anaesthesia induced by the inhalational anaesthetic, sevoflurane, in a re‐analysis of previously published data (Ranft et al., 2016). Specifically, we hypothesised that if the effects on brain integration and segregation observed in propofol anaesthesia and DOC patients are involved in supporting consciousness, then (a) they should also be observed with sevoflurane; and (b) they should be reversed upon recovery.

**FIGURE 9:**
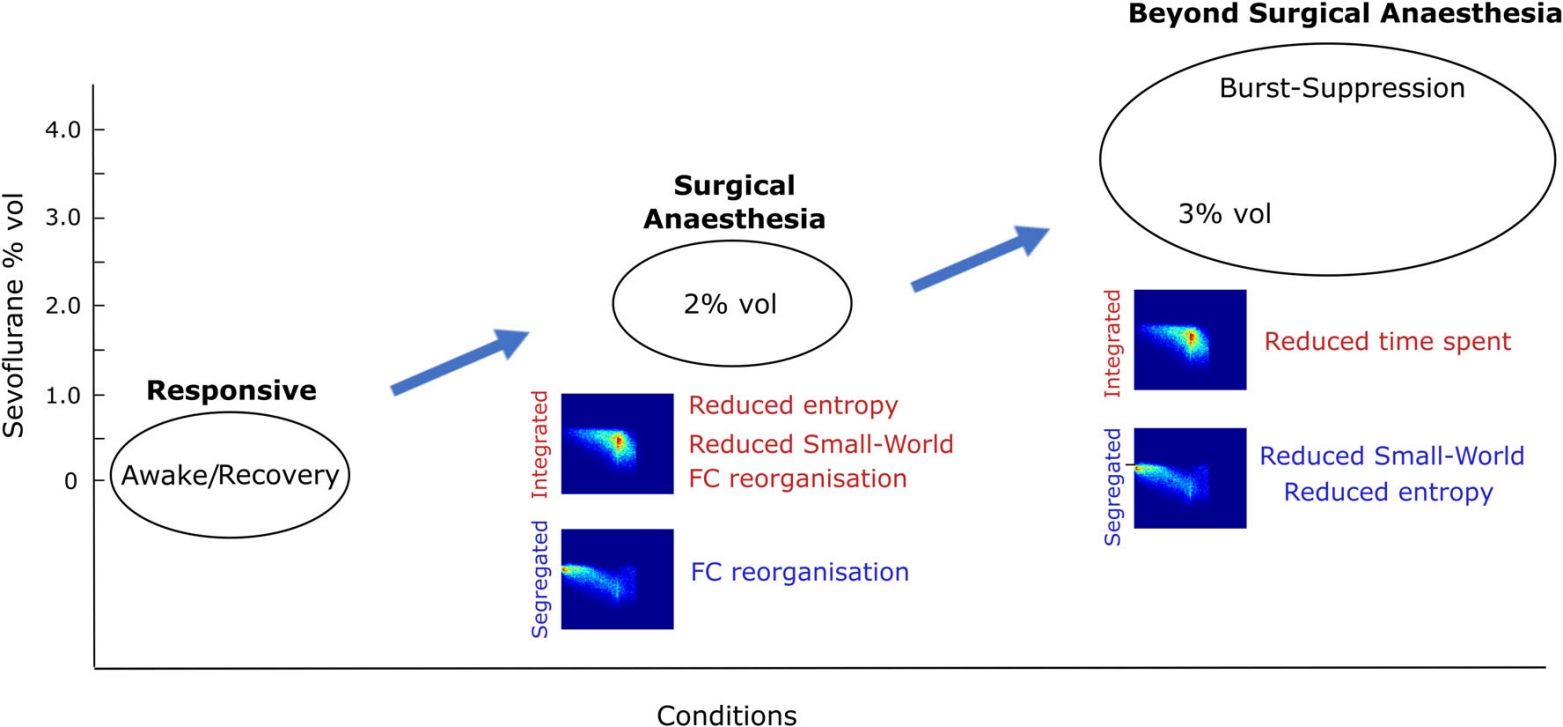
Summary of dynamic network changes across levels of sevoflurane anaesthesia observed in the present study. (i) From responsiveness (awake or recovery) to surgical levels of anaesthesia (2% vol) participants become unresponsiveness (and presumably unconscious). The integrated sub‐state shows the most prominent changes, with reduced entropy and small‐world propensity (SW) as well as functional connectivity reorganisation; functional connectivity changes are also observed in the segregated sub‐state. (ii) Beyond surgical levels of anaesthesia (3% vol and burst‐suppression), there is a reduction of the time spent in the integrated sub‐state; additionally, the segregated sub‐state exhibits reductions in entropy and small‐world propensity (note that this second step only mentions additional changes, which were not already present at the previous step)

To this end, we leveraged a well‐established method known as “cartographic profile” to obtain dynamic sub‐states of brain integration and segregation, known to play different roles in human cognition and consciousness (Fukushima et al., 2018; Luppi et al., 2019, 2021; Shine et al., 2016). A summary of our key findings is presented in Figure 9.

Remarkably, our first observation was that at the deepest levels of sevoflurane (BS and 3% volume), the brain switched from spending most of the time in the predominantly integrated sub‐state of dFC, to spending most time in the segregated sub‐state instead. We note that in earlier work (Luppi et al., 2019), we did not observe differences in the proportion of time spent in the integrated sub‐state during unconsciousness. However, the propofol anaesthesia described in Luppi et al. (2019) (Ramsay level 5) was most similar to the 2% vol concentration in the current study (Ramsay scale level 6) (Ranft et al., 2016). Corroborating our earlier work, at 2% vol our participants were still deeply unresponsive, but the proportion of time spent in the integrated sub‐state returned to wake‐like levels. Thus, the present results are in line with our previous observation that loss of consciousness (identified in terms of LOR), induced by a dose of propofol comparable with 2% vol sevoflurane, does not alter the temporal balance of integrated and segregated states in the human brain. Consequently, we interpret the reduced time spent in the integrated sub‐state at higher sevoflurane concentrations as being related to the drug dosage, rather than the absence of consciousness (indicated by responsiveness) per se.

This observation may bear direct clinical relevance. A sevoflurane concentration of 2% vol is compatible with surgical anaesthesia in adults (Miller, Theodore, & Widrich, 2020), and depth of anaesthesia beyond surgical requirements is associated with delayed recovery, and risk of postoperative delirium and cognitive dysfunction (Radtke et al., 2013). Recent work indicates that patients with more EEG suppression are more likely to experience postoperative delirium (Fritz et al., 2016), as are patients who experience EEG suppression at lower concentrations of volatile anaesthetic (Fritz, Maybrier, & Avidan, 2018). On the other hand, EEG suppression was not associated with long‐term changes in cognitive ability in patients or healthy volunteers (Fritz et al., 2016; Shortal et al., 2019), and delirium itself appears to be better predicted by preoperative abnormal cognition than by EEG suppression (Fritz et al., 2020). Crucially, here we observed reduced time spent in the integrated sub‐state already before the BS stage. Thus, it is tempting to speculate that the disrupted temporal balance of brain integration and segregation observed at 3% concentration of sevoflurane and above may be related to deeper anaesthesia and subsequent delirium and cognitive deficits. Future work may directly investigate whether disrupted temporal balance of brain integration and segregation could represent a predictor or even part of the neuronal mechanism responsible for postoperative delirium and cognitive deficits, possibly even before the stage of BS is reached.

Supporting the notion that high doses of sevoflurane have additional effects beyond those exerted to induce LOR, at BS and 3% volume of sevoflurane we also observed reductions in the complexity (entropy) of functional connections and brain network small‐world propensity, for both the integrated and segregated dynamic sub‐states—whereas previously such results had only been reported for the integrated sub‐state (Luppi et al., 2019). Crucially, both small‐world propensity and entropy of the segregated sub‐state exhibited a significant increase back to baseline when sevoflurane concentration was reduced to 2%—whereas this was not observed for the integrated sub‐state: instead, the return to baseline levels upon recovery was more gradual.

Thus, although both dynamic sub‐states exhibit reduced small‐world character and reduced complexity of connectivity patterns at high levels of sevoflurane, at 2% concentration neither effect was observed for the segregated sub‐state—even though individuals were deeply unresponsive.

Therefore, supporting our earlier results (Luppi et al., 2019), the present findings suggest that reduced complexity and small‐worldness of the integrated sub‐state are associated with loss of responsiveness (and presumably consciousness), whereas changes in these properties observed during the segregated state appear to reflect drug dose. This is noteworthy because the integrated sub‐state is the one more closely related to alertness and cognitive performance (Shine et al., 2016).

Anticorrelations between the default mode and FPN/DAN are well‐known to characterise the awake human brain at rest, and a perturbation of their characteristic alternation—as we observed here—is a robust hallmark of unconsciousness shared by DOC and anaesthesia with propofol, sevoflurane and even the dissociative anaesthetic ketamine (Bonhomme et al., 2016; Boveroux et al., 2010; Demertzi et al., 2015; Di Perri et al., 2018; Golkowski et al., 2019; Huang et al., 2020; Luppi et al., 2019; Luppi, Mediano, Rosas, Allanson, et al., 2020; Ranft et al., 2016).

However, previous work examining the dynamics of reduced anticorrelations induced by propofol or severe brain injury reported that they occur primarily during the integrated sub‐state (Luppi et al., 2019). Instead, our results suggest that the segregated sub‐state is also involved in the reduced anticorrelations induced by sevoflurane. Although sevoflurane has more widespread molecular mechanisms of action than propofol (Palanca et al., 2017), an alternative explanation is that general anaesthesia was deeper in the present study than in our previous work (Luppi et al., 2019), and the present findings indicate that at sufficiently high sevoflurane concentrations, effects that were previously observed only during the integrated sub‐state can extend to the segregated sub‐state. This hypothesis may be further investigated in studies employing lower doses of sevoflurane, to determine at which point the reduction in anticorrelations spreads from the integrated to the segregated sub‐states of dFC.

Unlike previous studies (Golkowski et al., 2017; Luppi et al., 2019), we did not observe reconfigurations of thalamocortical connectivity; however, we did observe a decrease in connectivity between anterior and posterior portions of the left thalamus, which was consistently reversed when responsiveness was restored—indicating that it was specifically associated with LOR. Intriguingly, the thalamocortical disconnection of Luppi et al. (2019) was also left‐lateralised. These results suggest that when brain dynamics are considered, thalamocortical disconnection may be associated with loss but not recovery of responsiveness (and presumably consciousness). Conversely, anterior–posterior connectivity within the left thalamus itself (which [Luppi et al., 2019] could not examine due to their use of a coarser‐grained parcellation) seems to be more specific, being reduced upon LOR and restored upon recovery.

Our results are complementary to the dynamic analysis of Golkowski et al. (2019). Using a variable number of dynamic sub‐states, these researchers observed that anaesthesia with propofol or sevoflurane reduces the brain's dynamic repertoire. Conversely, our results using a fixed number of dynamic sub‐states based on network integration and segregation, reveal that the individual sub‐states and their network properties are themselves altered during LOR, even before their relative prevalence is impacted. In particular, we were able to further establish the relative sensitivity of each dynamic sub‐state to pharmacological perturbations, and identify which aspects are related to LOR rather than the drug's other effects on the brain—especially evident at higher concentrations.

The pattern of connectivity entropy changes, being most profound at 3% concentration of sevoflurane, is consistent with previous results obtained with permutation entropy in the same dataset (Ranft et al., 2016), even though those results pertained to entropy of EEG temporal signals rather than fMRI connectivity patterns. This robustness across domains mirrors recent evidence that propofol anaesthesia produces similar reductions of brain complexity, whether measured in the spatial or temporal domains (Varley, Luppi, et al., 2020).

The present findings further complement those of Luppi et al. (2019) in several respects. First, we show that their key findings pertaining to reduced complexity and small‐worldness of the integrated sub‐state during LOR, can be further generalised to general anaesthesia induced by a different avenue: sevoflurane inhalation. Second, Luppi and colleagues determined consciousness‐specificity in terms of similarities between acute propofol administration and chronic DOC: that is, they identified perturbations that accompanied unconsciousness (indicated by unresponsiveness) irrespective of its cause, across different individuals. However, their study did not include a comparison with post‐anaesthetic recovery. By showing that these putative consciousness‐specific alterations are reversed upon awakening, here we demonstrate that they specifically track consciousness also within the same individual. Thus, our results extend the generalisability of earlier findings, while also strengthening the case for their role in supporting human consciousness.

### 4.1 Limitations

This study has a number of limitations that should be taken into account. First, sevoflurane anaesthesia started at higher concentrations (BS), followed by reduced concentrations (3 and 2% volume). Since sevoflurane is known to induce a dose‐dependent increase of cerebral blood flow, despite its reduction in cerebral metabolism (Palanca et al., 2017), our design introduces the possibility that findings at lower anaesthetic concentrations may reflect insufficient recovery from the higher doses. However, data were acquired after steady‐state equilibrium had been reached. Additionally, our results pertaining to altered temporal balance between dynamic sub‐states were reversed when sevoflurane concentration dropped to 2% vol, and both small‐worldness and complexity of connectivity patterns exhibited significant differences between 2% vol and deeper levels. Finally, in addition to noting that motion, cardiac, physiological and respiratory artifacts are accounted for in our denoising procedure (Section 2), we also note that previous work using the same dataset reported consistent findings across fMRI and EEG, even though EEG is independent of the BOLD signal, and therefore unaffected by sevoflurane's effects on the coupling between cerebral metabolism and cerebral blood flow (Ranft et al., 2016).

We also acknowledge that although we used loss of behavioural responsiveness (LOR) as a proxy for unconsciousness, the two are theoretically distinct and can be dissociated in practice (Huang et al., 2018; Leslie et al., 2009; Monti et al., 2010; Ní Mhuircheartaigh, Warnaby, Rogers, Jbabdi, & Tracey, 2013; Owen et al., 2006). Nevertheless, this concern is mitigated in the present study by the fact that our analysis included very deep levels of anaesthesia, with the lowest (2% vol) being already equivalent to surgical levels (Katoh & Ikeda, 1987; Miller et al., 2020), and the deepest level inducing EEG burst‐suppression (Ranft et al., 2016), making the presence of consciousness despite unresponsiveness very unlikely. Nevertheless, the combination of our analyses with measures of consciousness that do not depend on responsiveness, such as the perturbational complexity index (Casali et al., 2013), could shed further light on their relevance for consciousness versus responsiveness.

Another limitation is that neither parcellation used included the cerebellum, whose role in anaesthetic‐induced loss of consciousness deserves further investigation. Finally, cartographic profile based on sliding‐windows is one among a rapidly expanding number of ways to investigate brain dynamics (Allen et al., 2014; Atasoy, Donnelly, & Pearson, 2016; Atasoy et al., 2017; Atasoy, Vohryzek, Deco, Carhart‐harris, & Kringelbach, 2018; Barttfeld et al., 2015; Cai, Wang, et al., 2020; Cai, Wei, et al., 2020; Cao et al., 2019; Deco et al., 2019; Demertzi et al., 2019; Eagleman, Chander, Reynolds, Ouellette, & MacIver, 2019; Eagleman et al., 2018; Fukushima et al., 2018; Hahn et al., 2020; Huang et al., 2020; Hutchison, Hutchison, Manning, Menon, & Everling, 2014; Hutchison et al., 2013; Lee et al., 2019; D. Li et al., 2019; Y. Li et al., 2020; Lord et al., 2019; Luppi, Vohryzek, Jakub, Kringelbach, et al., 2020; Lurie et al., 2020; Preti et al., 2017; Riehl et al., 2017; Shine et al., 2016, 2019; Standage et al., 2020; Uhrig et al., 2018; Varley, Denny, Sporns, & Patania, 2020; Vlisides et al., 2019; Vohryzek, Deco, Cessac, Kringelbach, & Cabral, 2020; Zamani Esfahlani et al., 2020; Y. Zhang et al., 2019, 2020). Each approach inevitably comes with both strengths and limitations, although converging evidence is already beginning to emerge across different methods.

## 5 CONCLUSION

Overall, we demonstrate that general anaesthesia induced by 2% vol sevoflurane is accompanied by reorganised functional connectivity and network properties within dynamic sub‐states, whereas higher doses also compromise the temporal balance of integration and segregation in the human brain. Taken together, our results demonstrate that brain network complexity and information capacity during dynamic states of high integration are hallmarks of intact human consciousness, being compromised during general anaesthesia, and restored upon recovery.

## CONFLICT OF INTEREST

The authors declare no competing financial interests.

## ETHICS STATEMENT

The ethics committee of the medical school of the Technische Universität München (München, Germany) approved the current study, which was conducted in accordance with the Declaration of Helsinki. Written informed consent was obtained from volunteers at least 48 hr before the study session.

## Supporting information


**Appendix** **S1:** Supplementary Information

## Data Availability

Raw data available from Dr. Golkowski: golkowski@lrz.tum.de. Code for the “cartographic profile” is freely available online (https://github.com/macshine/integration/). Code for the computation of small‐world propensity is freely available online (http://www.seas.upenn.edu/~dsb/). The Brain Connectivity Toolbox code used for graph‐theoretical analyses is freely available online (https://sites.google.com/site/bctnet/). The CONN toolbox is freely available online (http://www.nitrc.org/projects/conn). The Network Based Statistic is freely available online (https://www.nitrc.org/projects/nbs/).

## References

[hbm25405-bib-0001] Allen, E. A. , Damaraju, E. , Plis, S. M. , Erhardt, E. B. , Eichele, T. , & Calhoun, V. D. (2014). Tracking whole‐brain connectivity dynamics in the resting state. Cerebral Cortex, 24(3), 663–676. 10.1093/cercor/bhs352 23146964 PMC3920766

[hbm25405-bib-0002] Andellini, M. , Cannatà, V. , Gazzellini, S. , Bernardi, B. , & Napolitano, A. (2015). Test‐retest reliability of graph metrics of resting state MRI functional brain networks: A review. Journal of Neuroscience Methods, 253, 183–192. 10.1016/j.jneumeth.2015.05.020 26072249

[hbm25405-bib-0003] Atasoy, S. , Donnelly, I. , & Pearson, J. (2016). Human brain networks function in connectome‐specific harmonic waves. Nature Communications, 7, 1–10. 10.1038/ncomms10340 PMC4735826 26792267

[hbm25405-bib-0004] Atasoy, S. , Roseman, L. , Kaelen, M. , Kringelbach, M. L. , Deco, G. , & Carhart‐Harris, R. L. (2017). Connectome‐harmonic decomposition of human brain activity reveals dynamical repertoire re‐organization under LSD. Scientific Reports, 7(1), 1–18. 10.1038/s41598-017-17546-0 29247209 PMC5732294

[hbm25405-bib-0005] Atasoy, S. , Vohryzek, J. , Deco, G. , Carhart‐harris, R. L. , & Kringelbach, M. L. (2018). Common neural signatures of psychedelics: Frequency‐specific energy changes and repertoire expansion revealed using connectome‐harmonic decomposition. Progress in Brain Research, 242, 277–293. 10.1016/bs.pbr.2018.08.009 30471684

[hbm25405-bib-0006] Balduzzi, D. , & Tononi, G. (2008). Integrated information in discrete dynamical systems: Motivation and theoretical framework. PLoS Computational Biology, 4(6), e1000091. 10.1371/journal.pcbi.1000091 18551165 PMC2386970

[hbm25405-bib-0007] Barttfeld, P. , Uhrig, L. , Sitt, J. D. , Sigman, M. , Jarraya, B. , & Dehaene, S. (2015). Signature of consciousness in the dynamics of resting‐state brain activity. Proceedings of the National Academy of Sciences of the United States of America, 112(3), 887–892. 10.1073/pnas.1418031112 25561541 PMC4311826

[hbm25405-bib-0008] Bassett, D. S. , & Bullmore, E. T. (2017). Small‐world brain networks revisited. The Neuroscientist, 23, 499–516. 10.1177/1073858416667720 27655008 PMC5603984

[hbm25405-bib-0009] Behzadi, Y. , Restom, K. , Liau, J. , & Liu, T. T. (2007). A component based noise correction method (CompCor) for BOLD and perfusion based fMRI. NeuroImage, 37, 90–101. 17560126 10.1016/j.neuroimage.2007.04.042 PMC2214855

[hbm25405-bib-0010] Blain‐Moraes, S. , Tarnal, V. , Vanini, G. , Bel‐Behar, T. , Janke, E. , Picton, P. , … Mashour, G. A. (2017). Network efficiency and posterior alpha patterns are markers of recovery from general anesthesia: A high‐density electroencephalography study in healthy volunteers. Frontiers in Human Neuroscience, 11, 1–8. 10.3389/fnhum.2017.00328 28701933 PMC5487412

[hbm25405-bib-0011] Blondel, V. D. , Guillaume, J.‐L. , Lambiotte, R. , & Lefebvre, E. (2008). Fast unfolding of communities in large networks. Journal of Statistical Mechanics: Theory and Experiment, 2008(10), P10008. 10.1088/1742-5468/2008/10/P10008

[hbm25405-bib-0012] Bodien, Y. G. , Threlkeld, Z. D. , & Edlow, B. L. (2019, October 1). Default mode network dynamics in covert consciousness. Cortex, 119, 571–574. 10.1016/j.cortex.2019.01.014 30791975 PMC6527357

[hbm25405-bib-0013] Bonhomme, V. , Vanhaudenhuyse, A. , Demertzi, A. , Bruno, M.‐A. , Jaquet, O. , Bahri, M. A. , … Laureys, S. (2016). Resting‐state network‐specific breakdown of functional connectivity during ketamine alteration of consciousness in volunteers. Anesthesiology, 125(5), 873–888. 10.1097/ALN.0000000000001275 27496657

[hbm25405-bib-0014] Boveroux, P. , Vanhaudenhuyse, A. , & Phillips, C. (2010). Breakdown of within‐ and between‐network resting state during propofol‐induced loss of consciousness. Anesthesiology, 113(5), 1038–1053. 20885292 10.1097/ALN.0b013e3181f697f5

[hbm25405-bib-0015] Braun, U. , Plichta, M. M. , Esslinger, C. , Sauer, C. , Haddad, L. , Grimm, O. , … Meyer‐Lindenberg, A. (2012). Test‐retest reliability of resting‐state connectivity network characteristics using fMRI and graph theoretical measures. NeuroImage, 59(2), 1404–1412. 10.1016/j.neuroimage.2011.08.044 21888983

[hbm25405-bib-0016] Buckner, R. L. , & DiNicola, L. M. (2019). The brain's default network: Updated anatomy, physiology and evolving insights. Nature Reviews Neuroscience, 20, 593–608. 10.1038/s41583-019-0212-7 31492945

[hbm25405-bib-0017] Cai, L. , Wang, J. , Guo, Y. , Lu, M. , Dong, Y. , & Wei, X. (2020). Altered inter‐frequency dynamics of brain networks in disorder of consciousness. Journal of Neural Engineering, 17(3), 1–13. 10.1088/1741-2552/ab8b2c 32311694

[hbm25405-bib-0018] Cai, L. , Wei, X. , Wang, J. , Yi, G. , Lu, M. , & Dong, Y. (2020). Characterization of network switching in disorder of consciousness at multiple time scales. Journal of Neural Engineering, 17(2), 026024. 10.1088/1741-2552/ab79f5 32097898

[hbm25405-bib-0019] Campbell, J. M. , Huang, Z. , Zhang, J. , Wu, X. , Qin, P. , Northoff, G. , … Hudetz, A. G. (2020). Pharmacologically informed machine learning approach for identifying pathological states of unconsciousness via resting‐state fMRI. NeuroImage, 206, 116316. 10.1016/j.neuroimage.2019.116316 31672663 PMC6981054

[hbm25405-bib-0020] Cao, B. , Chen, Y. , Yu, R. , Chen, L. , Chen, P. , Weng, Y. , … Huang, R. (2019). Abnormal dynamic properties of functional connectivity in disorders of consciousness. NeuroImage: Clinical, 24, 102071. 10.1016/j.nicl.2019.102071 31795053 PMC6881656

[hbm25405-bib-0021] Carhart‐Harris, R. L. (2018). The entropic brain—Revisited. Neuropharmacology, 142, 167–178. 10.1016/j.neuropharm.2018.03.010 29548884

[hbm25405-bib-0022] Carhart‐Harris, R. L. , Leech, R. , Hellyer, P. J. , Shanahan, M. , Feilding, A. , Tagliazucchi, E. , … Nutt, D. (2014). The entropic brain: A theory of conscious states informed by neuroimaging research with psychedelic drugs. Frontiers in Human Neuroscience, 8(February), 20. 10.3389/fnhum.2014.00020 24550805 PMC3909994

[hbm25405-bib-0023] Carhart‐Harris, R. L. , Muthukumaraswamy, S. , Roseman, L. , Kaelen, M. , Droog, W. , Murphy, K. , … Nutt, D. J. (2016). Neural correlates of the LSD experience revealed by multimodal neuroimaging. Proceedings of the National Academy of Sciences of the United States of America, 113(17), 201518377. 10.1073/pnas.1518377113 PMC4855588 27071089

[hbm25405-bib-0024] Casali, A. G. , Gosseries, O. , Rosanova, M. , Boly, M. , Sarasso, S. , Casali, K. R. , … Massimini, M. (2013). A theoretically based index of consciousness independent of sensory processing and behavior. Science Translational Medicine, 5, 1–10. 10.1017/CBO9781107415324.004 23946194

[hbm25405-bib-0025] Deco, G. , Cruzat, J. , Cabral, J. , Tagliazucchi, E. , Laufs, H. , Logothetis, N. K. , & Kringelbach, M. L. (2019). Awakening: Predicting external stimulation to force transitions between different brain states. Proceedings of the National Academy of Sciences of the United States of America, 116, 201905534. 10.1073/pnas.1905534116 PMC6731634 31427539

[hbm25405-bib-0026] Deco, G. , Tononi, G. , Boly, M. , & Kringelbach, M. L. (2015). Rethinking segregation and integration: Contributions of whole‐brain modelling. Nature Reviews Neuroscience, 16, 430–439. 10.1038/nrn3963 26081790

[hbm25405-bib-0027] Dehaene, S. , & Changeux, J.‐P. (2011). Experimental and theoretical approaches to conscious processing. Neuron, 70, 200–227. 10.1016/j.neuron.2011.03.018 21521609

[hbm25405-bib-0028] Dehaene, S. , Changeux, J. P. , & Naccache, L. (2011). The global neuronal workspace model of conscious access: From neuronal architectures to clinical applications. Research and Perspectives in Neurosciences, 18, 55–84. 10.1007/978-3-642-18015-6_4

[hbm25405-bib-0029] Demertzi, A. , Antonopoulos, G. , Heine, L. , Voss, H. U. , Crone, J. S. , De Los Angeles, C. , … Laureys, S. (2015). Intrinsic functional connectivity differentiates minimally conscious from unresponsive patients. Brain, 138(9), 2619–2631. 10.1093/brain/awv169 26117367

[hbm25405-bib-0030] Demertzi, A. , Martial, C. , Demertzi, A. , Tagliazucchi, E. , Dehaene, S. , Deco, G. , … Sitt, J. D. (2019). Human consciousness is supported by dynamic complex patterns of brain signal coordination. Science Advances, 5(February), 1–12. 10.1126/sciadv.aat7603 PMC6365115 30775433

[hbm25405-bib-0031] Deshpande, G. , Kerssens, C. , Sebel, P. S. , & Hu, X. (2010). Altered local coherence in the default mode network due to sevoflurane anesthesia. Brain Research, 1318, 110–121. 10.1016/j.brainres.2009.12.075 20059988 PMC2845285

[hbm25405-bib-0032] Di Perri, C. , Amico, E. , Heine, L. , Annen, J. , Martial, C. , Larroque, S. K. , … Laureys, S. (2018). Multifaceted brain networks reconfiguration in disorders of consciousness uncovered by co‐activation patterns. Human Brain Mapping, 39(1), 89–103. 10.1002/hbm.23826 29024197 PMC6866397

[hbm25405-bib-0033] Di Perri, C. , Bahri, M. A. , Amico, E. , Thibaut, A. , Heine, L. , Antonopoulos, G. , … Laureys, S. (2016). Neural correlates of consciousness in patients who have emerged from a minimally conscious state: A cross‐sectional multimodal imaging study. The Lancet Neurology, 15(8), 830–842. 10.1016/S1474-4422(16)00111-3 27131917

[hbm25405-bib-0034] Eagleman, S. L. , Chander, D. , Reynolds, C. , Ouellette, N. T. , & MacIver, M. B. (2019). Nonlinear dynamics captures brain states at different levels of consciousness in patients anesthetized with propofol. PLoS One, 14(10), e0223921. 10.1371/journal.pone.0223921 31665174 PMC6821075

[hbm25405-bib-0035] Eagleman, S. L. , Vaughn, D. A. , Drover, D. R. , Drover, C. M. , Cohen, M. S. , Ouellette, N. T. , & Maciver, M. B. (2018). Do complexity measures of frontal EEG distinguish loss of consciousness in geriatric patients under anesthesia? Frontiers in Neuroscience, 12(SEP), 1–13. 10.3389/fnins.2018.00645 30294254 PMC6158339

[hbm25405-bib-0036] Fan, L. , Li, H. , Zhuo, J. , Zhang, Y. , Wang, J. , Chen, L. , … Jiang, T. (2016). The human brainnetome atlas: A new brain atlas based on connectional architecture. Cerebral Cortex, 26(8), 3508–3526. 10.1093/cercor/bhw157 27230218 PMC4961028

[hbm25405-bib-0037] Fox, M. D. , Snyder, A. Z. , Vincent, J. L. , Corbetta, M. , Van Essen, D. C. , & Raichle, M. E. (2005). The human brain is intrinsically organized into dynamic, anticorrelated functional networks. Proceedings of the National Academy of Sciences of the United States of America, 102(27), 9673–9678. 10.1073/pnas.0504136102 15976020 PMC1157105

[hbm25405-bib-0038] Fritz, B. A. , Maybrier, H. R. , & Avidan, M. S. (2018). Intraoperative electroencephalogram suppression at lower volatile anaesthetic concentrations predicts postoperative delirium occurring in the intensive care unit. British Journal of Anaesthesia, 121(1), 241–248. 10.1016/j.bja.2017.10.024 29935578 PMC6200110

[hbm25405-bib-0039] Fritz, B. A. , Kalarickal, P. L. , Maybrier, H. R. , Muench, M. R. , Dearth, D. , Chen, Y. , … Avidan, M. S. (2016). Intraoperative electroencephalogram suppression predicts postoperative delirium. Anesthesia and Analgesia, 122(1), 234–242. 10.1213/ANE.0000000000000989 26418126 PMC4684753

[hbm25405-bib-0040] Fritz, B. A. , King, C. R. , Ben Abdallah, A. , Lin, N. , Mickle, A. M. , Budelier, T. P. , … Tappenden, J. (2020). Preoperative cognitive abnormality, intraoperative electroencephalogram suppression, and postoperative delirium: A mediation analysis. Anesthesiology, 132(6), 1458–1468. 10.1097/ALN.0000000000003181 32032096 PMC7228853

[hbm25405-bib-0041] Fukushima, M. , Betzel, R. F. , He, Y. , van den Heuvel, M. P. , Zuo, X. N. , & Sporns, O. (2018). Structure–function relationships during segregated and integrated network states of human brain functional connectivity. Brain Structure and Function, 223(3), 1091–1106. 10.1007/s00429-017-1539-3 29090337 PMC5871577

[hbm25405-bib-0042] Golkowski, D. , Larroque, S. K. , Vanhaudenhuyse, A. , Plenevaux, A. , Boly, M. , Di Perri, C. , … Ilg, R. (2019). Changes in whole brain dynamics and connectivity patterns during sevoflurane‐ and propofol‐induced unconsciousness identified by functional magnetic resonance imaging. Anesthesiology, 130(6), 898–911. 10.1097/ALN.0000000000002704 31045899

[hbm25405-bib-0043] Golkowski, D. , Ranft, A. , Kiel, T. , Riedl, V. , Kohl, P. , Rohrer, G. , … Jordan, D. (2017). Coherence of BOLD signal and electrical activity in the human brain during deep sevoflurane anesthesia. Brain and Behavior, 7(7), 1–8. 10.1002/brb3.679 PMC5516594 28729926

[hbm25405-bib-0044] Hahn, G. , Zamora‐López, G. , Uhrig, L. , Tagliazucchi, E. , Laufs, H. , Mantini, D. , … Deco, G. (2020). Signature of consciousness in brain‐wide synchronization patterns of monkey and human fMRI signals. NeuroImage, 226, 117470. 10.1016/j.neuroimage.2020.117470 33137478

[hbm25405-bib-0045] Huang, Z. , Vlisides, P. E. , Tarnal, V. C. , Janke, E. L. , Keefe, K. M. , Collins, M. M. , … Hudetz, A. G. (2018). Brain imaging reveals covert consciousness during behavioral unresponsiveness induced by propofol. Scientific Reports, 8(1), 1–11. 10.1038/s41598-018-31436-z 30181567 PMC6123455

[hbm25405-bib-0046] Huang, Z. , Zhang, J. , Wu, J. , Mashour, G. A. , & Hudetz, A. G. (2020). Temporal circuit of macroscale dynamic brain activity supports human consciousness. Science Advances, 6(11), 87–98. 10.1126/sciadv.aaz0087 PMC7065875 32195349

[hbm25405-bib-0047] Huang, Z. , Zhang, J. , Wu, J. , Qin, P. , Wu, X. , Wang, Z. , … Northoff, G. (2016). Decoupled temporal variability and signal synchronization of spontaneous brain activity in loss of consciousness: An fMRI study in anesthesia. NeuroImage, 124, 693–703. 10.1016/j.neuroimage.2015.08.062 26343319

[hbm25405-bib-0048] Humphries, M. D. , & Gurney, K. (2008). Network “small‐world‐ness”: A quantitative method for determining canonical network equivalence. PLoS One, 3(4), e0002051. 10.1371/journal.pone.0002051 18446219 PMC2323569

[hbm25405-bib-0049] Hutchison, R. M. , Hutchison, M. , Manning, K. Y. , Menon, R. S. , & Everling, S. (2014). Isoflurane induces dose‐dependent alterations in the cortical connectivity profiles and dynamic properties of the brain's functional architecture. Human Brain Mapping, 35(12), 5754–5775. 10.1002/hbm.22583 25044934 PMC6869297

[hbm25405-bib-0050] Hutchison, R. M. , Womelsdorf, T. , Allen, E. A. , Bandettini, P. A. , Calhoun, V. D. , Corbetta, M. , … Chang, C. (2013). Dynamic functional connectivity: Promise, issues, and interpretations. NeuroImage, 80, 5–79. 10.1016/j.neuroimage.2013.05.079 PMC3807588 23707587

[hbm25405-bib-0051] Kafashan, M. , Ching, S. , & Palanca, B. J. A. (2016). Sevoflurane alters spatiotemporal functional connectivity motifs that link resting‐state networks during wakefulness. Frontiers in Neural Circuits, 10, 1–11. 10.3389/fncir.2016.00107 28082871 PMC5187351

[hbm25405-bib-0052] Katoh, T. , & Ikeda, K. (1987). The minimum alveolar concentration (MAC) of sevoflurane in humans. Anesthesiology, 66(3), 301–303. 10.1097/00000542-198703000-00006 3826687

[hbm25405-bib-0053] Lee, H. , Golkowski, D. , Jordan, D. , Berger, S. , Ilg, R. , Lee, J. , … Vlisides, P. E. (2019). Relationship of critical dynamics, functional connectivity, and states of consciousness in large‐scale human brain networks. NeuroImage, 188(May 2018), 228–238. 10.1016/j.neuroimage.2018.12.011 30529630

[hbm25405-bib-0054] Leslie, K. , Sleigh, J. , Paech, M. J. , Voss, L. , Lim, C. W. , & Sleigh, C. (2009). Dreaming and electroencephalographic changes during anesthesia maintained with propofol or desflurane. Anesthesiology, 111(3), 547–555. 10.1097/ALN.0b013e3181adf768 19672164

[hbm25405-bib-0055] Li, D. , Vlisides, P. E. , Kelz, M. B. , Avidan, M. S. , Mashour, G. A. , Blain‐Moraes, S. , … Palanca, B. J. A. (2019). Dynamic cortical connectivity during general anesthesia in healthy volunteers. Anesthesiology, 130(6), 870–884. 10.1097/ALN.0000000000002656 30946055

[hbm25405-bib-0056] Li, Y. , Shi, W. , Liu, Z. , Li, J. , Wang, Q. , Yan, X. , … Wang, G. (2020). Effective brain state estimation during propofol‐induced sedation using advanced EEG microstate spectral analysis. IEEE Journal of Biomedical and Health Informatics, 2194c, 1–1. 10.1109/jbhi.2020.3008052 32749987

[hbm25405-bib-0057] Lord, L. D. , Expert, P. , Atasoy, S. , Roseman, L. , Rapuano, K. , Lambiotte, R. , … Cabral, J. (2019). Dynamical exploration of the repertoire of brain networks at rest is modulated by psilocybin. NeuroImage, 199, 127–142. 10.1016/j.neuroimage.2019.05.060 31132450

[hbm25405-bib-0058] Luppi, A. I. , Carhart‐Harris, R. L. , Roseman, L. , Pappas, I. , Menon, D. K. , & Stamatakis, E. A. (2021). LSD alters dynamic integration and segregation in the human brain. NeuroImage, 227(December), 117653. 10.1016/j.neuroimage.2020.117653 33338615 PMC7896102

[hbm25405-bib-0059] Luppi, A. I. , Craig, M. M. , Pappas, I. , Finoia, P. , Williams, G. B. , Allanson, J. , … Stamatakis, E. A. (2019). Consciousness‐specific dynamic interactions of brain integration and functional diversity. Nature Communications, 10(1), 4616. 10.1038/s41467-019-12658-9 PMC6787094 31601811

[hbm25405-bib-0060] Luppi, A. I. , Mediano, P. A. , Rosas, F. E. , Allanson, J. , Carhart‐Harris, R. L. , Williams, G. B. , … Stamatakis, E. A. (2020). A synergistic workspace for human consciousness revealed by integrated information decomposition. *BioRxiv*, 2020.11.25.398081. 10.1101/2020.11.25.398081 PMC11257694 39022924

[hbm25405-bib-0061] Luppi, A. I. , Mediano, P. A. , Rosas, F. E. , Holland, N. , Fryer, T. D. , O'Brien, J. T. , … Stamatakis, E. A. (2020). A synergistic core for human brain evolution and cognition. *BioRxiv*, 2020.09.22.308981. 10.1101/2020.09.22.308981 PMC7614771 35618951

[hbm25405-bib-0062] Luppi, A. I. , & Stamatakis, E. A. (2020). Combining network topology and information theory to construct representative brain networks. Network Neuroscience, 5, 1–46. 10.1162/netn_a_00170 PMC7935031 33688608

[hbm25405-bib-0063] Luppi, A. I. , Vohryzek, J. , Kringelbach, M. L. , Mediano, P. A. , Craig, M. M. , Adapa, R. , … Stamatakis, E. A. (2020). Connectome harmonic decomposition of human brain dynamics reveals a landscape of consciousness. *BioRxiv* . 10.1101/2020.08.10.244459

[hbm25405-bib-0064] Lurie, D. J. , Kessler, D. , Bassett, D. S. , Betzel, R. F. , Breakspear, M. , Keilholz, S. D. , … Calhoun, V.D. (2020). On the nature of time‐varying functional connectivity in resting fMRI. Network Neuroscience, 4(1), 30–69. 10.1162/netn_a_00116 32043043 PMC7006871

[hbm25405-bib-0065] Lydon‐Staley, D. M. , Ciric, R. , Satterthwaite, T. D. , & Bassett, D. S. (2019). Evaluation of confound regression strategies for the mitigation of micromovement artifact in studies of dynamic resting‐state functional connectivity and multilayer network modularity. Network Neuroscience, 3(2), 427–454. 10.1162/netn_a_00071 30793090 PMC6370491

[hbm25405-bib-0066] Martuzzi, R. , Ramani, R. , Qiu, M. , Rajeevan, N. , & Constable, T. (2010). Functional connectivity and alterations in baseline brain state in humans. NeuroImage, 49(1), 823–834. 10.1016/j.neuroimage.2009.07.028.Functional 19631277 PMC2764802

[hbm25405-bib-0067] Martuzzi, R. , Ramani, R. , Qiu, M. , Shen, X. , Papademetris, X. , & Constable, R. T. (2011). A whole‐brain voxel based measure of intrinsic connectivity contrast reveals local changes in tissue connectivity with anesthetic without a priori assumptions on thresholds or regions of interest. NeuroImage, 58, 1044–1050. 10.1016/j.neuroimage.2011.06.075 21763437 PMC3183817

[hbm25405-bib-0068] Mashour, G. A. , Roelfsema, P. , Changeux, J. P. , & Dehaene, S. (2020). Conscious processing and the global neuronal workspace hypothesis. Neuron, 105, 776–798. 10.1016/j.neuron.2020.01.026 32135090 PMC8770991

[hbm25405-bib-0069] Miller, A. L. , Theodore, D. , & Widrich, J. (2020). Inhalational Anesthetic 9. Retrieved from https://www.ncbi.nlm.nih.gov/books/NBK554540/ 32119427

[hbm25405-bib-0070] Monti, M. M. , Vanhaudenhuyse, A. , Coleman, M. R. , Boly, M. , Pickard, J. D. , Tshibanda, L. , … Laureys, S. (2010). Willful modulation of brain activity in disorders of consciousness. New England Journal of Medicine, 362(7), 579–589. 10.1056/NEJMoa0905370 20130250

[hbm25405-bib-0071] Muldoon, S. F. , Bridgeford, E. W. , & Bassett, D. S. (2016). Small‐world propensity and weighted brain networks. Scientific Reports, 6, 1–13. 10.1038/srep22057 26912196 PMC4766852

[hbm25405-bib-0072] Ní Mhuircheartaigh, R. , Warnaby, C. , Rogers, R. , Jbabdi, S. , & Tracey, I. (2013). Slow‐wave activity saturation and thalamocortical isolation during propofol anesthesia in humans. Science Translational Medicine, 5(208), 208ra148. 10.1126/scitranslmed.3006007 24154602

[hbm25405-bib-0073] Nir, T. , Jacob, Y. , Huang, K. H. , Schwartz, A. E. , Brallier, J. W. , Ahn, H. , … Mincer, J. S. (2020). Resting‐state functional connectivity in early postanaesthesia recovery is characterised by globally reduced anticorrelations. British Journal of Anaesthesia, 125(4), 529–538. 10.1016/j.bja.2020.06.058 32800503 PMC7565909

[hbm25405-bib-0074] Northoff, G. , Wainio‐Theberge, S. , & Evers, K. (2020). Is temporo‐spatial dynamics the “common currency” of brain and mind? In Quest of “Spatiotemporal Neuroscience.”. Physics of Life Reviews, 33, 34–54. 10.1016/j.plrev.2019.05.002 31221604

[hbm25405-bib-0075] Owen, A. M. , Coleman, M. R. , Boly, M. , Davis, M. H. , Laureys, S. , & Pickard, J. D. (2006). Detecting awareness in the vegetative state. Science, 313(5792), 1402. 10.1126/science.1130197 16959998

[hbm25405-bib-0076] Palanca, B. J. A. , Avidan, M. S. , & Mashour, G. A. (2017). Human neural correlates of sevoflurane‐induced unconsciousness. BJA: British Journal of Anaesthesia, 119(4), 573–582. 10.1093/bja/aex244 29121298 PMC6172973

[hbm25405-bib-0077] Palanca, B. J. A. , Mitra, A. , Larson‐Prior, L. , Snyder, A. Z. , Avidan, M. S. , & Raichle, M. E. (2015). Resting‐state functional magnetic resonance imaging correlates of sevoflurane‐induced unconsciousness. Anesthesiology, 123(2), 346–356. 10.1097/ALN.0000000000000731 26057259 PMC4509973

[hbm25405-bib-0078] Papo, D. , Zanin, M. , Martínez, J. H. , & Buldú, J. M. (2016). Beware of the small‐world neuroscientist! Frontiers in Human Neuroscience, 10, 96. 10.3389/fnhum.2016.00096 27014027 PMC4781830

[hbm25405-bib-0079] Power, J. D. , Barnes, K. A. , Snyder, A. Z. , Schlaggar, B. L. , & Petersen, S. E. (2012). Spurious but systematic correlations in functional connectivity MRI networks arise from subject motion. NeuroImage, 59(3), 2142–2154. 10.1016/j.neuroimage.2011.10.018 22019881 PMC3254728

[hbm25405-bib-0080] Power, J. D. , Mitra, A. , Laumann, T. O. , Snyder, A. Z. , Schlaggar, B. L. , & Petersen, S. E. (2014). Methods to detect, characterize, and remove motion artifact in resting state fMRI. NeuroImage, 84, 320–341. 10.1016/j.neuroimage.2013.08.048 23994314 PMC3849338

[hbm25405-bib-0081] Preti, M. G. , Bolton, T. A. , & Van De Ville, D. (2017). The dynamic functional connectome: State‐of‐the‐art and perspectives. NeuroImage, 160, 41–54. 10.1016/j.neuroimage.2016.12.061 28034766

[hbm25405-bib-0082] Radtke, F. M. , Franck, M. , Lendner, J. , Krüger, S. , Wernecke, K. D. , & Spies, C. D. (2013). Monitoring depth of anaesthesia in a randomized trial decreases the rate of postoperative delirium but not postoperative cognitive dysfunction. British Journal of Anaesthesia, 110(SUPPL. 1), i98–i105. 10.1093/bja/aet055 23539235

[hbm25405-bib-0083] Raichle, M. E. , MacLeod, A. M. , Snyder, A. Z. , Powers, W. J. , Gusnard, D. A. , & Shulman, G. L. (2001). A default mode of brain function. Proceedings of the National Academy of Sciences of the United States of America, 98(2), 676–682. 10.1073/pnas.98.2.676 11209064 PMC14647

[hbm25405-bib-0084] Ranft, A. , Golkowski, D. , Kiel, T. , Riedl, V. , Kohl, P. , Rohrer, G. , … Ilg, R. (2016). Neural correlates of sevoflurane‐induced unconsciousness identified by simultaneous functional magnetic resonance imaging and electroencephalography. Anesthesiology, 125(5), 861–872. 10.1097/ALN.0000000000001322 27617689 PMC5069173

[hbm25405-bib-0085] Riehl, J. R. , Palanca, B. J. , & Ching, S. (2017). High‐energy brain dynamics during anesthesia‐induced unconsciousness. Network Neuroscience, 1(4), 431–445. 10.1162/NETN_a_00023 30090873 PMC6063715

[hbm25405-bib-0086] Rubinov, M. , & Sporns, O. (2010). Complex network measures of brain connectivity: Uses and interpretations. NeuroImage, 52(3), 1059–1069. 10.1016/j.neuroimage.2009.10.003 19819337

[hbm25405-bib-0087] Rubinov, M. , & Sporns, O. (2011). Weight‐conserving characterization of complex functional brain networks. NeuroImage, 56(4), 2068–2079. 10.1016/j.neuroimage.2011.03.069 21459148

[hbm25405-bib-0088] Saenger, V. M. , Ponce‐Alvarez, A. , Adhikari, M. , Hagmann, P. , Deco, G. , & Corbetta, M. (2018). Linking entropy at rest with the underlying structural connectivity in the healthy and lesioned brain. Cerebral Cortex, 28(8), 2948–2958. 10.1093/cercor/bhx176 28981635 PMC6248473

[hbm25405-bib-0089] Schaefer, A. , Kong, R. , Gordon, E. M. , Laumann, T. O. , Zuo, X.‐N. , Holmes, A. J. , … Yeo, B. T. T. (2018). Local‐global parcellation of the human cerebral cortex from intrinsic functional connectivity MRI. Cerebral Cortex, 28, 3095–3114. 10.1093/cercor/bhx179 28981612 PMC6095216

[hbm25405-bib-0090] Shine, J. M. , Bissett, P. G. , Bell, P. T. , Koyejo, O. , Balsters, J. H. , Gorgolewski, K. J. , … Poldrack, R. A. (2016). The dynamics of functional brain networks: Integrated network states during cognitive task performance. Neuron, 92(2), 544–554. 10.1016/j.neuron.2016.09.018 27693256 PMC5073034

[hbm25405-bib-0091] Shine, J. M. , Breakspear, M. , Bell, P. T. , Ehgoetz Martens, K. , Shine, R. , Koyejo, O. , … Poldrack, R. A. (2019). Human cognition involves the dynamic integration of neural activity and neuromodulatory systems. Nature Neuroscience, 22(2), 289–296. 10.1038/s41593-018-0312-0 30664771

[hbm25405-bib-0092] Shortal, B. P. , Hickman, L. B. , Mak‐McCully, R. A. , Wang, W. , Brennan, C. , Ung, H. , … Proekt, A. (2019). Duration of EEG suppression does not predict recovery time or degree of cognitive impairment after general anaesthesia in human volunteers. British Journal of Anaesthesia, 123(2), 206–218. 10.1016/j.bja.2019.03.046 31202561 PMC6676227

[hbm25405-bib-0093] Sporns, O. , & Betzel, R. F. (2016). Modular brain networks. Annual Review of Psychology, 67(1), 613–640. 10.1146/annurev-psych-122414-033634 PMC4782188 26393868

[hbm25405-bib-0094] Standage, D. , Areshenkoff, C. N. , Nashed, J. Y. , Matthew Hutchison, R. , Hutchison, M. , Heinke, D. , … Gallivan, J. P. (2020). Dynamic reconfiguration, fragmentation, and integration of whole‐brain modular structure across depths of unconsciousness. Cerebral Cortex, 30(10), 5229–5241. 10.1093/cercor/bhaa085 32469053 PMC7472202

[hbm25405-bib-0095] Tanabe, S. , Huang, Z. , Zhang, J. , Chen, Y. , Fogel, S. , Doyon, J. , … Northoff, G. (2020). Altered global brain signal during physiologic, pharmacologic, and pathologic states of unconsciousness in humans and rats. Anesthesiology, 132, 1392–1406. 10.1097/ALN.0000000000003197 32205548 PMC8218242

[hbm25405-bib-0096] Threlkeld, Z. D. , Bodien, Y. G. , Rosenthal, E. S. , Giacino, J. T. , Nieto‐Castanon, A. , Wu, O. , … Edlow, B. L. (2018). Functional networks reemerge during recovery of consciousness after acute severe traumatic brain injury. Cortex, 106, 2–11. 10.1016/j.cortex.2018.05.004 PMC6120794 29871771

[hbm25405-bib-0097] Tian, Y. , Margulies, D. , Breakspear, M. , & Zalesky, A. (2020). Topographic organization of the human subcortex unveiled with functional connectivity gradients. Nature Neuroscience, 23(11), 1421–1432. 10.1101/2020.01.13.903542 32989295

[hbm25405-bib-0098] Tononi, G. (2004). An information integration theory of consciousness: An information integration theory of consciousness. BMC Neuroscience, 5, 42–64. 10.1186/1471-2202-5-42 15522121 PMC543470

[hbm25405-bib-0099] Tononi, G. , & Edelman, G. M. (1998). Consciousness and complexity. Science, 282, 1846–1851. 10.1126/science.282.5395.1846 9836628

[hbm25405-bib-0100] Tononi, G. , Sporns, O. , & Edelman, G. M. (1994). A measure for brain complexity: Relating functional segregation and integration in the nervous system. Proceedings of the National Academy of Sciences of the United States of America, 91(11), 5033–5037. 10.1073/pnas.91.11.5033 8197179 PMC43925

[hbm25405-bib-0101] Uhrig, L. , Sitt, J. D. , Jacob, A. , Tasserie, J. , Barttfeld, P. , Dupont, M. , … Jarraya, B. (2018). Resting‐state dynamics as a cortical signature of anesthesia in monkeys. Anesthesiology, 129(5), 942–958. 10.1097/ALN.0000000000002336 30028727

[hbm25405-bib-0102] van Dijk, K. R. A. , Sabuncu, M. R. , & Buckner, R. L. (2012). The influence of head motion on intrinsic functional connectivity MRI. NeuroImage, 59(1), 431–438. 10.1016/j.neuroimage.2011.07.044 21810475 PMC3683830

[hbm25405-bib-0103] Varley, T. F. , Denny, V. , Sporns, O. , & Patania, A. (2020). Topological analysis of differential effects of ketamine and propofol anesthesia on brain dynamics. *bioRxiv* . 10.1101/2020.04.04.025437 PMC8220281 34168888

[hbm25405-bib-0104] Varley, T. F. , Luppi, A. I. , Pappas, I. , Naci, L. , Adapa, R. , Owen, A. M. , … Stamatakis, E. A. (2020). Consciousness & brain functional complexity in propofol anaesthesia. Scientific Reports, 10(1), 1018. 10.1038/s41598-020-57695-3 31974390 PMC6978464

[hbm25405-bib-0105] Vlisides, P. E. , Li, D. , Zierau, M. , Lapointe, A. P. , Ip, K. I. , McKinney, A. M. , & Mashour, G. A. (2019). Dynamic cortical connectivity during general anesthesia in surgical patients. Anesthesiology, 130(6), 885–897. 10.1097/ALN.0000000000002677 30946057 PMC6520139

[hbm25405-bib-0106] Vohryzek, J. , Deco, G. , Cessac, B. , Kringelbach, M. L. , & Cabral, J. (2020). Ghost attractors in spontaneous brain activity: Recurrent excursions into functionally‐relevant BOLD phase‐locking states. Frontiers in Systems Neuroscience, 14, 20. 10.3389/fnsys.2020.00020 32362815 PMC7182014

[hbm25405-bib-0107] Wenzel, M. , Han, S. , Smith, E. H. , Hoel, E. , Greger, B. , House, P. A. , & Yuste, R. (2019). Reduced repertoire of cortical microstates and neuronal ensembles in medically induced loss of consciousness. Cell Systems, 8(5), 467–474.e4. 10.1016/j.cels.2019.03.007 31054810 PMC6544156

[hbm25405-bib-0108] Whitfield‐Gabrieli, S. , & Nieto‐Castanon, A. (2012). Conn: A functional connectivity toolbox for correlated and anticorrelated brain networks. Brain Connectivity, 2(3), 125–141. 10.1089/brain.2012.0073 22642651

[hbm25405-bib-0109] Yeo, B. T. T. , Krienen, F. M. , Sepulcre, J. , Sabuncu, M. R. , Lashkari, D. , Hollinshead, M. , … Buckner, R. L. (2011). The organization of the human cerebral cortex estimated by intrinsic functional connectivity. Journal of Neurophysiology, 106, 1125–1165. 10.1152/jn.00338.2011 21653723 PMC3174820

[hbm25405-bib-0110] Zalesky, A. , Fornito, A. , & Bullmore, E. T. (2010). Network‐based statistic: Identifying differences in brain networks. NeuroImage, 53(4), 1197–1207. 10.1016/j.neuroimage.2010.06.041 20600983

[hbm25405-bib-0111] Zalesky, A. , Fornito, A. , Cocchi, L. , Gollo, L. L. , & Breakspear, M. (2014). Time‐resolved resting‐state brain networks. Proceedings of the National Academy of Sciences of the United States of America, 111(28), 10341–10346. 10.1073/pnas.1400181111 24982140 PMC4104861

[hbm25405-bib-0112] Zamani Esfahlani, F. , Jo, Y. , Faskowitz, J. , Byrge, L. , Kennedy, D. P. , Sporns, O. , & Betzel, R. F. (2020). High‐amplitude cofluctuations in cortical activity drive functional connectivity. Proceedings of the National Academy of Sciences of the United States of America, 117(45), 28393–28401. 10.1073/pnas.2005531117 33093200 PMC7668041

[hbm25405-bib-0113] Zhang, J. , Huang, Z. , Chen, Y. , Zhang, J. , Ghinda, D. , Nikolova, Y. , … Northoff, G. (2018). Breakdown in the temporal and spatial organization of spontaneous brain activity during general anesthesia. Human Brain Mapping, 39(5), 2035–2046. 10.1002/hbm.23984 29377435 PMC6866328

[hbm25405-bib-0114] Zhang, Y. , Wang, C. , Wang, Y. , Yan, F. , Wang, Q. , & Huang, L. (2019). Investigating dynamic functional network patterns after propofol‐induced loss of consciousness. Clinical Neurophysiology, 130(3), 331–340. 10.1016/j.clinph.2018.11.028 30665155

[hbm25405-bib-0115] Zhang, Y. , Wang, Y. , Yan, F. , Song, D. , Wang, H. , Wang, Q. , & Huang, L. (2020). Influence of pre‐anesthesia dynamic frontal‐parietal communication on individual susceptibility to propofol. Clinical Neurophysiology, 131(11), 2566–2577. 10.1016/j.clinph.2020.07.018 32927212

