# Supplementary material for: Brain network integration dynamics are associated with loss and recovery of consciousness induced by sevoflurane
Source: Hum Brain Mapp. 2021 Mar 19;42(9):2802–22. doi: 10.1002/hbm.25405 (PMC8127159; doi:10.1002/hbm.25405)
Supplement: Supplementary file 1 — Appendix S1: Supplementary Information [file HBM-42-2802-s001.docx]

# Supporting Information for

# Brain network integration dynamics are associated with loss and recovery of consciousness induced by sevoflurane

Andrea I. Luppi^a,b^*, Daniel Golkowski^c^, Andreas Ranft^d^, Rüdiger Ilg^c,e^, Denis Jordan^d^, David K. Menon^a,f^, Emmanuel A. Stamatakis^a^

^a^Division of Anaesthesia, University of Cambridge, Cambridge, UK

^b^Department of Clinical Neurosciences, University of Cambridge, Cambridge, UK

^c^Department of Neurology, Klinikum rechts der Isar, Technical University Munich, München, Germany

^d^Department of Anaesthesiology and Intensive Care Medicine, Klinikum rechts der Isar, Technical University Munich, München, Germany

^e^Asklepios Clinic, Department of Neurology, Bad Tölz, Germany

^f^ Wolfon Brain Imaging Centre, University of Cambridge, Cambridge, UK

# Supplementary Materials and Methods

### Modularity

In the case of signed graphs, a module is defined as a group of nodes that are positively correlated with each other, but negatively correlated with nodes belonging to different modules (Rubinov & Sporns, 2010). The modularity function *Q* quantifies how well a network can be divided into distinct modules, and is computed as follows:

$$Q = \frac{1}{\upsilon^{+}}\sum_{ij} ({w_{ij}}^{+} - {e_{ij}}^{+})\delta_{M_{i}M_{j}}- \frac{1}{\upsilon^{+}+ \upsilon^{-}}({w_{ij}}^{-} - {e_{ij}}^{-})\delta_{M_{i}M_{j}}$$

where *υ* is the total weight of the graph (sum of all edges), *w_ij_* is the (signed) weight of the edge between nodes *i* and *j*, *e_ij_* is the weight of an edge divided by the total weight of the graph (positive and negative edges are denoted with ‘+’ and ‘−’ superscripts, respectively), and *δ_MiMj_* is set to 1 when nodes *i* and *j* are in the same module and 0 otherwise.

### Participation coefficient

The participation coefficient *P_i_* quantifies the degree of connection that a node entertains with nodes belonging to other modules: the more of a node’s connections are towards other modules, the higher its participation coefficient will be (Rubinov & Sporns, 2010). Conversely, the participation coefficient of a node will be zero if its connections are all with nodes belonging to its own module.

$$P_{i}= 1 - \sum_{s=1}^{M} \left( \frac{\kappa_{is}}{k_{i}} \right)^{2}$$

Here, *κ_is_* is the strength of positive connections between node *i* and other nodes in module *s*, *k_i_* is the strength of all its positive connections, and *M* is the number of modules in the network, as identified by a given modularity detection algorithm. The participation coefficient ranges between zero (no connections with other modules) and one (equal connections to all other modules). A network with high average participation coefficient can be expected to have high levels of integration between its constituent modules.

### Within-module degree Z-score

The Within-module degree Z-score *Z_i_* is a measure of a node’s connectivity with other nodes belonging to its module. It indicates how much larger (or smaller) the node’s connections to other nodes in the module are, relative to the average connection strength within that module. A node with high within-module degree Z-score has stronger-than-average coupling with the other nodes in its module (Rubinov & Sporns, 2010).

$$z_{i} = \frac{\kappa_{is} - \bar{\kappa}_{is}}{\sigma_{\kappa_{is}}}$$

where *κ_is_* is the strength of connections between node *i* and other nodes in module *s*, and $\bar{\kappa}$*_is_* and *σ_κis_* are respectively the average and the standard deviation of *κ_is_* over all nodes belonging to module *s*.

### Characteristic path length

Characteristic path length (*L*) is a network-wide measure of how effortful it is on average to move between different nodes in the network. This metric is calculated as the average length of the shortest path *d* between every pair of nodes in the network.

$$L = \frac{1}{n} \sum_{i}^{n} \frac{\sum_{j\neq i}^{n} \left( d_{ij} \right)}{n-1}$$

The shortest path length between two nodes *i* and *j*, *d_ij_,* represents the effort required to move between them, such that smaller values indicate less effort/easier communication. Different networks may attach different meaning to the quantity being minimised, such as time in a transport network, or cost. In the simplified case of a binarised network, the shortest path (geodesic distance) between two nodes *i* and *j* is the smallest number of edges that need to be traversed to move from *i* to *j*. When considering a weighted network, a stronger edge is understood as implying easier communication between the two nodes (analogous to how large a road is: larger roads make communication easier). Thus, in weighted graphs the shortest path d between *i* and *j* is calculated as the minimum sum of inverse of the edge weights that need to be traversed between the two nodes. Thus, the characteristic path length is understood as inversely related to the capacity for global processing across the whole network (Rubinov & Sporns, 2010).

### Clustering coefficient

The clustering coefficient of node *i* (*C_i_*) is a node-specific measure of how well connected a node’s neighbourhood is; in a binarized graph, it is calculated as the fraction of neighbours of the node that are also neighbours of each other.

$$C_{i} =\frac{{2t}_{i}}{k_{i}(k_{i} - 1)}$$

where *t_i_* is the number of triangles around node *i,*and $k_{i}$ is the number of edges connected to node *i.*”

Following (Muldoon et al., 2016), for weighted graphs we adopt Onnela’s generalisation of the clustering coefficient based on subgraph intensity (Onnela, Saramäki, Kertész, & Kaski, 2005):

$$C_{i} =\frac{1}{k_{i}(k_{i} - 1)}\sum_{j,k}^{n} \left( \hat{w}_{ij} + \hat{w}_{jk} + \hat{w}_{ik} \right)^{1/3}$$

With *w_ij_* being the strength of a connection between nodes *i* and *j*, and $\hat{w}_{ij}=$*w_ij_* / max(*w*).

The mean of all nodes’ clustering coefficients (i.e. the network’s mean clustering coefficient, *C*), indicates how well connected, on average, the neighbourhoods present in the network tend to be. When applied to brain networks, the clustering coefficient is thought to represent the degree of information integration at a local level, and hence the potential for efficiently performing specialised local processing (Rubinov & Sporns, 2010).

# Supplementary Figures


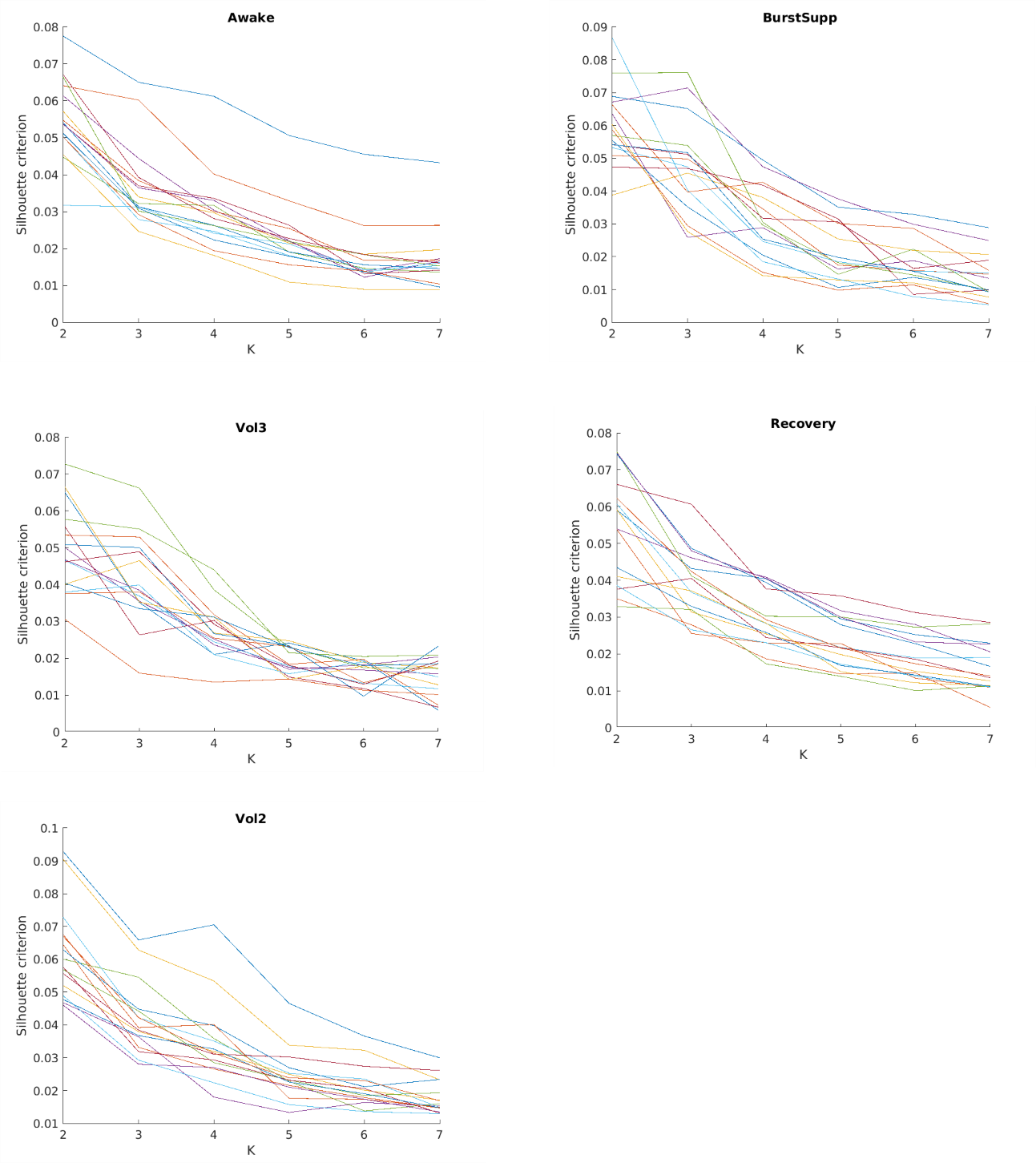


### **Supplementary Figure 1.** Silhouette criterion for the choice of best number of clusters K for each participant, separately for each condition. In almost every case, K = 2 is the most appropriate number of clusters.


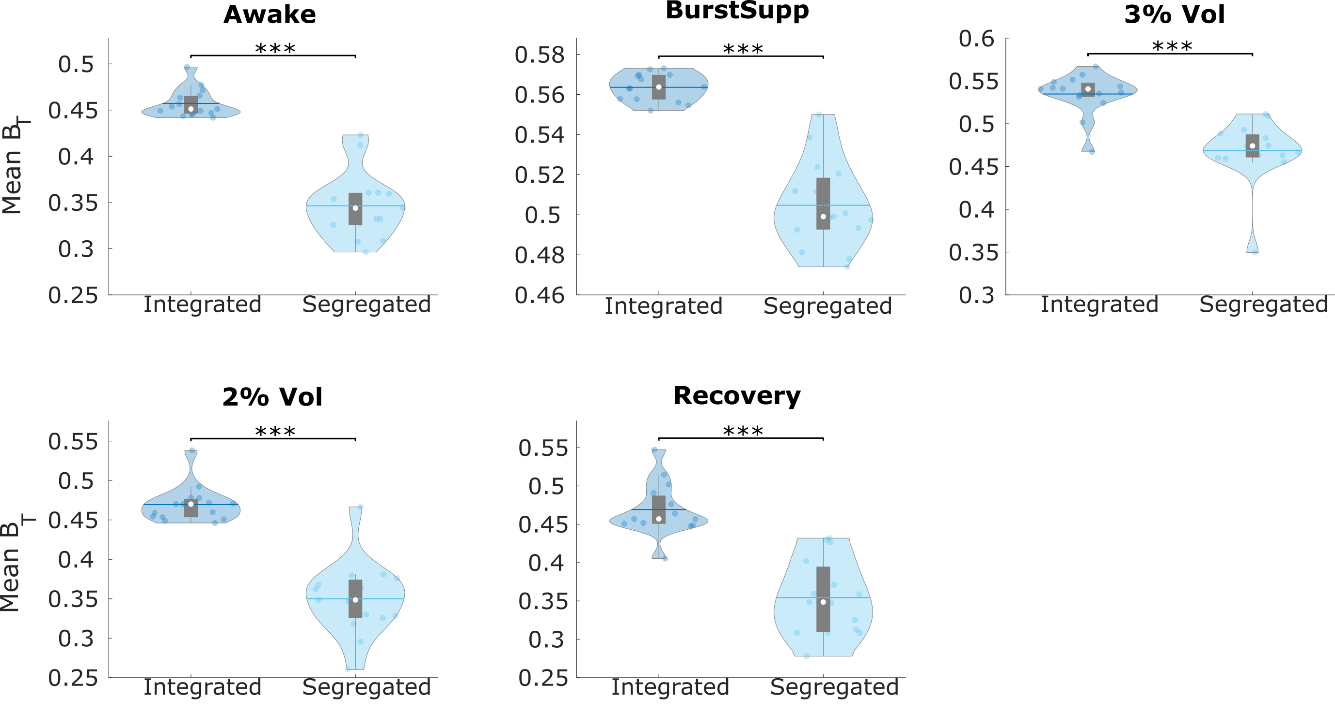


**Supplementary Figure 2. Significant differences in mean participation coefficient *B_T_* between integrated and segregated sub-states, for each condition.** Violin plots indicate the distribution of participants in each condition (coloured circles). White circle, median; horizontal center line, mean; box limits, upper and lower quartiles; whiskers, 1.5x interquartile range. **** p* < 0.001.


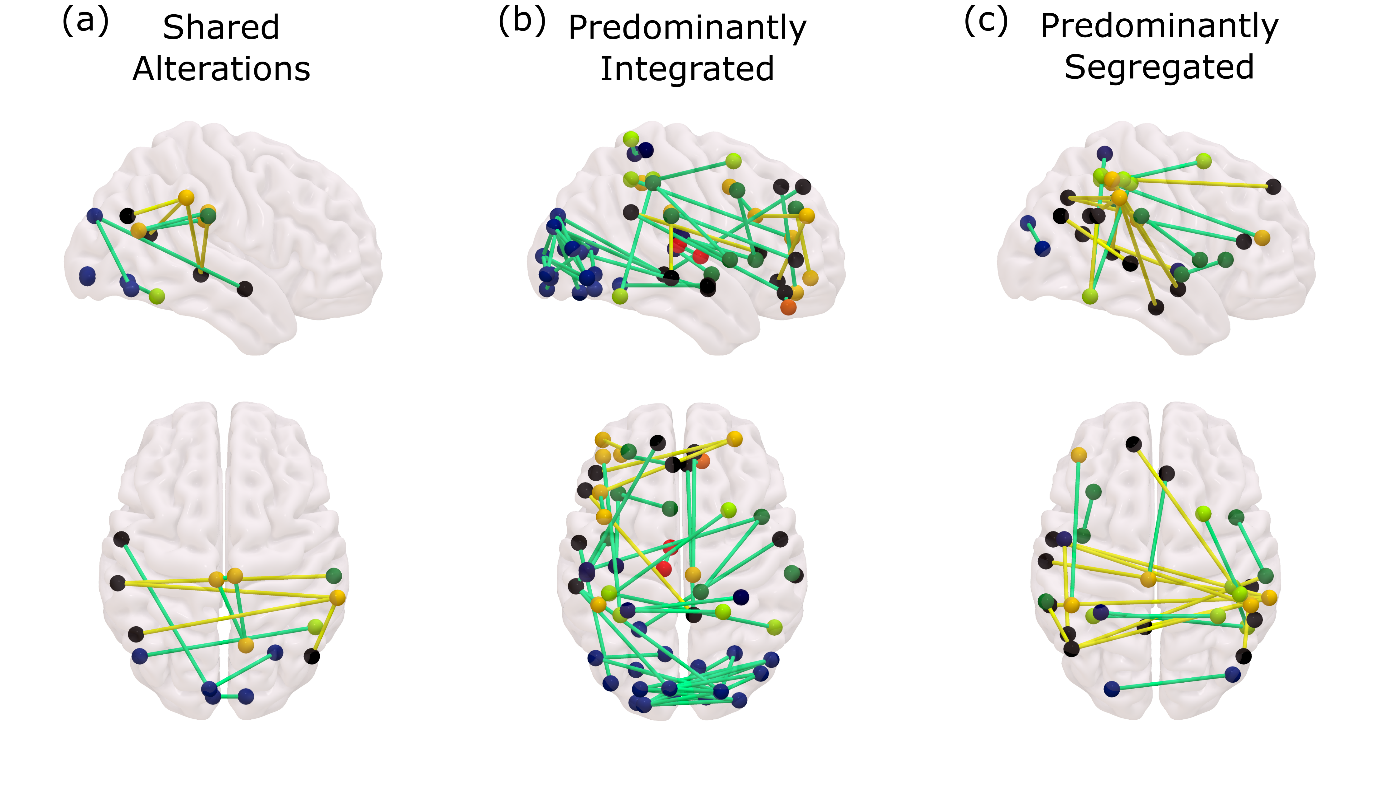
**Supplementary Figure 3.** **Replication of NBS results with extent-based threshold.** (a) Sagittal and axial brain projections of connections that are significantly different from each conscious state (wakefulness and recovery) during every unconscious state (burst-suppression, 3% volume and 2% volume of sevoflurane), for both the integrated and segregated sub-states. (b) Sagittal and axial brain projections of connections that are significantly different from each conscious state (wakefulness and recovery) during every unconscious state (burst-suppression, 3% volume and 2% volume of sevoflurane), exclusively for the integrated sub-state. (c) Sagittal and axial brain projections of connections that are significantly different from each conscious state (wakefulness and recovery) during every unconscious state (burst-suppression, 3% volume and 2% volume of sevoflurane), exclusively for the segregated sub-state. Yellow edges: conscious > unconscious. Green edges: unconscious > conscious. Black nodes: default mode network. Light blue: visual network. Dark blue: sensorimotor network. Dark green: salience/ventral attention network. Light green: dorsal attention network. Yellow: fronto-parietal network. Red: subcortical network. ROIs were assigned to resting-state network by Fan et al (2016) based on the definitions of Yeo *et al* (2011). Statistical significance between each pair of conditions was determined by the NBS with an extent-based F-threshold of 10.


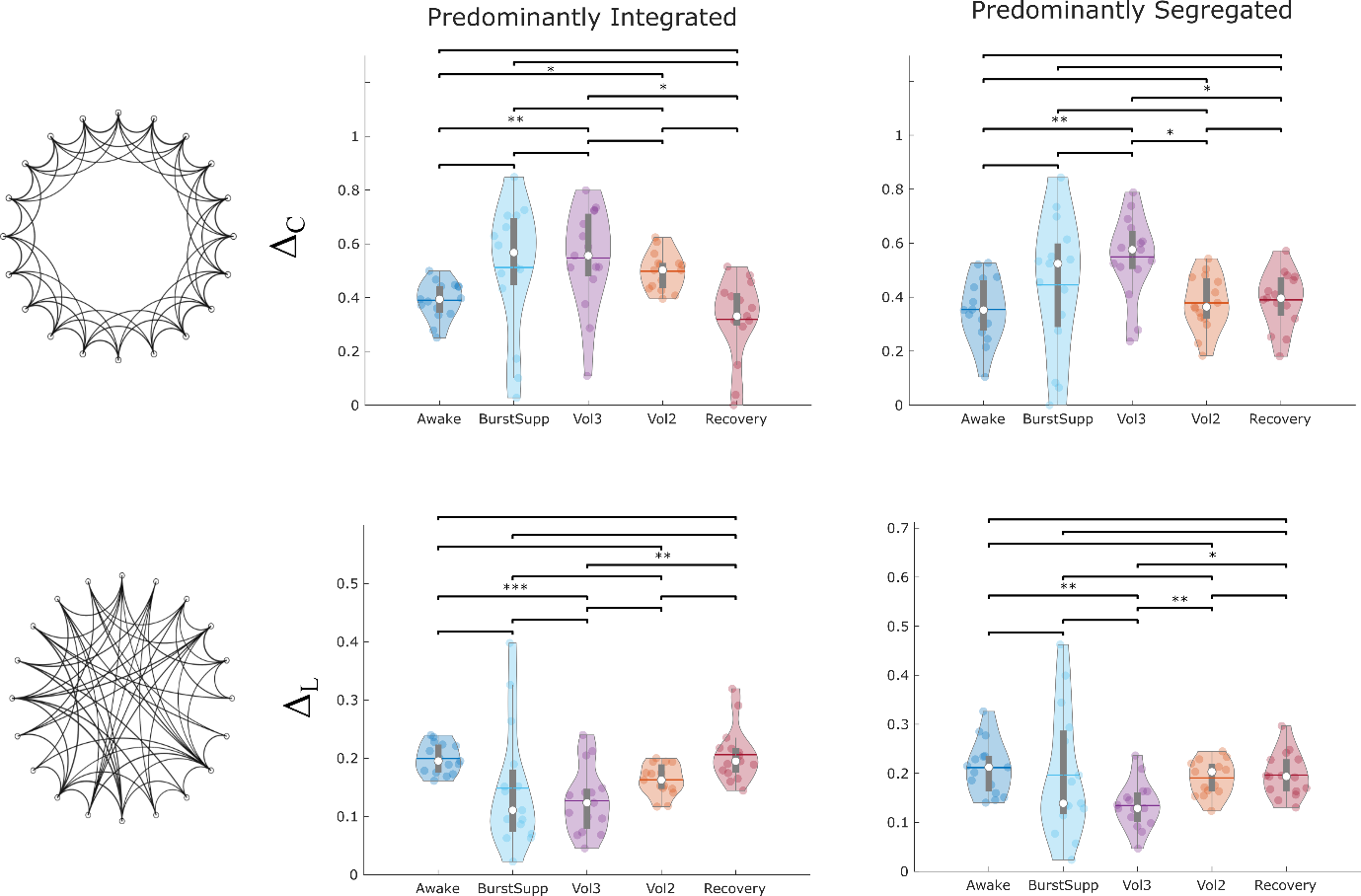


### **Supplementary Figure 4. Brain network deviation from regular and random.** Comparison of deviation from a regular (lattice) network in terms of clustering (Δ_C_; top row) and deviation from a random network in terms of characteristic path length (Δ_L_), separately for the predominantly integrated and predominantly segregated sub-states of dynamic functional connectivity, for the augmented Schaefer-232 parcellation.


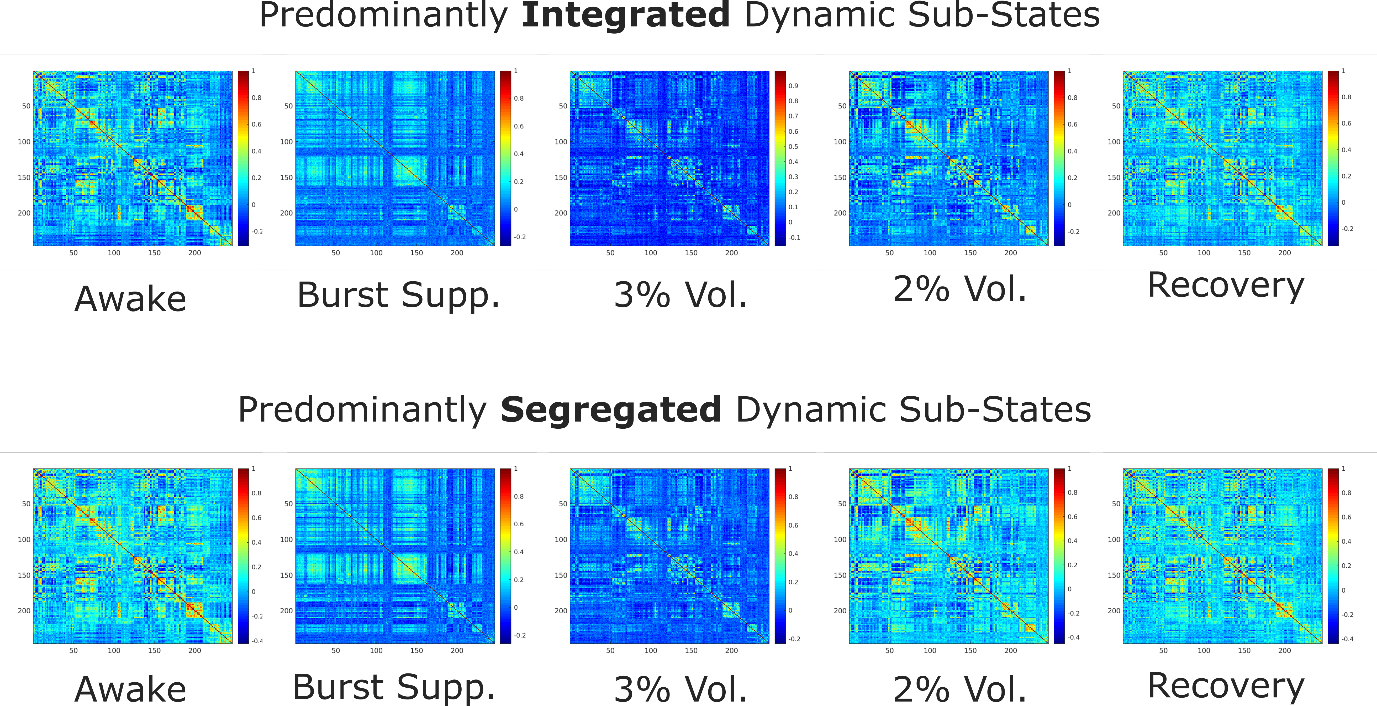


### **Supplementary Figure 5. Connectivity matrices for the integrated and segregated sub-states of dynamic functional connectivity, for the Brainnetome-246 atlas.**


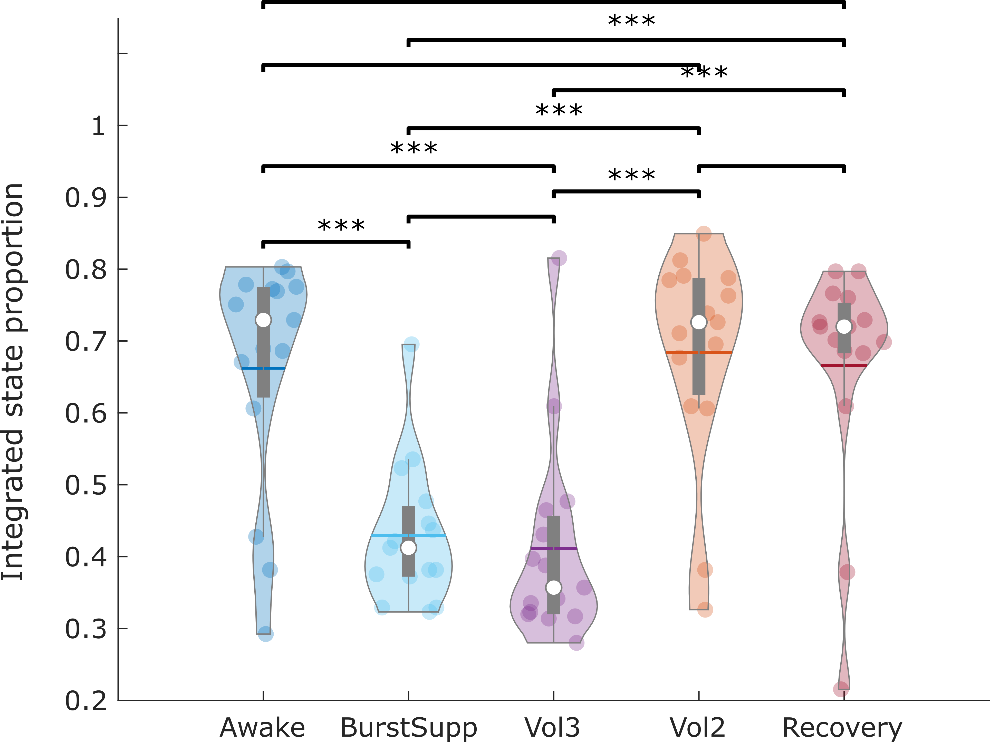


### **Supplementary Figure 6. Reduced time spent in the integrated sub-state at deep levels of sevoflurane anaesthesia is replicated with Brainnetome atlas.** Violin plots indicate the distribution of participants in each condition (coloured circles). White circle, median; horizontal center line, mean; box limits, upper and lower quartiles; whiskers, 1.5x interquartile range. *** p < 0.001, Bonferroni-corrected.


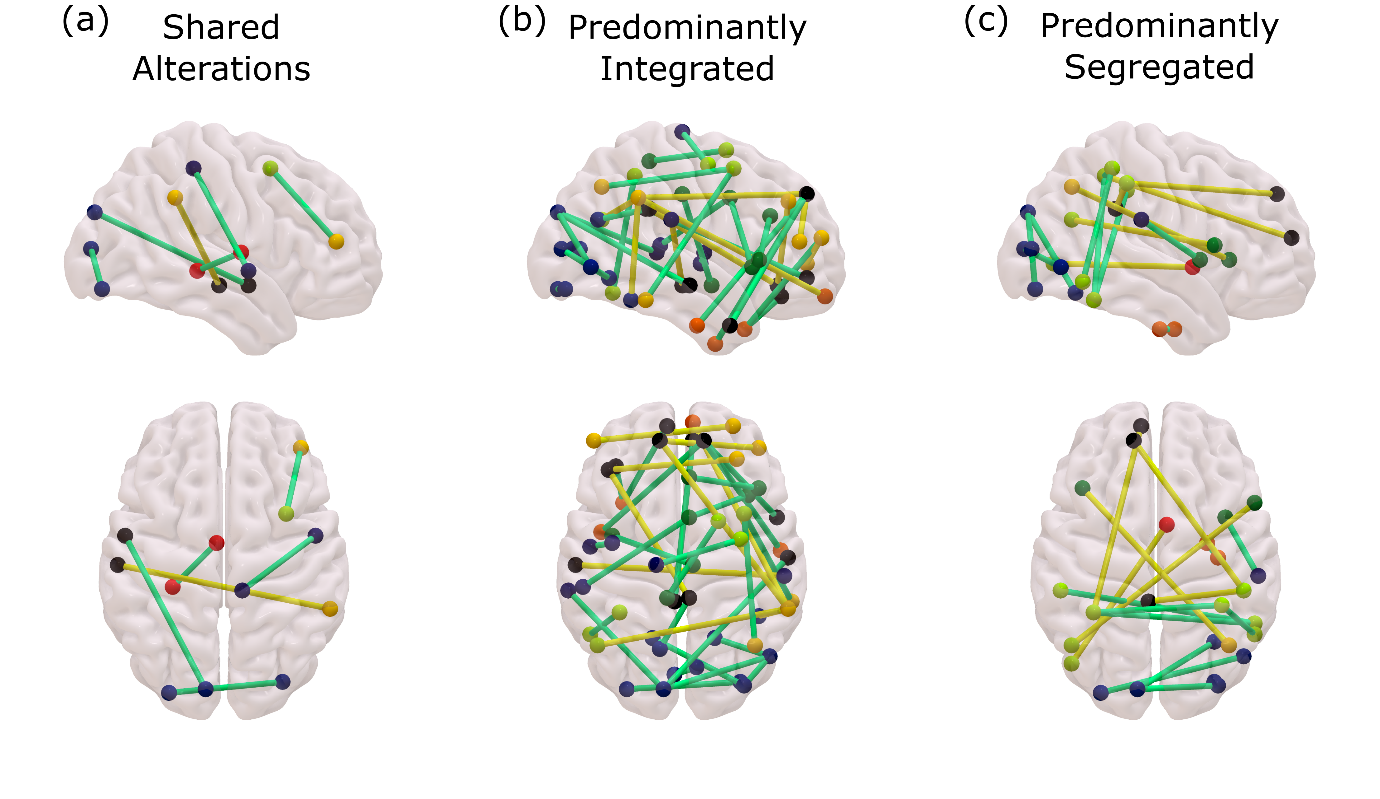


### **Supplementary Figure 7.** **Consciousness-specific dynamic reorganisation of functional connectivity induced by sevoflurane is replicated with Brainnetome atlas.** (a) Sagittal and axial brain projections of connections that are significantly different from each conscious state (wakefulness and recovery) during every unconscious state (burst-suppression, 3% volume and 2% volume of sevoflurane), for both the integrated and segregated sub-states. (b) Sagittal and axial brain projections of connections that are significantly different from each conscious state (wakefulness and recovery) during every unconscious state (burst-suppression, 3% volume and 2% volume of sevoflurane), exclusively for the integrated sub-state. (c) Sagittal and axial brain projections of connections that are significantly different from each conscious state (wakefulness and recovery) during every unconscious state (burst-suppression, 3% volume and 2% volume of sevoflurane), exclusively for the segregated sub-state. Yellow edges: conscious > unconscious. Green edges: unconscious > conscious. Black nodes: default mode network. Light blue: visual network. Dark blue: sensorimotor network. Dark green: salience/ventral attention network. Light green: dorsal attention network. Yellow: fronto-parietal network. Red: subcortical network. ROIs were assigned to resting-state network by Fan et al (2016) based on the definitions of Yeo *et al* (2011). Statistical significance between each pair of conditions was determined by the NBS with an intensity-based F-threshold of 10.


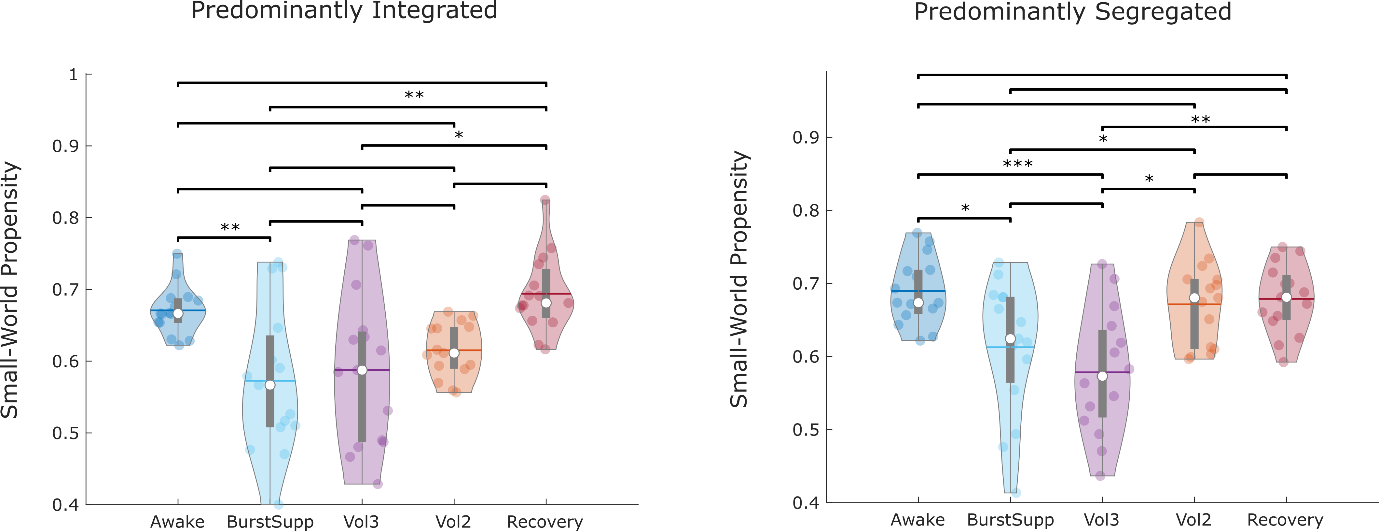


### **Supplementary Figure 8. Reduced small-world propensity of dynamic brain networks under sevoflurane is replicated with Brainnetome atlas.** Violin plots represent the distribution of small-world propensity for the predominantly integrated sub-state, and the predominantly segregated dynamic sub-state, across levels of sevoflurane. White circle, median; horizontal center line, mean; box limits, upper and lower quartiles; whiskers, 1.5x interquartile range. * *p* < 0.05; ** *p* < 0.01; *** *p* < 0.001, after Bonferroni correction.


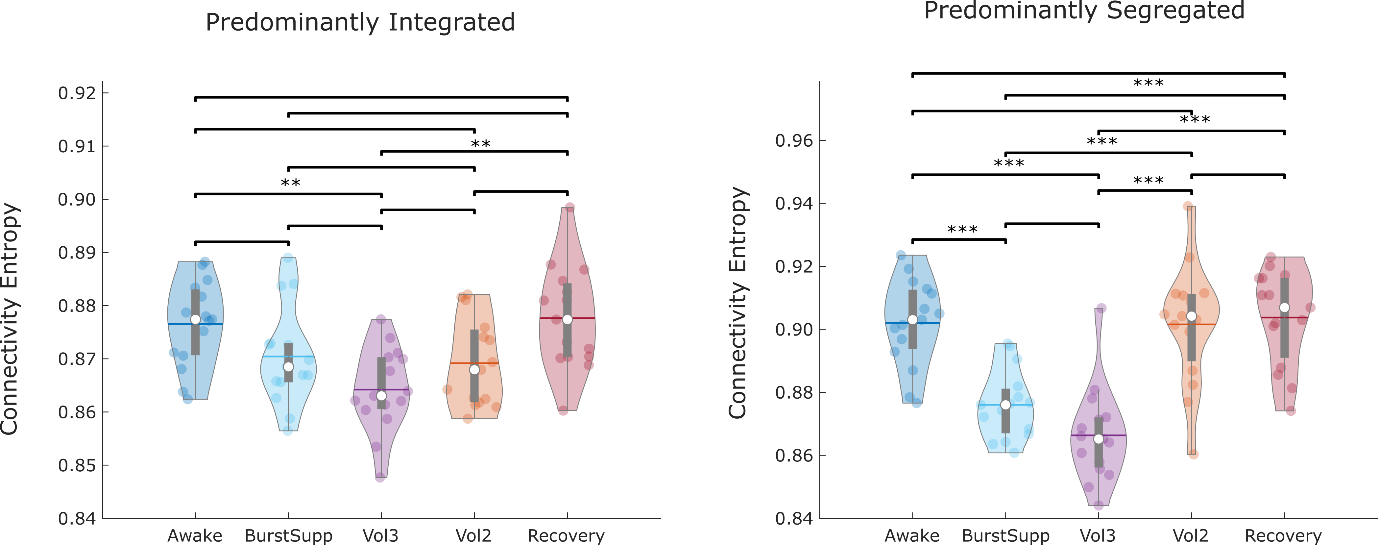


### **Supplementary Figure 9. Reduced entropy of functional connections between brain regions under the effects of sevoflurane, is replicated with Brainnetome atlas.** Violin plots represent the distribution of connectivity entropy across the whole brain for the predominantly integrated sub-state, and the predominantly segregated dynamic sub-state, across levels of sevoflurane. White circle, median; horizontal center line, mean; box limits, upper and lower quartiles; whiskers, 1.5x interquartile range. ** *p* < 0.01; *** *p* < 0.001, after Bonferroni correction.


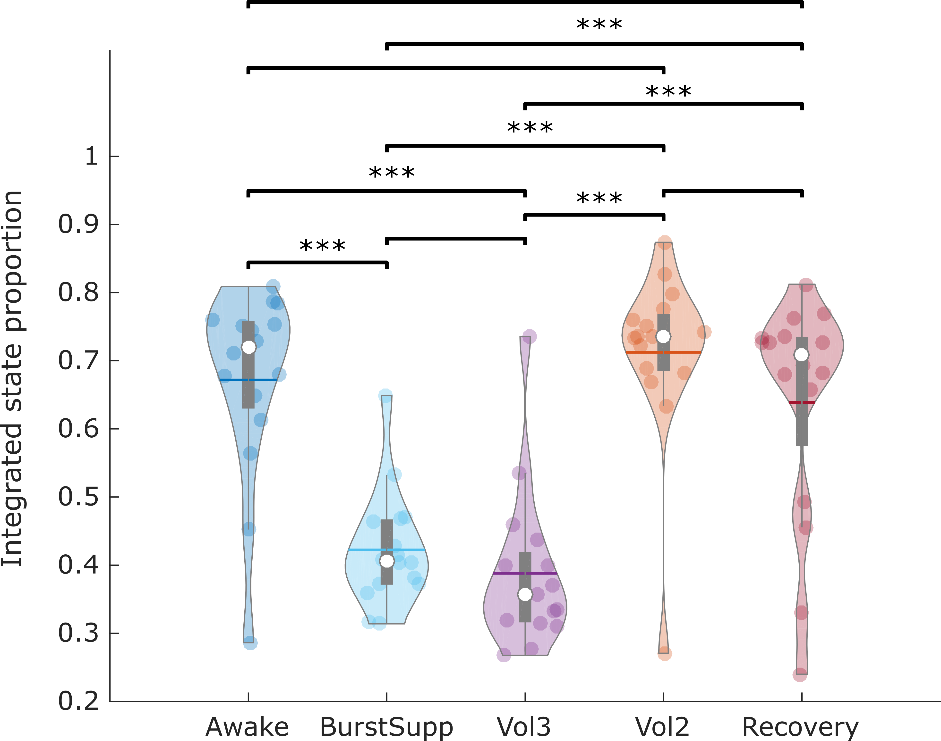


### **Supplementary Figure 10. Reduced time is spent in the integrated sub-state at deep levels of sevoflurane anaesthesia, is replicated when including all subjects.** Violin plots indicate the distribution of participants in each condition (coloured circles). White circle, median; horizontal center line, mean; box limits, upper and lower quartiles; whiskers, 1.5x interquartile range. *** p < 0.001, Bonferroni-corrected.


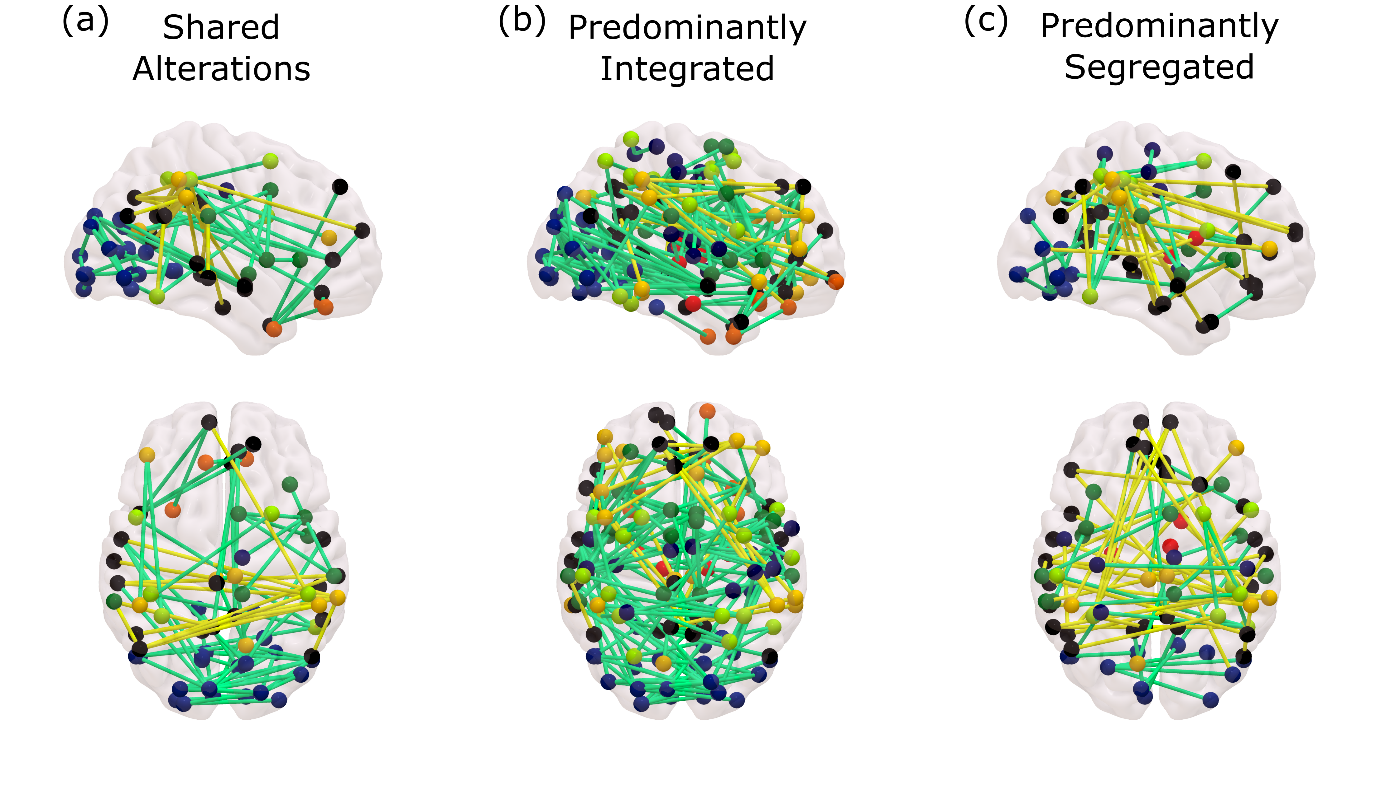
**Supplementary Figure 11.** **Consciousness-specific dynamic reorganisation of functional connectivity induced by sevoflurane, is replicated when including all subjects.** (a) Sagittal and axial brain projections of connections that are significantly different from each conscious state (wakefulness and recovery) during every unconscious state (burst-suppression, 3% volume and 2% volume of sevoflurane), for both the integrated and segregated sub-states. (b) Sagittal and axial brain projections of connections that are significantly different from each conscious state (wakefulness and recovery) during every unconscious state (burst-suppression, 3% volume and 2% volume of sevoflurane), exclusively for the integrated sub-state. (c) Sagittal and axial brain projections of connections that are significantly different from each conscious state (wakefulness and recovery) during every unconscious state (burst-suppression, 3% volume and 2% volume of sevoflurane), exclusively for the segregated sub-state. Yellow edges: conscious > unconscious. Green edges: unconscious > conscious. Black nodes: default mode network. Light blue: visual network. Dark blue: sensorimotor network. Dark green: salience/ventral attention network. Light green: dorsal attention network. Yellow: fronto-parietal network. Red: subcortical network. ROIs were assigned to resting-state network by Fan et al (2016) based on the definitions of Yeo *et al* (2011). Statistical significance between each pair of conditions was determined by the NBS with an intensity-based F-threshold of 10.


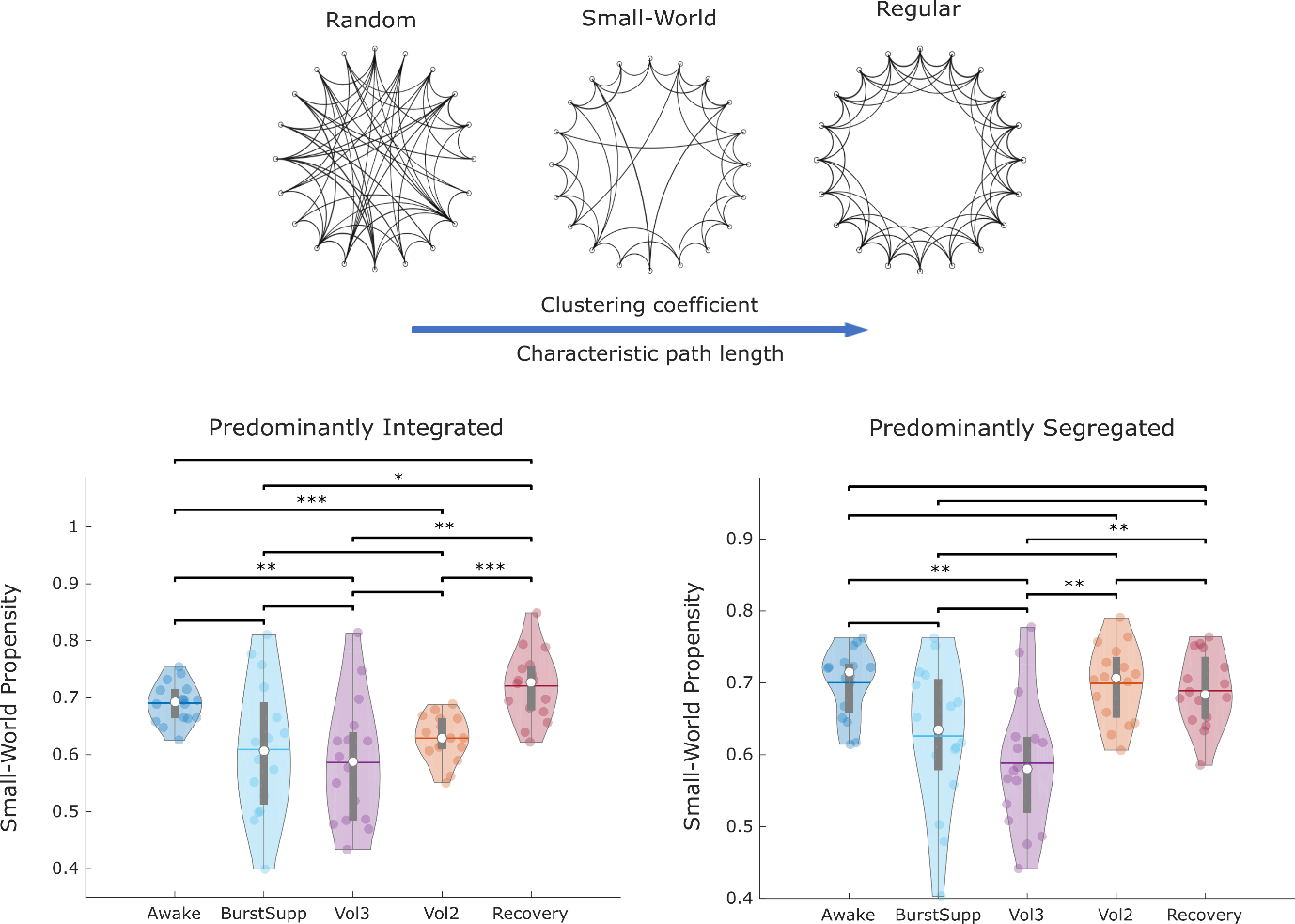


### **Supplementary Figure 12. Reduced small-world propensity of dynamic brain networks under sevoflurane, is replicated when including all subjects.** Violin plots represent the distribution of small-world propensity for the predominantly integrated sub-state, and the predominantly segregated dynamic sub-state, across levels of sevoflurane. White circle, median; horizontal center line, mean; box limits, upper and lower quartiles; whiskers, 1.5x interquartile range. * *p* < 0.05; ** *p* < 0.01; *** *p* < 0.001, after Bonferroni correction.


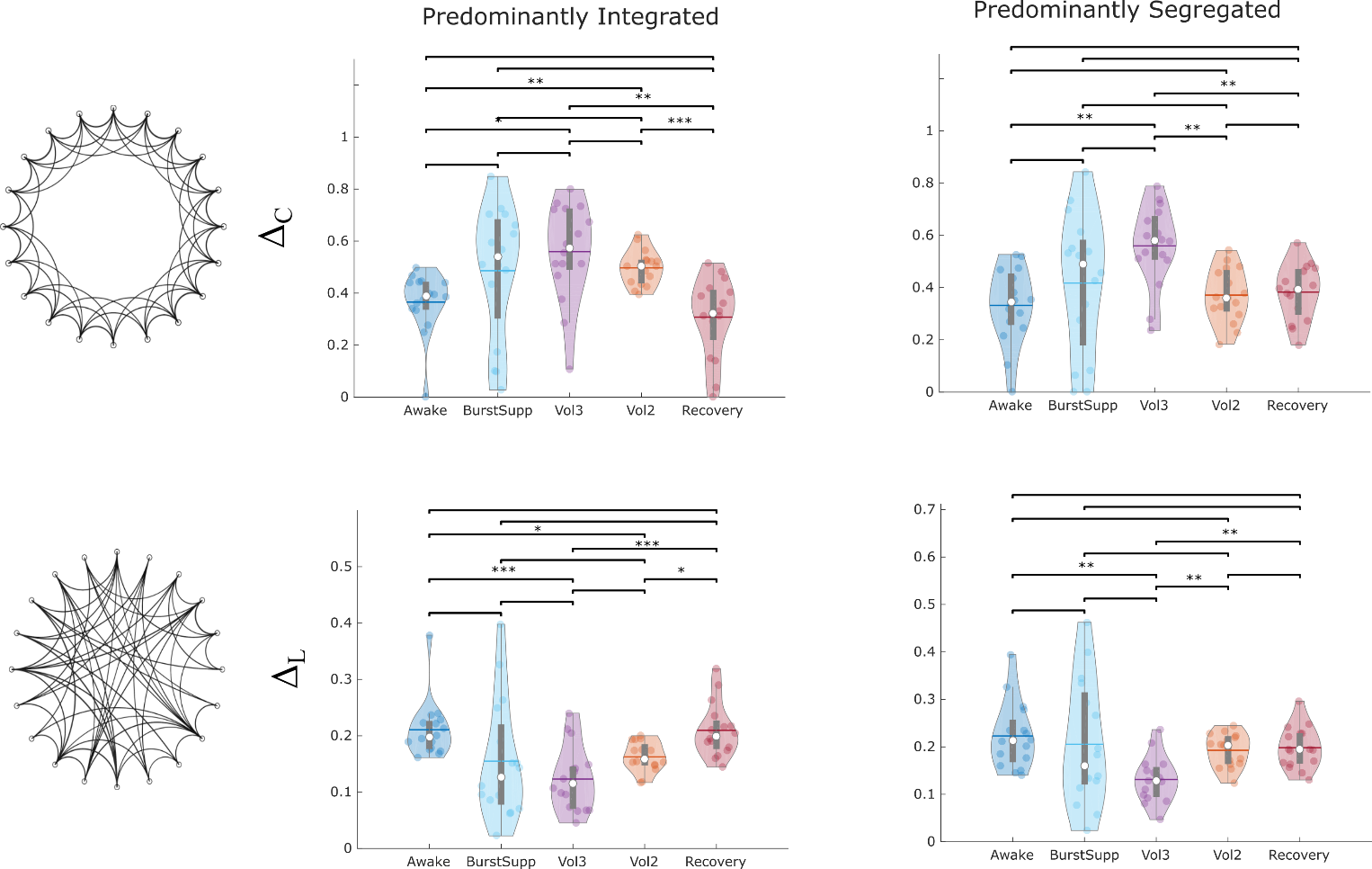


### **Supplementary Figure 13. Brain network deviation from regular and random under sevoflurane, is replicated when including all subjects.** Comparison of deviation from a regular (lattice) network in terms of clustering (Δ_C_; top row) and deviation from a random network in terms of characteristic path length (Δ_L_), separately for the predominantly integrated and predominantly segregated sub-states of dynamic functional connectivity, for the augmented Schaefer-232 parcellation.


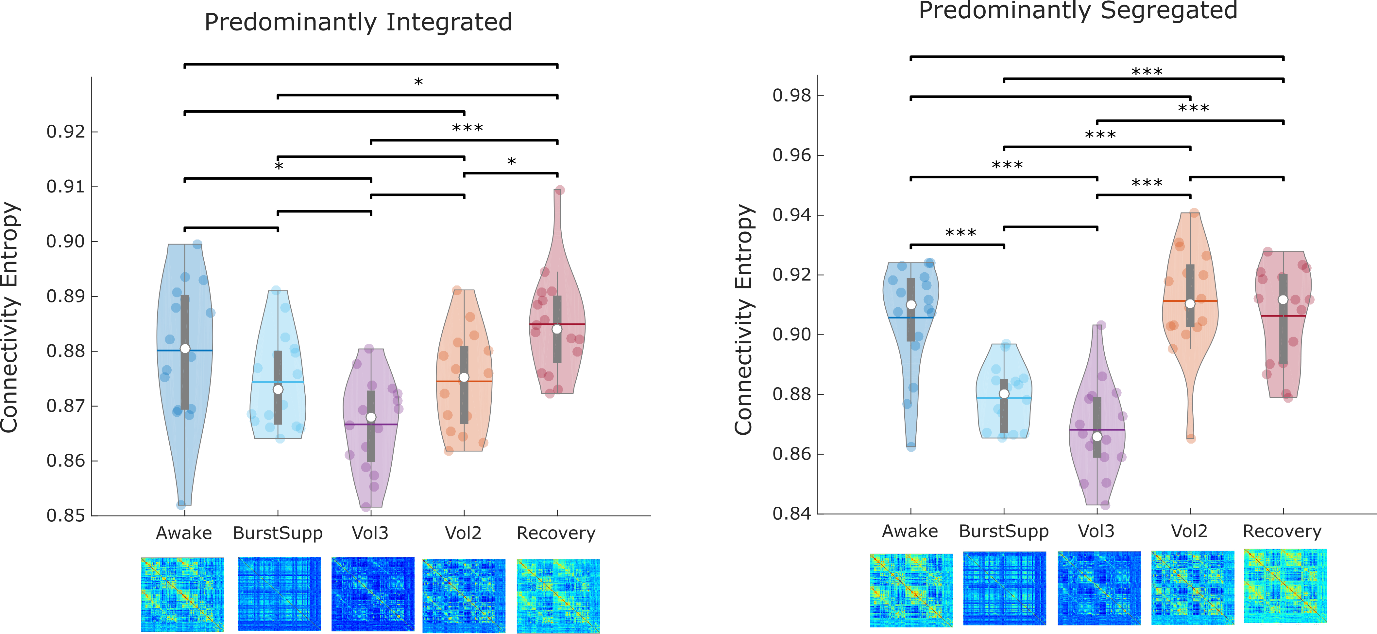


### **Supplementary Figure 14. Reduced entropy of functional connections between brain regions under the effects of sevoflurane, is replicated when including all subjects.** Violin plots represent the distribution of connectivity entropy across the whole brain for the predominantly integrated sub-state, and the predominantly segregated dynamic sub-state, across levels of sevoflurane. White circle, median; horizontal center line, mean; box limits, upper and lower quartiles; whiskers, 1.5x interquartile range. * *p* < 0.05; ** *p* < 0.01; *** *p* < 0.001, after Bonferroni correction.


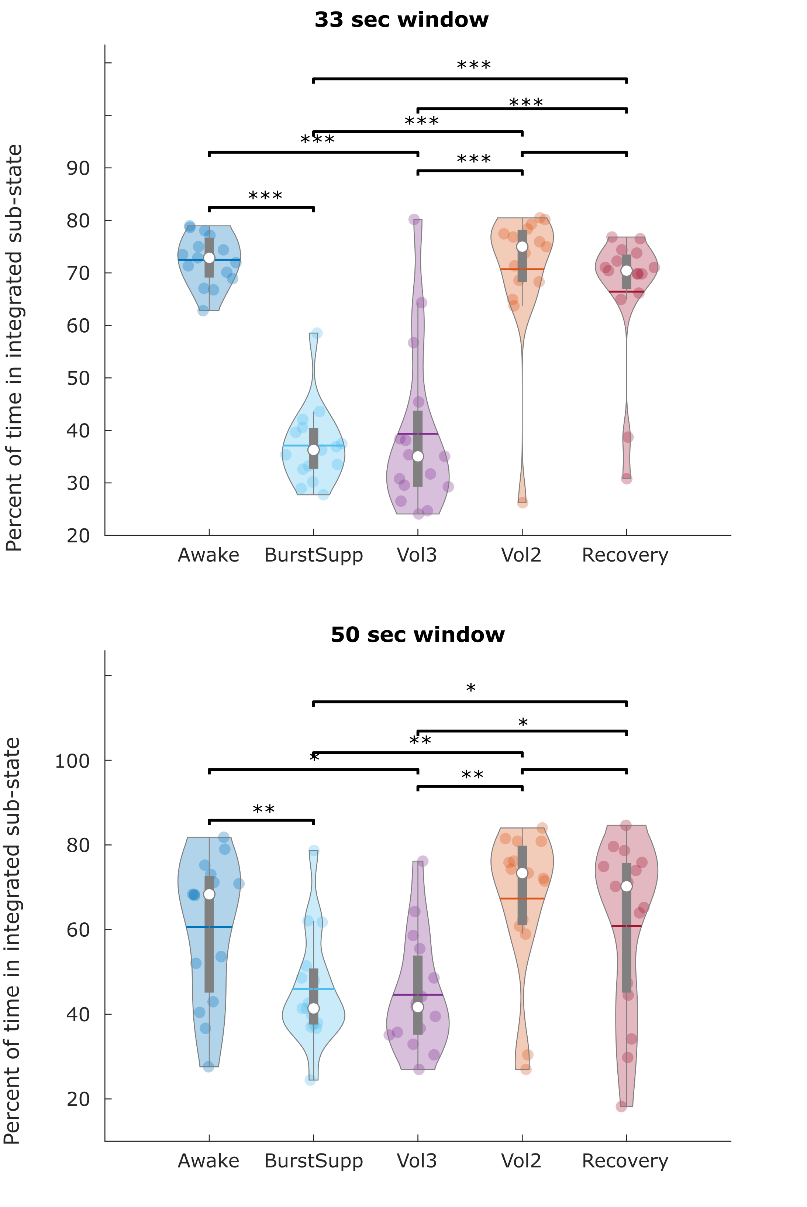


### **Supplementary Figure 15. Reduced time is spent in the integrated sub-state at deep levels of sevoflurane anaesthesia, is replicated when using shorter sliding windows (18 TRs, ~33s, top) or longer sliding windows (27 TRs, ~50s, bottom).** Violin plots indicate the distribution of participants in each condition (coloured circles). White circle, median; horizontal center line, mean; box limits, upper and lower quartiles; whiskers, 1.5x interquartile range. *** p < 0.001, Bonferroni-corrected.


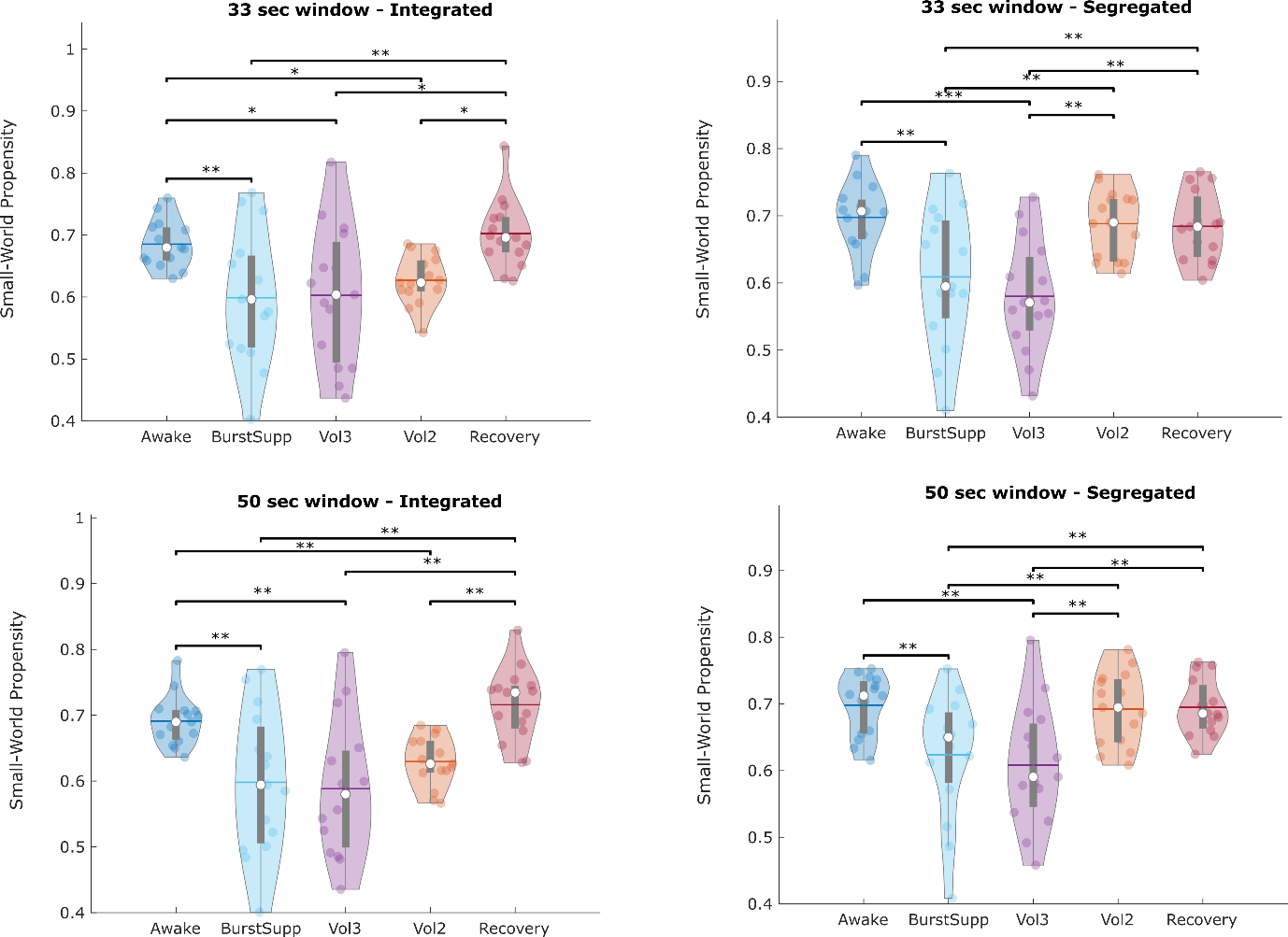


**Supplementary Figure 16. Reduced small-world propensity of dynamic brain networks under sevoflurane, is replicated when using shorter sliding windows (18 TRs, ~33s, top) or longer sliding windows (27 TRs, ~50s, bottom).** Violin plots represent the distribution of small-world propensity for the predominantly integrated sub-state, and the predominantly segregated dynamic sub-state, across levels of sevoflurane. White circle, median; horizontal center line, mean; box limits, upper and lower quartiles; whiskers, 1.5x interquartile range. * *p* < 0.05; ** *p* < 0.01; *** *p* < 0.001, after Bonferroni correction.

**
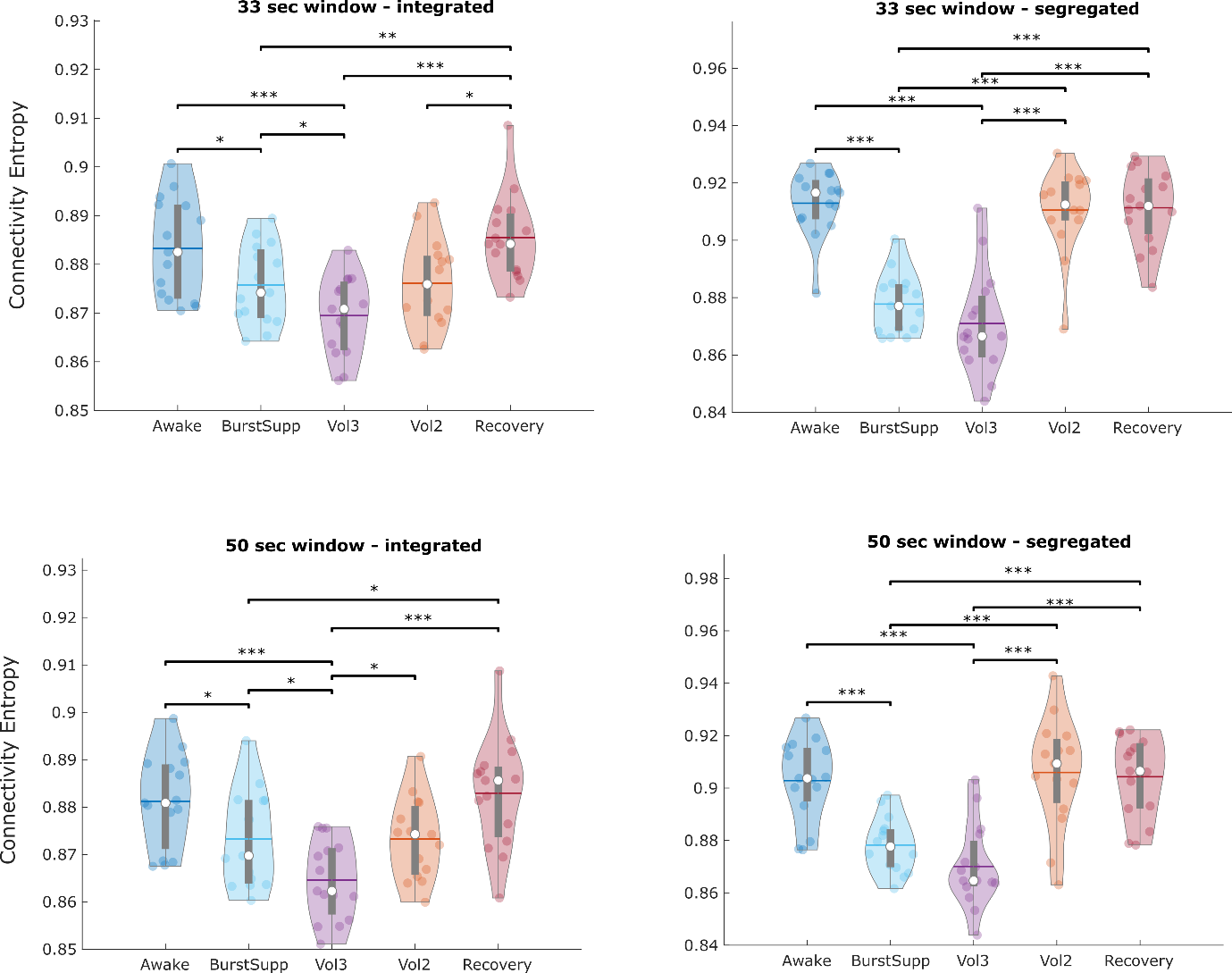
**

### **Supplementary Figure 17. Reduced entropy of functional connections between brain regions under the effects of sevoflurane, is replicated when using shorter sliding windows (18 TRs, ~33s, top) or longer sliding windows (27 TRs, ~50s, bottom).** Violin plots represent the distribution of connectivity entropy across the whole brain for the predominantly integrated sub-state, and the predominantly segregated dynamic sub-state, across levels of sevoflurane. White circle, median; horizontal center line, mean; box limits, upper and lower quartiles; whiskers, 1.5x interquartile range. * *p* < 0.05; ** *p* < 0.01; *** *p* < 0.001, after Bonferroni correction.

# Supplementary Tables

### **Supplementary Table 1.** Statistical results comparing the proportion of time spent in the predominantly integrated sub-state, between each pair of conditions, while controlling for the percentage of scrubbed scans.

| Contrast | Estimate | SE | tStat | EffSize | pValue |
| --- | --- | --- | --- | --- | --- |
| Awake vs BurstSupp | -0.258 | 0.039 | -6.658 | -1.216 | 0.000 |
| Awake vs Vol3 | -0.297 | 0.044 | -6.779 | -1.238 | 0.000 |
| Awake vs Vol2 | 0.043 | 0.053 | 0.819 | 0.150 | 0.420 |
| Awake vs Recovery | -0.067 | 0.055 | -1.234 | -0.225 | 0.228 |
| BurstSupp vs Vol3 | -0.043 | 0.032 | -1.337 | -0.244 | 0.192 |
| BurstSupp vs Vol2 | 0.269 | 0.044 | 6.160 | 1.125 | 0.000 |
| BurstSupp vs Recovery | 0.208 | 0.045 | 4.618 | 0.843 | 0.000 |
| Vol3 vs Vol2 | 0.361 | 0.034 | 10.581 | 1.932 | 0.000 |
| Vol3 vs Recovery | 0.230 | 0.053 | 4.317 | 0.788 | 0.000 |
| Vol2 vs Recovery | -0.120 | 0.066 | -1.813 | -0.331 | 0.081 |

### **Supplementary Table 2.** Statistical results comparing the empirical proportion of time spent in the predominantly integrated sub-state, for each condition, with stationary data generated by a Vector Autoregressive model.

| Contrast | Mean1 | SD1 | Mean2 | SD2 | tStat | df | pValue | EffSize |
| --- | --- | --- | --- | --- | --- | --- | --- | --- |
| Awake | 0.687 | 0.130 | 0.448 | 0.252 | 3.352262 | 14 | 0.005 | 1.150 |
| BurstSupp | 0.427 | 0.085 | 0.420 | 0.109 | 0.198339 | 14 | 0.846 | 0.066 |
| Vol3 | 0.387 | 0.120 | 0.483 | 0.179 | -1.47367 | 14 | 0.163 | -0.604 |
| Vol2 | 0.709 | 0.136 | 0.504 | 0.190 | 3.454662 | 14 | 0.004 | 1.198 |
| Recovery | 0.636 | 0.173 | 0.559 | 0.194 | 0.940582 | 14 | 0.363 | 0.405 |

### **Supplementary Table 3.** Statistical results comparing the empirical proportion of time spent in the predominantly integrated sub-state, for each condition, with a fixed proportion of 50%.

| Contrast | Mean1 | SD1 | Mean2 | SD2 | tStat | df | pValue | EffSize |
| --- | --- | --- | --- | --- | --- | --- | --- | --- |
| Awake | 0.687 | 0.130 | 0.500 | 0.000 | 5.549624 | 14 | 0.000 | 1.953 |
| BurstSupp | 0.427 | 0.085 | 0.500 | 0.000 | -3.3601 | 14 | 0.005 | -1.182 |
| Vol3 | 0.387 | 0.120 | 0.500 | 0.000 | -3.65055 | 14 | 0.003 | -1.285 |
| Vol2 | 0.709 | 0.136 | 0.500 | 0.000 | 5.957174 | 14 | 0.000 | 2.096 |
| Recovery | 0.636 | 0.173 | 0.500 | 0.000 | 3.044845 | 14 | 0.009 | 1.071 |

### **Supplementary Table 4.** Statistical results comparing the small-world propensity of the predominantly integrated sub-state, between each pair of conditions, while controlling for the percentage of scrubbed scans.

| Contrast | Estimate | SE | tStat | EffSize | pValue |
| --- | --- | --- | --- | --- | --- |
| Awake vs BurstSupp | -0.097 | 0.027 | -3.661 | -0.668 | 0.001 |
| Awake vs Vol3 | -0.094 | 0.028 | -3.341 | -0.610 | 0.002 |
| Awake vs Vol2 | -0.042 | 0.014 | -3.066 | -0.560 | 0.005 |
| Awake vs Recovery | 0.017 | 0.014 | 1.242 | 0.227 | 0.225 |
| BurstSupp vs Vol3 | 0.006 | 0.028 | 0.196 | 0.036 | 0.846 |
| BurstSupp vs Vol2 | 0.055 | 0.028 | 2.002 | 0.365 | 0.055 |
| BurstSupp vs Recovery | 0.115 | 0.029 | 3.912 | 0.714 | 0.001 |
| Vol3 vs Vol2 | 0.031 | 0.029 | 1.061 | 0.194 | 0.298 |
| Vol3 vs Recovery | 0.121 | 0.032 | 3.780 | 0.690 | 0.001 |
| Vol2 vs Recovery | 0.067 | 0.021 | 3.167 | 0.578 | 0.004 |

### **Supplementary Table 5.** Statistical results comparing the small-world propensity of the predominantly segregated sub-state, between each pair of conditions, while controlling for the percentage of scrubbed scans.

| Contrast | Estimate | SE | tStat | EffSize | pValue |
| --- | --- | --- | --- | --- | --- |
| Awake vs BurstSupp | -0.087 | 0.024 | -3.696 | -0.675 | 0.001 |
| Awake vs Vol3 | -0.104 | 0.026 | -3.998 | -0.730 | 0.000 |
| Awake vs Vol2 | 0.011 | 0.014 | 0.782 | 0.143 | 0.441 |
| Awake vs Recovery | -0.024 | 0.014 | -1.719 | -0.314 | 0.097 |
| BurstSupp vs Vol3 | -0.015 | 0.026 | -0.562 | -0.103 | 0.579 |
| BurstSupp vs Vol2 | 0.101 | 0.022 | 4.558 | 0.832 | 0.000 |
| BurstSupp vs Recovery | 0.062 | 0.023 | 2.731 | 0.499 | 0.011 |
| Vol3 vs Vol2 | 0.095 | 0.027 | 3.495 | 0.638 | 0.002 |
| Vol3 vs Recovery | 0.087 | 0.027 | 3.200 | 0.584 | 0.004 |
| Vol2 vs Recovery | -0.030 | 0.017 | -1.725 | -0.315 | 0.096 |

### **Supplementary Table 6.** Statistical results comparing the Δ_C_ of the predominantly integrated sub-state, between each pair of conditions, while controlling for the percentage of scrubbed scans.

| Contrast | Estimate | SE | tStat | EffSize | pValue |
| --- | --- | --- | --- | --- | --- |
| Awake vs BurstSupp | 0.133 | 0.059 | 2.246 | 0.410 | 0.033 |
| Awake vs Vol3 | 0.157 | 0.050 | 3.151 | 0.575 | 0.004 |
| Awake vs Vol2 | 0.075 | 0.024 | 3.094 | 0.565 | 0.005 |
| Awake vs Recovery | -0.050 | 0.036 | -1.399 | -0.255 | 0.173 |
| BurstSupp vs Vol3 | 0.020 | 0.057 | 0.349 | 0.064 | 0.730 |
| BurstSupp vs Vol2 | -0.065 | 0.060 | -1.073 | -0.196 | 0.293 |
| BurstSupp vs Recovery | -0.181 | 0.069 | -2.629 | -0.480 | 0.014 |
| Vol3 vs Vol2 | -0.044 | 0.051 | -0.848 | -0.155 | 0.404 |
| Vol3 vs Recovery | -0.226 | 0.063 | -3.611 | -0.659 | 0.001 |
| Vol2 vs Recovery | -0.145 | 0.049 | -2.939 | -0.537 | 0.007 |

### **Supplementary Table 7.** Statistical results comparing the Δ_C_ of the predominantly segregated sub-state, between each pair of conditions, while controlling for the percentage of scrubbed scans.

| Contrast | Estimate | SE | tStat | EffSize | pValue |
| --- | --- | --- | --- | --- | --- |
| Awake vs BurstSupp | 0.103 | 0.066 | 1.560 | 0.285 | 0.130 |
| Awake vs Vol3 | 0.194 | 0.048 | 4.016 | 0.733 | 0.000 |
| Awake vs Vol2 | -0.011 | 0.034 | -0.333 | -0.061 | 0.742 |
| Awake vs Recovery | 0.061 | 0.032 | 1.922 | 0.351 | 0.065 |
| BurstSupp vs Vol3 | 0.088 | 0.057 | 1.543 | 0.282 | 0.134 |
| BurstSupp vs Vol2 | -0.117 | 0.064 | -1.811 | -0.331 | 0.081 |
| BurstSupp vs Recovery | -0.041 | 0.059 | -0.693 | -0.127 | 0.494 |
| Vol3 vs Vol2 | -0.162 | 0.047 | -3.459 | -0.632 | 0.002 |
| Vol3 vs Recovery | -0.152 | 0.048 | -3.150 | -0.575 | 0.004 |
| Vol2 vs Recovery | 0.048 | 0.034 | 1.419 | 0.259 | 0.167 |

### **Supplementary Table 8.** Statistical results comparing the Δ_L_ of the predominantly integrated sub-state, between each pair of conditions, while controlling for the percentage of scrubbed scans.

| Contrast | Estimate | SE | tStat | EffSize | pValue |
| --- | --- | --- | --- | --- | --- |
| Awake vs BurstSupp | -0.055 | 0.026 | -2.079 | -0.380 | 0.047 |
| Awake vs Vol3 | -0.072 | 0.015 | -4.803 | -0.877 | 0.000 |
| Awake vs Vol2 | -0.027 | 0.009 | -3.028 | -0.553 | 0.005 |
| Awake vs Recovery | 0.002 | 0.012 | 0.180 | 0.033 | 0.859 |
| BurstSupp vs Vol3 | -0.016 | 0.024 | -0.660 | -0.120 | 0.515 |
| BurstSupp vs Vol2 | 0.033 | 0.027 | 1.246 | 0.228 | 0.223 |
| BurstSupp vs Recovery | 0.053 | 0.028 | 1.878 | 0.343 | 0.071 |
| Vol3 vs Vol2 | 0.035 | 0.015 | 2.380 | 0.434 | 0.025 |
| Vol3 vs Recovery | 0.080 | 0.019 | 4.153 | 0.758 | 0.000 |
| Vol2 vs Recovery | 0.037 | 0.016 | 2.324 | 0.424 | 0.028 |

### **Supplementary Table 9.** Statistical results comparing the Δ_L_ of the predominantly segregated sub-state, between each pair of conditions, while controlling for the percentage of scrubbed scans.

| Contrast | Estimate | SE | tStat | EffSize | pValue |
| --- | --- | --- | --- | --- | --- |
| Awake vs BurstSupp | -0.019 | 0.034 | -0.569 | -0.104 | 0.574 |
| Awake vs Vol3 | -0.077 | 0.017 | -4.453 | -0.813 | 0.000 |
| Awake vs Vol2 | -0.008 | 0.015 | -0.495 | -0.090 | 0.624 |
| Awake vs Recovery | -0.024 | 0.016 | -1.568 | -0.286 | 0.129 |
| BurstSupp vs Vol3 | -0.056 | 0.030 | -1.892 | -0.345 | 0.069 |
| BurstSupp vs Vol2 | 0.013 | 0.034 | 0.376 | 0.069 | 0.710 |
| BurstSupp vs Recovery | -0.005 | 0.033 | -0.167 | -0.030 | 0.869 |
| Vol3 vs Vol2 | 0.055 | 0.014 | 4.025 | 0.735 | 0.000 |
| Vol3 vs Recovery | 0.060 | 0.017 | 3.487 | 0.637 | 0.002 |
| Vol2 vs Recovery | -0.009 | 0.014 | -0.641 | -0.117 | 0.527 |

### **Supplementary Table 10.** Statistical results comparing the average entropy of connectivity patterns of the predominantly integrated sub-state, between each pair of conditions, while controlling for the percentage of scrubbed scans.

| Contrast | Estimate | SE | tStat | EffSize | pValue |
| --- | --- | --- | --- | --- | --- |
| Awake vs BurstSupp | -0.008 | 0.003 | -2.820 | -0.515 | 0.009 |
| Awake vs Vol3 | -0.015 | 0.003 | -4.644 | -0.848 | 0.000 |
| Awake vs Vol2 | -0.005 | 0.004 | -1.432 | -0.261 | 0.164 |
| Awake vs Recovery | 0.002 | 0.003 | 0.598 | 0.109 | 0.555 |
| BurstSupp vs Vol3 | -0.007 | 0.002 | -2.957 | -0.540 | 0.006 |
| BurstSupp vs Vol2 | 0.001 | 0.003 | 0.272 | 0.050 | 0.788 |
| BurstSupp vs Recovery | 0.010 | 0.003 | 3.121 | 0.570 | 0.004 |
| Vol3 vs Vol2 | 0.007 | 0.003 | 2.157 | 0.394 | 0.040 |
| Vol3 vs Recovery | 0.017 | 0.003 | 5.699 | 1.040 | 0.000 |
| Vol2 vs Recovery | 0.010 | 0.004 | 2.586 | 0.472 | 0.015 |

### **Supplementary Table 11.** Statistical results comparing the average entropy of connectivity patterns of the predominantly segregated sub-state, between each pair of conditions, while controlling for the percentage of scrubbed scans.

| Contrast | Estimate | SE | tStat | EffSize | pValue |
| --- | --- | --- | --- | --- | --- |
| Awake vs BurstSupp | -0.030 | 0.004 | -8.387 | -1.531 | 0.000 |
| Awake vs Vol3 | -0.039 | 0.005 | -7.534 | -1.375 | 0.000 |
| Awake vs Vol2 | 0.006 | 0.005 | 1.124 | 0.205 | 0.271 |
| Awake vs Recovery | -0.006 | 0.005 | -1.083 | -0.198 | 0.288 |
| BurstSupp vs Vol3 | -0.009 | 0.004 | -2.121 | -0.387 | 0.043 |
| BurstSupp vs Vol2 | 0.033 | 0.005 | 7.228 | 1.320 | 0.000 |
| BurstSupp vs Recovery | 0.026 | 0.005 | 5.572 | 1.017 | 0.000 |
| Vol3 vs Vol2 | 0.042 | 0.006 | 6.711 | 1.225 | 0.000 |
| Vol3 vs Recovery | 0.035 | 0.006 | 6.001 | 1.096 | 0.000 |
| Vol2 vs Recovery | -0.009 | 0.007 | -1.329 | -0.243 | 0.195 |
